# Supplementary material for: A diagnostic real-time PCR assay for the rapid identification of the tomato-potato psyllid, Bactericera cockerelli (Šulc, 1909) and development of a psyllid barcoding database
Source: PLoS One. 2020 Mar 26;15(3):e0230741. doi: 10.1371/journal.pone.0230741 (PMC7098582; doi:10.1371/journal.pone.0230741)
Supplement: S1 Table — Optimum primer concentration was 0.2 μM showing the best combination of r2, slope, efficiency, and sensitivity. (DOCX) [file pone.0230741.s001.docx]

| Primer µM | y-intercept | r² | slope | efficiency | LOD ng/µl | C_t_ SD |
| --- | --- | --- | --- | --- | --- | --- |
| 0.1 | 33.137 | 0.977 | -3.314 | 100.336 | 1.0E-05 | 0.334 |
| 0.2 | 29.580 | 0.983 | -3.282 | 106.195 | 1.0E-06 | 0.322 |
| 0.3 | 28.052 | 0.973 | -3.112 | 109.549 | 1.0E-06 | 0.263 |
| 0.5 | 25.989 | 0.981 | -2.661 | 137.535 | 1.0E-04 | 0.180 |
| 1.0 | 26.019 | 0.984 | -2.849 | 124.363 | 1.0E-06 | 0.154 |

**Supplementary Table S1**: Assay performance across a range of primer concentrations at 60 °C and 1.5mM MgCl_2_. Optimum primer conc was 0.2 µM showing the best combination of r^2^, slope, efficiency, and sensitivity.
