# Supplementary material for: A diagnostic real-time PCR assay for the rapid identification of the tomato-potato psyllid, Bactericera cockerelli (Šulc, 1909) and development of a psyllid barcoding database
Source: PLoS One. 2020 Mar 26;15(3):e0230741. doi: 10.1371/journal.pone.0230741 (PMC7098582; doi:10.1371/journal.pone.0230741)
Supplement: S2 Table — (DOCX) [file pone.0230741.s002.docx]

| MgCl_2_ mM | y-intercept | r² | slope | efficiency | C_t_ SD | LOD ng/µl |
| --- | --- | --- | --- | --- | --- | --- |
| 1.5 | 27.992 | 0.981 | -2.952 | 118.126 | 0.219 | 1.00E-06 |
| 3.5 | 27.516 | 0.986 | -2.940 | 118.839 | 0.272 | 1.00E-06 |
| 5.5 | 27.094 | 0.981 | -3.027 | 113.968 | 0.245 | 1.00E-06 |
| 7.5 | 26.911 | 0.981 | -3.007 | 115.070 | 0.351 | 1.00E-06 |
| 9.5 | 26.243 | 0.990 | -2.820 | 126.268 | 0.330 | 1.00E-06 |

**Supplementary Table S2**: Performance of *B. cockerelli* real-time PCR assay at different magnesium chloride (MgCl_2_) concentrations.
