# Supplementary material for: A diagnostic real-time PCR assay for the rapid identification of the tomato-potato psyllid, Bactericera cockerelli (Šulc, 1909) and development of a psyllid barcoding database
Source: PLoS One. 2020 Mar 26;15(3):e0230741. doi: 10.1371/journal.pone.0230741 (PMC7098582; doi:10.1371/journal.pone.0230741)
Supplement: S3 Table — All DNA concentrations tested above the limit of detection (10ng, 1 ng, 0.1ng, 0.01ng 0.001ng, 0.0001ng, 0.00001ng, 0.000001ng) gave 100% positives across 3 x replicates. LOD is given for each temperature. All non-target Bactericera species tested at different DNA concentration gave 0% false positives except for B. albiventris cloned DNA which cross reacted at 64 and 66°C. (*reactions at 64°C gave 33.33% positives at 20 copy numbers). (DOCX) [file pone.0230741.s003.docx]

| Temp °C | y-intercept | r² | slope | efficiency | SD mean | LOD (copy numbers) | *Bactericera albiventris* false positives | |
| --- | --- | --- | --- | --- | --- | --- | --- | --- |
|  |  |  |  |  |  |  | 10ng DNA | 1ng DNA |
| 58 | 28.206 | 0.993 | -3.609 | 89.281 | 0.307 | 2000 | 0% | 0% |
| 60 | 26.425 | 0.991 | -3.489 | 93.478 | 0.296 | 200 | 0% | 0% |
| 62 | 25.397 | 0.990 | -3.401 | 96.799 | 0.234 | 200 | 0% | 0% |
| 64 | 25.162 | 0.984 | -3.435 | 95.481 | 0.199 | 200* | 33.33% | 33.33% |
| 66 | 25.001 | 0.982 | -3.361 | 98.387 | 0.227 | 200 | 33.33% | 33.33% |

**Supplementary Table 3**: Summary of standard curves from optimisation of temperature on Bcoc_JSK2 real-time PCR assay for identification of B. cockerelli. All DNA concentrations tested above the limit of detection (10ng, 1 ng, 0.1ng, 0.01ng 0.001ng, 0.0001ng, 0.00001ng, 0.000001ng) gave 100% positives across 3 x replicates. LOD is given for each temperature. All non-target Bactericera species tested at different DNA concentration gave 0% false positives except for B. albiventris cloned DNA which cross reacted at 64 and 66 °C. (*reactions at 64 °C gave 33.33% positives at 20 copy numbers).
