# Supplementary material for: A diagnostic real-time PCR assay for the rapid identification of the tomato-potato psyllid, Bactericera cockerelli (Šulc, 1909) and development of a psyllid barcoding database
Source: PLoS One. 2020 Mar 26;15(3):e0230741. doi: 10.1371/journal.pone.0230741 (PMC7098582; doi:10.1371/journal.pone.0230741)
Supplement: S4 Table — All treatments showed 100% positives despite small changes to the overall set-up. (DOCX) [file pone.0230741.s004.docx]

| Factor | Combination | | | | | | | |
| --- | --- | --- | --- | --- | --- | --- | --- | --- |
|  | 1 | 2 | 3 | 4 | 5 | 6 | 7 | 8 |
| Cycler | QuantStudio 6 | QuantStudio 6 | QuantStudio 6 | QuantStudio 6 | CFX96 | CFX96 | CFX96 | CFX96 |
| Master mix | unchanged | unchanged | -10% | -10% | unchanged | unchanged | -10% | -10% |
| Primer conc | unchanged | -30% | unchanged | -30% | unchanged | -30% | unchanged | -30% |
| Probe conc | unchanged | -30% | -30% | unchanged | -30% | unchanged | unchanged | -30% |
| Reaction vol | 14 µl | 14 µl | 16 µl | 16 µl | 16 µl | 16 µl | 14 µl | 14 µl |
| Anneal temp. | 61 °C | 59 °C | 61 °C | 59 °C | 59 °C | 61 °C | 59 °C | 61 °C |
| Positives (n=6) | 100% | 100% | 100% | 100% | 100% | 100% | 100% | 100% |
| C_t_ average | 23.81 | 24.21 | 24.11 | 23.98 | 22.25 | 22.33 | 22.25 | 22.57 |

**Supplementary Table S4:** Set-up and results of multifactorial robustness experiment testing the Bcoc_JSK2 assay on *B. cockerelli* genomic DNA. All treatments showed 100% positives despite small changes to the overall set-up.
