# Supplementary material for: A diagnostic real-time PCR assay for the rapid identification of the tomato-potato psyllid, Bactericera cockerelli (Šulc, 1909) and development of a psyllid barcoding database
Source: PLoS One. 2020 Mar 26;15(3):e0230741. doi: 10.1371/journal.pone.0230741 (PMC7098582; doi:10.1371/journal.pone.0230741)
Supplement: Attachment — Submitted filename: doc07744220200113085906.pdf [file pone.0230741.s005.pdf]

all life stages. It can be used as a surveillance and monitoring tool to further study this important crop pest and to aid the prevention of outbreaks, or to prevent their spread after establishment in new areas.

1. ***Introduction***

The psyllid *Bactericera cockerelli* (Šulc, 1909), (commonly known as "Potato Psyllids" or "Tomato-Potato Psyllid"), is a major pest of cultivated Solanaceous crops including potato and tomato [1]. The feeding of this psyllid causes severe damage to potato plants including: deformed tubers; production of numerous small, poor quality tubers; curling of leaves and petioles; and yellowing or purpling of leaves. This leads to stunted growth and loss of yield [2]. *Bactericera cockerelli* is also the main vector of '*Candidatus* Liberibacter solanacearum' (Lso) which is associated with Zebra Chip and psyllid yellows in Central and North America and New Zealand [3–8].

While *B. cockerelli* prefers to complete its life cycle on Solanaceous plants it is also able to reproduce on Convolvulaceae (Bindweeds and Morning Glories) [9]. There may also be an association with *B. cockerelli* and plants in the Lamiaceae (mints and deadnettles) but this is based on incidental observations from glasshouses with high *B. cockerelli* infection [10]. In addition, adult *B.cockerelli* have been found on over 40 species belonging to 20 families, however most of these are either casual, food or shelter plants on which the psyllid is unable to complete a full life cycle and/or transmit Lso [2,10–16]. Four biotypes of *B. cockerelli* have been described according to polymorphisms in the mitochondrial cytochrome *c oxidase subunit I* (COI) gene and represent geographically distinct populations; central, western, north-western, and south-western [17,18]. These populations have been observed to differ in their ability to spread Lso [18,19].

Bactericera cockerelli is thought to originate from South-Western USA and Mexico [2,12] and from here has spread via natural and human-mediated dispersal to extend its range in Northern and Central America and to Oceania where it is now established in New Zealand [20] and more recently Western Australia [21]. In Central America, *B. cockerelli* has been found as far south as Nicaragua [22] but has not currently been found in Costa Rica or Panama [23]. This psyllid has now established small populations in Canada [24] where previously it was not thought to overwinter. After a period of surveillance, in 2017, *B. cockerelli* was found in areas of Western Australia [21], however Lso has not yet been detected in psyllids or plants in these locations. In 2017, all developmental stages of *B. cockerelli* were found in two locations in Ecuador on potato plants. This represents the first finding of *B. cockerelli* in South America [25].

The phloem-limited bacterium '*Candidatus* Liberibacter solanacearum' (Lso) is a pathogen associated with Zebra Chip disease of potatoes [3,26–28] and disease in other Solanaceous crops such as cultivated tomato [1,3,29,30], pepper [31], eggplant [32], tobacco [22,33] and tomatillo [29]. Currently, *B. cockerelli* is the main vector of Lso in field and glasshouse-grown Solanaceous plants in the United States, Mexico, areas of Central America [30–33], Canada [24] and New Zealand [5,6,28]. While 9 haplotypes of Lso have hitherto been described in the literature (A, B, C, D, E, F, G, H, and U) only three of the haplotypes, A, B and F, are associated with disease in Solanaceous plants. Haplotypes A, B, and F are associated with Zebra chip disease in America [3,34,35], whereas only haplotype A has been found in New
Haplotype B is also found in Bactericera maculipennis
Zealand [5,36]. The remaining haplotypes are not vectored by *B. cockerelli* but by closely related species in the Triozidae family. Haplotype G was found in 49 year-old herbarium specimens of *Solanum umbelliferum* [37] but it is not known if it is able to infect potato.

Haplotypes C, D, and E are associated with disease in Apiaceous crops in Europe [27,38–41] and Northern Africa [42,43]. Lso Haplotypes D and E are vectored mainly by *Bactericera trigonica* and to a lesser extent *Bactericera nigricornis* and *Bactericera tremblayi* [41,44,45]. These closely related *Bactericera* spp. are morphologically similar and substantial taxonomic expertise is required to accurately identify species. Currently the geographic distribution of *B. cockerelli* does not overlap with *B. trigonica* and *B. nigricornis* which makes field identification easier. However, in the event of an outbreak of *B. cockerelli* molecular diagnostic methods allow rapid identification by non-specialists and is particularly valuable in areas where other *Bactericera* spp. co-occur.

Haplotype C is found in Northern Europe where it is mainly vectored by *Trioza apicalis* which, together with psyllid feeding, causes up to 100% loss of Apiaceaous crops in Finland [46,47] and Sweden [28] following transmission. Haplotype U is found in asymptomatic *Urtica dioica* (stinging nettle) and is vectored by the psyllid *Trioza urticae* but is not yet known to cause economic damage [48]. It seems that psyllid-mediated infection of host plants with Lso only occurs when the psyllid vector is on its preferred host. Infection of carrot plants with Lso haplotypes vectored by *B. cockerelli* and infection of potato plants by carrot psyllid vectored Lso only occurs very rarely *in-vitro* and suggests that an efficient vector is necessary for wide-scale spread of Lso [49]. Monitoring and prevention of the spread of *B. cockerelli* is essential to prevent the risk of an outbreak of Lso on potato, tomato and other Solanaceous crops in areas where it is not currently found [50].

The impact of *B. cockerelli* and associated Lso transmission on agriculture is significant. Since its arrival in New Zealand circa 2005 via human-mediated dispersal it has caused millions of dollars of economic losses [20]. In 2008, the reduction in exports of capsicum and losses in

To calculate standard curves DNA standards of *B. cockerelli* were prepared using dilution series of linearized cloned plasmid DNA. DNA was extracted as above using the non-destructive method, amplified and cloned into competent *Escherichia coli* cells using the TOPO TA cloning kit (Thermo-Fisher). DNA from successfully transformed colonies was extracted using "PureYield Plasmid Miniprep System" (Promega). For assay validation DNA was cloned from other psyllid species (Supp Tab. S1). Real-time PCRs were performed in 15 µl volumes including: 6.75 µl Jumpstart Taq Ready Mix (Sigma); 1.2 µl $MgCl_2$ (25mM); 0.45 µl of each primer; 0.15 µl probe; 4 µl of molecular grade water (Sigma); and 2 µl of template DNA. Primer concentration, $MgCl_2$ concentration and temperature was adjusted for validation and optimization of the assay as described below. Reactions were performed on a "QuantStudio 6 Flex" (Applied Biosystems) real-time PCR machine and analysis was done on the "QuantStudio Real-Time PCR Software" (Applied Biosystems).

2.5. *Assay validation*

2.5.1. *Specificity*

The final primer and probe set was tested on genomic DNA from 47 *B. cockerelli* specimens from different life stages. These included the 4 US biotypes [17,67] and specimens from New Zealand to determine false negatives. The assay was tested for specificity against genomic DNA of 73 non-target psyllid species collected as mentioned above, to detect false positives. This included a total of 8 other closely related *Bactericera* spp. and the major vectors of Lso on Apiaceous crops (*B. nigricornis*, *B. trigonica* and *Trioza apicalis*). All information on specimens and DNA samples can be found in (Supp Tab. S1). All reactions with non-target psyllid DNA were run in conjunction with a TaqMan Exogenous Internal Positive Control Reagent Kit (Applied Biosystems) to ensure false positives were not

Biosystems) and "CFX Manager 3.1" (BioRad). The methodology used followed the European Network of GMO Laboratories (ENGL) recommendations [68].

3. RESULTS

3.1. DNA extraction, PCR, and DNA sequencing for identification of psyllids

DNA from 110 psyllid specimens comprising 73 different species were extracted, amplified and sequenced successfully from either CO1 or ITS2 gene regions, or (Supp Tab. S1).

Submitted to NCBI? provide accession numbers

3.2. Bioinformatics and Real-Time PCR Assay Design.

While differentiation within both the ITS2 and CO1 gene regions was sufficient to discriminate between psyllid species, the ITS2 gene region was more suitable for TaqMan assay design for *B. cockerelli*. Similarities between CO1 gene sequences between members of the *Bactericera* genus and *B. cockerelli* were higher than in the ITS2 region (average % similarity = 82.51 ± 0.68 for CO1 and 77.80 ± 4.79 for ITS2) (Tab. 1). The ITS2 region showed larger sections of variability along the gene on which to design primers and probes. Several primer and probe sets passed the selection criteria, but most were unsuitable due to high rate of false positives from closely related *Bactericera* species. The final primer and probe set Bcoc_JSK2 (Tab. 2) targets a 187bp region of the ITS2 gene (Fig. 1).

Species	ITS2			CO1		
	% Similarity	bp	GC content %	% Similarity	bp	GC content %
B. trigonica	78.96	662	59.3	82.88	509	35.4
B. tremblayi	79.16	665	59.1	82.97	682	33

160725.B.coc.06.col.04	transformed *E. coli*	Lab	11.78	6	100	/	TBC	Cloned, 10ng
160725.B.coc.07.col.08	transformed *E. coli*	Lab	11.67	6	100	/	TBC	Cloned, 10ng

Table 3: Information on *Bactericera cockerelli* samples tested with Bcoc_JSK2 assay including genomic DNA from adults, immatures, single eggs and egg batches. Location of samples collection is also included. All samples gave 100% positives with the assay, samples were run in at least duplicate. GenBank accession numbers relating to sequenced CO1 and ITS2 regions are included.

Figure 1. CLUSTAL-W alignment of ITS2 regions from closely related *Bactericera* species showing variable regions and the gene target for the Bcoc_JSK2 primer and probe set. Bases shades with black show differences to *B. cockerelli* sequence. Colour highlights locations of forward primer (blue highlight); reverse primer (green highlight) and probe (yellow highlight). The probe and reverse primer are reverse compliments of the highlighted regions here.

3.3. Specificity and Sensitivity

This assay did not amplify DNA from any of the 73 non-target psyllid species when tested at 60 °C with primer concentration 0.2 µ/mol. This included 8 closely related *Bactericera* species with similar ITS2 and CO1 sequences (Tab. 1). Under optimal conditions, false negatives = 0% for all non-target species tested with pure genomic DNA, giving a diagnostic specificity of 100%. Some suboptimal reaction conditions showed 33% false positives against high concentrations (10 ng / 1 ng) of *Bactericera albiventris* cloned DNA as mentioned below. All *B. cockerelli* genomic DNA samples gave positive results (Tab. 3) giving 0% false negatives across 54 biological replicates and 147 technical replicates; resulting in a diagnostic sensitivity of 100%. These included *B. cockerelli* specimens from each of the four US biotypes as well as specimens from New Zealand. These specimens included adults,

immatures and eggs. The assay can amplify *B. cockerelli* DNA from both cloned and genomic samples. Under optimal conditions for PCR efficiency and specificity (60 °C, 0.2 µM primer, 1.5 mM $MgCl_2$) the limit of detection was 0.00001 ng DNA across a range of different reaction parameters this equates to 200 copy numbers of ITS2 calculated using the following equation: Number of Copies = (ng DNA x 6.022×10^{23}) ÷ (length of gene region in base pairs * 1×10^{9} * 660). The copy number calculator available at http://scienceprimer.com/copy-number-calculator-for-realtime-pcr was used. Diagnostic sensitivity was 100% on all DNA extracted from *B. cockerelli* immatures. False negatives from DNA from egg extractions were 0% for single eggs and 0% for batches of 3 and 10 eggs.

3.4. <u>Repeatability and Reproducibility</u>

No significant differences were found between C_t means across the different replicates at different concentrations as tested by two-way ANOVA ($F_{5, 25}$= 0.54, p = 0.955). The assay also performed consistently across different machines and there was no significant difference between runs across the two machines as tested by two-way ANOVA ($F_{1, 5}$= 1.28, p = 0.279).

3.5. <u>Robustness/Optimization</u>

The assays amplified *B. cockerelli* DNA at all primer concentrations, $MgCl_2$ concentrations and annealing temperatures with varying levels of efficiency, precision, and sensitivity (Supp Tabs. S2-S4). At primer concentration, 0.5 µM the assay was less sensitive only amplifying up to 0.001 ng DNA. At higher primer concentrations (0.5 and 1.0) the assay showed higher sensitivity, but efficiency was outside the range for acceptable use. The assay performed optimally at 0.2 µM primer concentration showing good efficiency and high sensitivity

different treatments and was shown to be robust and unaffected by small changes in assay set-up (Supp Tab. S5). Each treatment gave 100% positives for amplification of *B. cockerelli* genomic DNA.

4. **Discussion**

lots of repeating. consider a combined results & discussion

We have designed and validated the first species-specific, qualitative real-time PCR TaqMan assay for *B. cockerelli* by using the comparison of 73 non-target species to identify unique gene regions that were suitable for primer/probe design and species differentiation. The assay was also tested on 9 closely related *Bactericera* species. The number of species used in our study is relatively high compared to other reported TaqMan assays for plant pests that report lower numbers of non-target species [69,70]. The ITS2 region was found to be suitable for assay design as it contained high intraspecific variation consisting of stretches of insertions and deletions (INDELs), which are ideal diagnostic sites for primer and probe design. Although COI is classically used for species discrimination, mutations were less clustered which is likely due to third base degeneracy. The ITS2 region can also be used to distinguish species phylogenetically and has been used to identify cryptic species in the *Cacopsylla pruni* complex [60]. The sequences obtained from this study will improve the current species representation on online DNA databases, reducing the chance of Type II errors (i.e. misidentification due to lack of conspecific references) [71]. It is important to note that the target and non-target specimens consisted of multiple samples of each species from different geographical regions. This reduced the risk of false negatives that may arise if during assay design, the target specimens were obtained from a limited geographic spread and were therefore not representative of the genetic variation within the species. To

account for interspecific variation, *B. cockerelli* samples used in the assay validation consisted of specimens from the four USA biotypes and specimens from New Zealand.

Species identification can be achieved for *B. cockerelli* by sequencing the ITS2 or COI regions as both loci, to date, have entries in the National Centre for Biotechnology Information (NCBI) database. However, DNA sequencing is a lengthy process compared to real-time PCR, which in contrast is a rapid identification method involving fewer pieces of equipment, reagents, and time both for running the sample, and processing it digitally. The success rates of eradications are dependent on the length of time between introduction, detection, and implementation of eradication measures as Lso displays a short transmission time from *B. cockerelli* to potatoes [4,28]. Feasibly, methodology described in this study could be used to extract DNA from a specimen and test for *B. cockerelli* positives within 12 hrs or quicker.

The assay described in this study can identify *B. cockerelli* specimens from all life stages. It is robust, reliable and can detect 200 copies (0.00001 ng DNA) of the ITS2 gene region. This is the first assay which uses a TaqMan real-time PCR module to specifically identify this important pest species. The assay performs well across a range of annealing temperatures, $MgCl_2$ concentrations and primer concentrations and is a robust tool that can be used to give reliable results despite human error, different lab practices, equipment, standard operating procedures or PCR set-ups.

The assay can be used on eggs and immatures, as well as adults of *B. cockerelli,* which will enable the rapid identification of this species from specimens that may be otherwise impossible to identify. For example, identification of eggs of *B. cockerelli* is not possible using classical taxonomy methods alone and it is now possible to rapidly identify this cryptic life stage. In addition, adult females and immatures are considerably more difficult to

identify compared to adult males. The genus *Bactericera* currently contains 160 species [56] and <1% of these have been tested in the current study due to the difficulty obtaining other specimens from the field or lab colonies. However Europe is home to 26 different species of Bactericera 30% of which have been validated using this assay [56]. This assay has been tested against the *Bactericera* species which are most commonly found in potato and carrot fields in Europe and the wider EPPO region which should minimize the potential for false positives and ensure the assay is efficient at detecting outbreaks in European fields.

The ability to identify insects to species accurately and quickly is essential to support national biosecurity measures for the prevention and early detection of new pests and vectors of disease. Psyllids can be difficult to identify and certain species of concern, such as *B. cockerelli,* require skilled specialist taxonomists to accurately differentiate between closely related species that may or may not be of risk to plant health. Entomologists with the skills required to do so are a valuable yet sparse resource and incorrect identification of a psyllid species could lead to a delay in national response or inappropriate allocation of resources to resolve a problem that is not present, possibly leading to the unnecessary destruction of crops. We have designed a functional tool for reliable, rapid, robust and accurate identification of the tomato-potato psyllid *B. cockerelli* to support national biosecurity measures should this pest be found within the EPPO region or other regions where it could become damaging to agriculture. The situation in New Zealand and recent interceptions of *B. cockerelli* on aubergine in plant material imported into the UK from Mexico highlight the risk and pathways for introduction into new areas [54]. Furthermore immature stages of *B. cockerelli* are commonly intercepted on vegetable material from the Western-United States and Mexico and adults are occasionally found on leafy vegetables

such as lettuce [54]. Recent studies suggest *B. cockerelli* can utilize multiple host-plants within the Solanaceae and other plant families; the potential for eggs and immatures of *B. cockerelli* to be present on yet undetermined host-plants is likely.

The identification of psyllids using classical taxonomy is often aided using information about the host-plant on which the specimen was found [72]. However, although psyllids are typically monophagous, some species feed on a few closely related plant species. For example, northern hemisphere pest species, such as *B. cockerelli* and *B. trigonica,* feed on several plant species within a family [73]. In the case of *B. cockerelli* its range of host-plants (reproductive, food and incidental) is much wider than originally thought [9,16,74]. Adult psyllids may also settle on plants that they do not feed on, known as a casual plant [75]. It is possible that: A) *B. cockerelli* can utilise other host-plants and could be overlooked; B) other psyllid species morphologically/ecologically similar to *B. cockerelli* could be mistaken for this pest if only considering the host plant on which they are found. Therefore, the identification of suspected host-plant material alone cannot be reliably used for psyllid identification and for most psyllid species their host-plant range is unknown or fragmentary at best. This assay can be used to study life parameters of *B. cockerelli* in the field such as oviposition on reproductive host-plants, as eggs can now be identified.

Additionally, host-plant data is sometimes unavailable depending on the trapping method employed. For example, indirect host-plant data is available from trapping methods at ground level (e.g. water/sticky traps) with less data available for insects caught beyond the vegetation layer (e.g. 12.2m suction traps or aerial balloon traps). Unlike spot checks performed by inspectors directly on plant produce, canopy-level or aerial traps which

provide data at field or regional level [76,77]. A diagnostic assay for *B. cockerelli* is therefore useful for monitoring its occurrence at several spatial scales, from local border checks to regional surveys. As some collection methods obtain specimens where host-plant data is not available, this tool enables rapid screening of psyllids from suction-traps or those collected in sticky traps, pan traps or similar. However, further validation should be performed to ensure the assay performs adequately on *B. cockerelli* fragments obtained from these kinds of traps. The assay can also be used in areas currently known to have *B. cockerelli* to easily quantify numbers from the field or to monitor migration, distribution and spread of this pest species.

Further validation of this assay however should include its use in several different laboratories with different practitioners. Different reaction mixes should be tested for their suitability with this assay and its compatibility with alternative qPCR machines. It is also valuable to test these primers in a set-up using an inter-collating dye such as Sybr Green. Additionally, this assay should be tested on further samples of *Bactericera* and other closely related Triozidae psyllids. Further validation of this assay should be performed on more *Bactericera* species not included in this study and on DNA from Solanaceous host plants. This will ensure false positives are not obtained from DNA extracted from complex matrices. Due to being based on real-time PCR chemistry, one limitation of this assay is that it cannot be taken out into the field, making it less portable than a LAMP assay or other NGS sequencing techniques such as Nanopore technology.

this should be done now, plant DNA can be amplified from psyllids

In conclusion a rapid, specific, robust, repeatable and reliable real-time PCR assay has now been validated and can be used to detect the important pest *B. cockerelli*. This work has
