# Supplementary material for: A diagnostic real-time PCR assay for the rapid identification of the tomato-potato psyllid, Bactericera cockerelli (Šulc, 1909) and development of a psyllid barcoding database
Source: PLoS One. 2020 Mar 26;15(3):e0230741. doi: 10.1371/journal.pone.0230741 (PMC7098582; doi:10.1371/journal.pone.0230741)
Supplement: Attachment — Submitted filename: PONE-D-19-35047_R1_reviewer.pdf [file pone.0230741.s007.pdf]

PLOS ONE

A diagnostic real-time PCR assay for the rapid identification of the tomato-potato psyllid, Bactericera cockerelli (Šulc, 1909) and development of a psyllid barcoding database.

--Manuscript Draft--

Manuscript Number:	PONE-D-19-35047R1
Article Type:	Research Article
Full Title:	A diagnostic real-time PCR assay for the rapid identification of the tomato-potato psyllid, Bactericera cockerelli (Šulc, 1909) and development of a psyllid barcoding database.
Short Title:	Bactericera cockerelli diagnostic assay
Corresponding Author:	Jason Charles Sumner-Kalkun, Ph. D.<br>Science and Advice for Scottish Agriculture<br>Edinburgh, UNITED KINGDOM
Keywords:	Bactericera cockerelli, Liberibacter, psyllids, pest insects, plant pathology, molecular biology, real-time PCR, TaqMan Assay, crop protection, phytosanitary, diagnostics, vector, biosecurity,
Abstract:	The accurate and rapid identification of many insect pests is an important step in the prevention and control of outbreaks in areas that are otherwise pest free. The potato-tomato psyllid Bactericera cockerelli (Šulc, 1909) is the main vector of ' Candidatus Liberibacter solanacearum' on potato and tomato crops in Central and Northern America and New Zealand. This study describes the design and validation of the first species - specific TaqMan probe-based real-time PCR assay, targeting the ITS2 gene region of B. cockerelli . The assay successfully detected B. cockerelli genomic DNA from adults (100% accuracy, n=72); immatures (100% accuracy, n=26) and eggs (100% accuracy, n=25) . This assay also detected DNA from cloned plasmids containing the ITS2 region of B. cockerelli (100% accuracy, n=24). The assay showed 0% false positives when tested on genomic and cloned DNA from 73 other psyllid species collected from across Europe, New Zealand and Mexico. This included 8 other species in the Bactericera genus and the main vectors of ' Candidatus Liberibacter solanacearum' worldwide. The limit of detection for this assay at optimum conditions was 0.000001ng DNA (~200 copies) of ITS2 DNA which equates to around a 1:10000 dilution of DNA from one single adult specimen. This assay is the first real-time PCR based method for accurate, robust, sensitive and specific identification of B. cockerelli from all life stages. It can be used as a surveillance and monitoring tool to further study this important crop pest and to aid the prevention of outbreaks, or to prevent their spread after establishment in new areas.
Order of Authors:	Jason Charles Sumner-Kalkun, Ph. D.
	MJ Sjölund
	YM Arnsdorf
	M Carnegie
	F Highet
	D Ouvrard
	AFC Greenslade
	JR Bell
	R Sigvald
	DM Kenyon
Response to Reviewers:	Dear Dr Sean Michael Prager,<br>Please find enclosed our revised manuscript entitled "A diagnostic real-time PCR assay for the rapid identification of the tomato-potato psyllid, Bactericera cockerelli (Šulc, 1909) and development of a psyllid barcoding database". We thank you and the

Powered by Editorial Manager® and ProduXion Manager® from Aries Systems Corporation

reviewers for your careful review of our submitted manuscript and the opportunity to resubmit an improved version. We find the comments to be fair and constructive and have helped to improve the final manuscript. Please see below our responses to points raised by the reviewer's comments and the amendments we have made to the final manuscript. We provide a copy of the resubmitted manuscript with track changes and track changes accepted. Line numbers refer to those given in the resubmitted manuscript with track changes accepted.
We hope that you will consider this revised manuscript of a high enough standard to be published in PLOS ONE.
Yours Sincerely,
Dr Jason C Sumner-Kalkun
(on behalf of all co-authors)

•Thank you for considering our work for publication in your journal. We found all reviewer comments to be useful and constructive and appreciate you overall assessment of the manuscript. We are pleased to have the opportunity to resubmit an improved version. We have made considerable efforts to condense the introduction and discussion section to include only relevant information and to streamline the manuscript. We agree that there was some duplication and repetition in the discussion, and it has been re-written accordingly. We decided, due to the technical nature of the paper that a separate results and discussion section was more appropriate. We have addressed the reviewer concerns regarding data availability and all sequence data has been uploaded to GenBank and is now free to be made publicly available. We hope that our amendments are deemed adequate to meet the high standards of PLOS ONE and are excited about the possibility of publishing with you.

REVIEWER COMMENTS TO AUTHOR
Reviewer #1: This manuscript describes a qPCR assay to identify potato psyllid intercepted in shipments. The assay is paramount to Europe's ability to detect potential introductions of this psyllid, which would be harmful to agricultural production. The authors describe the assay and confirmed that it does not amplify the ITS gene of other psyllids. My major concern for the manuscript is that it is overwritten and over-interpreted. The study is very simple - qPCR assay to detect potato psyllid - yet the text is over 50 pages long, includes unrelated information in the introduction, and includes an overly long discussion. The manuscript should be re-written to focus only on the assay and its use in trade commodities. Specific comments are provided in an attached document. I will apologize for my handwriting.
MAJOR POINTS
We appreciate your thorough assessment of our manuscript and thank you for your time. We found your comments very constructive and helpful. We have taken the care to reduce the introduction and discussion sections considerably to provide more focus on the assay and its uses, removing a lot of the duplication. The manuscript has been edited down to 31 pages + supplementary material. We attempted to produce a combined results and discussion section but felt that, due to the technical nature of the paper, keeping these separate was preferable. We hope that you will agree with this assessment on reading the improved version.
On the recommendation of the reviewer on line 486 of the previous manuscript we have performed the assay on Potato DNA to check for cross-reaction. No false positives were obtained from 8x reps of 3 Potato samples "Maris Piper" variety.
MINOR POINTS
1.Line 45: Abstract overwritten, stats to be removed, word count reduced
-The abstract Line 21-39 has been reduced in size with all stats removed and is now within the word limit (252 words)
2.Line 47: Remove "-" in "Potato-Psyllids"
-Changed to "Potato Psyllid" now line 41
3.Line 49: "The feeding of…." To be changed to "Feeding by"
-Changed as suggested now line 43
4.Line 53: Psyllid yellows refers to the feeding damage described above.
-Removed to avoid confusion and improve accuracy. Line 47
5.Lines 55-56: Change "…is also able to reproduce on…" to "…can also complete development on species of…."
-Changed as suggested line 49-50
6.Lines 56-58: Statement not deemed true
-Statement removed line 51

Powered by Editorial Manager® and ProduXion Manager® from Aries Systems Corporation

7.Line 61: Remove statement on Lso transmission to non-host plants of B. cockerelli
-Statement removed line 53
8.Line 64-65: Remove claims about B.cockerelli populations observed to differ in their ability to spread Lso
-Changed to: “Evidence suggests that these genetic types may differ in their ability to spread Lso…” Lines 56-57
9.Line 86: Haplotype B is also found in Bactericera maculipennis
-Information added to the text line 71
10.Line 111: typo capsicum not italics
-Changed to “…Capsicum…” line 84
11.Line 223:This table is referenced a lot, make it a real table
-Supp Tab. S1 now changed to Table 1. In results section Line 244-250. Cited on lines: 244. Supp Tabs 2-4 renumbered to Supp Tabs 1-3 and Tables 1-3 renumbered to Tables 2-4.
12.Line 276: Submitted to NCBI? Provide accession numbers
-Accession numbers added to Table 1. Lines 246-252 and Table 4. Lines 271-276
13.Line 314-315: change “….cloned DNA as mentioned below.” To “..DNA below”.
-Changed to “….cloned DNA (see below).” Line 290
14.Line 319: change “immatures” to “nymphs”
-The term “immatures” is preferred by leading psyllid taxonomists Daniel Burckhardt and David Ouvrard, that latter of whom is an author on this paper. See ref: (Burckhardt et al. 2014). We have kept the term “immatures” or “immature life stages” throughout.
Burckhardt D, Ouvrard D, Queiroz D, Percy D (2014) Psyllid Host-Plants (Hemiptera: Psylloidea): Resolving a Semantic Problem. Florida Entomol 97:242–246 .
https://doi.org/10.1653/024.097.0132
15.Line 411: “…Bactericera…” to be italicised
-Changed to italics. Line 354
16.Lines 439-441: Section to be re-written as inaccurate wording used
-This section was removed in the re-write of the discussion.
17.Line 468: Suggestion to perform further validation on Solanaceous DNA
-3 x samples of Solanum tuberosum ‘Maris Piper’ were tested and were negative results added to lines: 194-196 and 284-285. Also results of primer blast etc. did not return any hits for Solanum species or any plant sequences.
Reviewer #2
-We are thankful to the reviewer for their detailed and careful examination of our paper. They have provided very useful, constructive comments regarding the technical aspects of the paper and have informed us of errors in the finer details. We hope we have incorporated changes to their satisfaction, and we have endeavoured to clear up the technical details that were missing or incorrect.

1.Data availability
-Psyllid DNA sequences have been uploaded to GenBank and accession numbers are provided in Tab1. And Tab4; lines 246-252 and 271-276 respectively.
2.Page 8 Line 163: What part of the body is used for micro-dissection to extract DNA? The authors should describe the micro-dissection procedure in more detail rather than only citing the papers.
-The non-destructive DNA extraction method is described on lines 121 – 132. “Micro-dissection” was used here to describe the piercing of the abdomen and thorax. “Micro-dissection” has been changed to “pierced” as a more appropriate term (line 126).
3.Page 8 Line 172: “For amplification of ITS2 primers CA55p8sFcm-F and CA28sB1d-R [60] and for amplification of CO1 gene regions arthropod barcoding Primers LCO1490 and HCO2198 [61].” The authors should check the grammar here. It is not a complete sentence. It could be “For amplification of ITS2, primers CA55p8sFcm-F and CA28sB1d-R [60] were used, and for amplification of CO1 gene regions, arthropod barcoding Primers LCO1490 and HCO2198 [61] were used.”
-Changed as suggested lines 135- 137
4.Page 10 Line 204: “DNA was extracted as above using the non-destructive method, amplified and cloned into competent Escherichia coli cells using the TOPO TA cloning kit (Thermo-Fisher).” The authors should specify what genes (ITS2 or CO1?) they amplified for cloning, and what restriction enzyme (EcoRI?) they used to linearize the plasmid.
-Information added and moved from later section 2.5.2 Sensitivity. Now line 171-178
5.Page 10 Line 212: The authors need to list the real time PCR cycling conditions here, for example XX degrees for XX seconds.

Powered by Editorial Manager® and ProduXion Manager® from Aries Systems Corporation

-Added lines 178-181

6.Page 10 Line 223: “All reactions with non-target psyllid DNA were run in conjunction with a TaqMan Exogenous Internal Positive Control Reagent Kit (Applied Biosystems) to ensure false positives were not obtained due to inhibition within the reaction”. Here, “ensure” should be “rule out the possibility that”

- Changed as suggested lines 196-201

7. Page 11 Line 226: “DNA from all non-target psyllids was sequenced to ensure psyllid DNA was present in all reactions to rule out false negatives due to inefficient DNA extraction.” What DNA was sequenced? PCR product from ITS2 or CO1? The authors need to specify

- Details now added to new Tab 1 and citation to table included on lines 246-252

8. Page 11 Line 239: “6 subsequent dilutions were made. Stock DNA 10 ng/μl was linearised using EcoRI restrictions enzyme (New England Biolabs),” Here “6 subsequent dilutions” should be “8 subsequent dilutions”, according to the nine point 10-fold dilution series mentioned on Page 11 Line 236.

- Corrected Line 212-213

9. Page 12 Line 252: “A six point 1:10 dilution series starting at 10ng/μl was used with each dilution being performed in triplicate.” Here, “six point” should be “nine point” according to Page 11 Line 236.

- Only 6 points were used for repeatability. This is sufficient to analyse standard curves between runs. Lines 222-223 refer to sensitivity experiments only.

10. Page 12 Line 263: “For each tested parameter, optimization was performed across an eight point 1:10 dilution series starting at 10ng DNA.” Here, “eight point” should be “nine point”, “10ng” should be “10ng/μl”.

- Corrected. Line 234

11. In Supplementary table S1, green and red color coding should be explained in the text. What does TBC mean? Accession numbers should be given for all the sequences. Accession numbers in Table 3 should also be given and TBC should be explained.

- We apologise for this error; this colouring has been removed as was an artefact of preparing the table and shouldn’t have been included in the submitted version. TBC was used to show we were waiting for accession numbers. Accession numbers are now added to tables and TBC removed. Tab. 1 lines: 246-247 Tab.4 lines:

12. Page 14 Line 289: “CO1 genes showed higher similarity and generally less conserved and variable regions compared to ITS2 regions.” Here “less conserved and variable” should be “less variable”.

- Corrected line 266

13. Page 17 Line 310: “0.2 μ/mol” should be “0.2 μM”.

- Corrected line 285

14. Page 18 Line 324: “The copy number calculator available at http://scienceprimer.com/copy-number-calculator-for-realtime-pcr was used.” Here a hyperlink should be created. According to the link and the formula given, 0.00001ng DNA equals 4.879×10000 copies, if length of gene region is considered 187bp (product length of ITS2 in real time PCR). However, the authors calculated that it equals to 200bp. Please double check the calculation.

-Limit of detection is actually 0.000001 ng DNA. This mistake of 10 fold higher amounts stated in the text was found throughout and in tables. We have now corrected them.

The correct equation should be:

Number of Copies = (ng DNA(0.000001) x 6.022x1023) ÷ ((length of plasmid 4656bp + cloned fragment 700bp) * 1x109 * 660) = 170.36 copy numbers.

15. Page 18 Line 337: “At primer concentration, 0.5 μM the assay was less sensitive only amplifying up to 0.001 ng DNA.” It should be “At primer concentration 0.5 μM, the assay was less sensitive only amplifying up to 0.001 ng DNA.”

-Corrected. Lines 313-314

16. Page 18 Line 338: “At higher primer concentrations (0.5 and 1.0) the assay showed higher sensitivity” Here “(0.5 and 1.0)” should be “(1.0 μM)”.

-Corrected. Line 314

17. Page 19 Line 350: “The precision of the assay was lower at higher MgCl2 concentrations 6mM and 8mM (Supp Tab. S3).” Here “6mM and 8mM” should be “7.5mM and 9mM”.

-Corrected. Lines 326-327

18. Page 19 Line 354: “Reactions at 58 °C were 10 to 100-fold less sensitive than reactions at 58 °C.” Here it should be “Reactions at 58 °C were 10 to 100-fold less sensitive than reactions at 64 °C.”

Powered by Editorial Manager® and ProduXion Manager® from Aries Systems Corporation

-Corrected. Lines 330-331
19.Page 20 Line 367: “We have designed and validated the first species-specific, qualitative real-time PCR TaqMan assay for B. cockerelli by using the comparison of 73 non-target species to identify unique gene regions that were suitable for primer/probe design and species differentiation.” Here “qualitative” should be “quantitative”.
-Changed to quantitative. Line 351

Reviewer #3
-We thank the reviewer for their thoughtful assessment of our manuscript and are pleased that only minor corrections were found throughout. The corrections have improved the manuscript greatly and have ironed out some important technical errors. We hope that our amendments are deemed satisfactory and have covered the issues they have raised.

1.Page 8 line 175: please replace amount of primers used with final concentration of primers (or add this)
- Added. Line 138
2. Page 10 line 213: please add cycling conditions of real time PCR, as done for CO1 and ITS2 amplification
-Added lines 178-181
3. Table 1: should include also B. cockerelli, so to include fragment size of amplicons for this species. In alternative, fragment sizes can be added to the main text
-B. cockerelli added to table 2. Line 262-263
4. Table 3: not clear what the "/" symbol in the CO1 column means
-Samples with / were not amplified in this region. Accession numbers for each sample have been added and this is explained better in the text. Lines: 252 Tab.1 ; 276 Tab. 4
5. Page 17 line 310: please check spelling of concentration
-Corrected to µM. Line 285
6. Page 17 line 316: numbers seem not to add up: how many technical replicates were used per sample?
-Information on technical reps is incorporated into table 4. Some samples were tested in duplicate, triplicate or 6x replicates.
7. Page 18 line 323: I have tried the formula myself using the concentration (0.00001 ng) and fragment size (187 bp) specified by the authors, but I get a quite different number of ITS2 copies (about 50,000 versus 200). Please double check, and add actual numbers to the formula.
- Limit of detection is actually 0.000001 ng DNA. This mistake of 10-fold higher amounts stated in the text was found throughout and in tables. We have now corrected them. The correct equation should be:
Number of Copies = (ng DNA(0.000001) x 6.022x1023) ÷ ((length of plasmid 4656bp + cloned fragment 700bp) * 1x109 * 660) = 170.36 copy numbers.
8. Of some concern is the author's answer to the data accessibility question. Authors stated that they are not going to make all data available, with a generic "Some restrictions will apply". Please explain what data will not be made accessible and why.
- All data will be made available. Accession numbers were not available at the time of submission as they were restricted by one or more of our projects until we had consent to upload them to public databases.

Additional Information:

Question	Response
**Financial Disclosure**<br><br>Enter a financial disclosure statement that describes the sources of funding for the work included in this submission. Review the submission guidelines for detailed requirements. View published research articles from *PLOS ONE* for specific examples.	DK: This work was supported by the EU Horizon2020 Programme under grant agreement No. 635646, POnTE (Pest Organisms Threatening Europe) https://ec.europa.eu/programmes/horizon2020/en and the Scottish Government [RRL/001/14].<br>JB: The Rothamsted Insect Survey, a National Capability, is funded by the Biotechnology and Biological Sciences Research Council under the Core Capability Grant BBS/E/C/000J0200. https://bbsrc.ukri.org/funding/<br>The funders had no role in study design, data collection and analysis, decision to publish, or preparation of the manuscript.

Powered by Editorial Manager® and ProduXion Manager® from Aries Systems Corporation

This statement is required for submission and **will appear in the published article** if the submission is accepted. Please make sure it is accurate.<br><br>**Unfunded studies**<br>Enter: *The author(s) received no specific funding for this work.*<br><br>**Funded studies**<br>Enter a statement with the following details:<br>• Initials of the authors who received each award<br>• Grant numbers awarded to each author<br>• The full name of each funder<br>• URL of each funder website<br>• Did the sponsors or funders play any role in the study design, data collection and analysis, decision to publish, or preparation of the manuscript?<br>• **NO** - Include this sentence at the end of your statement: *The funders had no role in study design, data collection and analysis, decision to publish, or preparation of the manuscript.*<br>• **YES** - Specify the role(s) played.<br><br>* typeset	
**Competing Interests**<br><br>Use the instructions below to enter a competing interest statement for this submission. On behalf of all authors, disclose any competing interests that could be perceived to bias this work—acknowledging all financial support and any other relevant financial or non-financial competing interests.<br><br>This statement **will appear in the published article** if the submission is accepted. Please make sure it is accurate. View published research articles from *PLOS ONE* for specific examples.	The authors have declared that no competing interests exist.

Powered by Editorial Manager® and ProduXion Manager® from Aries Systems Corporation

**NO authors have competing interests**<br><br>Enter: *The authors have declared that no competing interests exist.*<br><br>**Authors with competing interests**<br><br>Enter competing interest details beginning with this statement:<br><br>*I have read the journal's policy and the authors of this manuscript have the following competing interests: [insert competing interests here]*<br><br>* typeset	
**Ethics Statement**<br><br>Enter an ethics statement for this submission. This statement is required if the study involved:<br><br>• Human participants<br>• Human specimens or tissue<br>• Vertebrate animals or cephalopods<br>• Vertebrate embryos or tissues<br>• Field research<br><br>Write "N/A" if the submission does not require an ethics statement.<br><br>General guidance is provided below. Consult the submission guidelines for detailed instructions. **Make sure that all information entered here is included in the Methods section of the manuscript.**	N/A

Powered by Editorial Manager® and ProduXion Manager® from Aries Systems Corporation

**Format for specific study types**<br><br>**Human Subject Research (involving human participants and/or tissue)**<br>• Give the name of the institutional review board or ethics committee that approved the study<br>• Include the approval number and/or a statement indicating approval of this research<br>• Indicate the form of consent obtained (written/oral) or the reason that consent was not obtained (e.g. the data were analyzed anonymously)<br><br>**Animal Research (involving vertebrate animals, embryos or tissues)**<br>• Provide the name of the Institutional Animal Care and Use Committee (IACUC) or other relevant ethics board that reviewed the study protocol, and indicate whether they approved this research or granted a formal waiver of ethical approval<br>• Include an approval number if one was obtained<br>• If the study involved *non-human primates*, add *additional details* about animal welfare and steps taken to ameliorate suffering<br>• If anesthesia, euthanasia, or any kind of animal sacrifice is part of the study, include briefly which substances and/or methods were applied<br><br>**Field Research**<br><br>Include the following details if this study involves the collection of plant, animal, or other materials from a natural setting:<br>• Field permit number<br>• Name of the institution or relevant body that granted permission	
**Data Availability**<br><br>Authors are required to make all data underlying the findings described fully available, without restriction, and from the time of publication. PLOS allows rare exceptions to address legal and ethical concerns. See the PLOS Data Policy and FAQ for detailed information.	Yes - all data are fully available without restriction

A Data Availability Statement describing where the data can be found is required at submission. Your answers to this question constitute the Data Availability Statement and **will be published in the article**, if accepted.<br><br>**Important:** Stating 'data available on request from the author' is not sufficient. If your data are only available upon request, select 'No' for the first question and explain your exceptional situation in the text box.<br><br>Do the authors confirm that all data underlying the findings described in their manuscript are fully available without restriction?	
**Describe where the data may be found in full sentences. If you are copying our sample text, replace any instances of XXX with the appropriate details.**<br><br>• If the data are **held or will be held in a public repository**, include URLs, accession numbers or DOIs. If this information will only be available after acceptance, indicate this by ticking the box below. For example: *All XXX files are available from the XXX database (accession number(s) XXX, XXX.).*<br>• If the data are all contained **within the manuscript and/or Supporting Information files**, enter the following: *All relevant data are within the manuscript and its Supporting Information files.*<br>• If neither of these applies but you are able to provide **details of access elsewhere**, with or without limitations, please do so. For example:<br><br>*Data cannot be shared publicly because of [XXX]. Data are available from the XXX Institutional Data Access / Ethics Committee (contact via XXX) for researchers who meet the criteria for access to confidential data.*<br><br>*The data underlying the results presented in the study are available from (include the name of the third party*	All Co1 and ITS2 sequences area available from GenBank accession numbers (MT021761-MT021824; MT027551-MT027599; MT038907-MT038996; MT040955-MT040966). These will be made accessible on request.

and contact information or URL).<br>• This text is appropriate if the data are owned by a third party and authors do not have permission to share the data.<br><br>* typeset	
Additional data availability information:	

Manuscript Click here to access/download;Manuscript;Manuscript.docx

Full Title: A diagnostic real-time PCR assay for the rapid identification of the tomato-potato psyllid, *Bactericera cockerelli* (Šulc, 1909) and development of a psyllid barcoding database.

Authors

JC Sumner-Kalkun[1]*, MJ Sjölund[1], YM Arnsdorf[1], M Carnegie[1], F Highet[1], D Ouvrard[2, 5], AFC Greenslade[3], JR Bell[3], R Sigvald[4], DM Kenyon[1]

[1] SASA, Roddinglaw Road, Edinburgh EH12 9FJ, UK. [2] Department of Life Sciences, Natural History Museum, Cromwell Road, London SW7 5BD, UK. [3] Rothamsted Insect Survey, Rothamsted Research, West Common, Harpenden, Hertfordshire, AL5 2JQ, UK. [4] Department of Ecology, Swedish University of Agricultural Sciences, Box 7044 750 07 Uppsala, Sweden. [5]Entomology and invasive plants Unit, Plant Health Laboratory, ANSES, 755 avenue du campus Agropolis, CS 30016, 34988 Montferrier-sur-Lez Cedex, France

*Corresponding author: jason.sumner-kalkun@sasa.gov.scot

Short Title: *Bactericera cockerelli* diagnostic assay

Keywords: *Bactericera cockerelli,* Biosecurity, Phytosanitary, Diagnostic, Pest, Vector, '*Candidatus* Liberibacter solanacearum', real-time PCR

Abstract

The accurate and rapid identification of ~~many~~ insect pests is an important step in the prevention and control of outbreaks in areas that are otherwise pest free. The potato-tomato psyllid *Bactericera cockerelli* (Šulc, 1909) is the main vector of '*Candidatus* Liberibacter solanacearum' on potato and tomato crops in ~~Central and~~ Northern America and New Zealand. This study describes the design and validation of the first species-specific TaqMan probe-based real-time PCR assay, targeting the ITS2 gene region of *B. cockerelli*. The assay ~~successfully~~ detected *B. cockerelli* genomic DNA from adults ~~(100% accuracy, n=72);~~ immatures ~~(100% accuracy, n=26)~~ and eggs ~~(100% accuracy, n=25)~~. This assay also detected DNA from cloned plasmids containing the ITS2 region of *B. cockerelli* (100% accuracy, n=24). The assay showed 0% false positives when tested on genomic and cloned DNA from 73 other psyllid species collected from across Europe, New Zealand and Mexico. This included 8 other species in the *Bactericera* genus and the main vectors of '*Candidatus* Liberibacter solanacearum' worldwide. The limit of detection for this assay at optimum conditions was 0.000001ng DNA (~200 copies) of ITS2 DNA which equates to around a 1:10000 dilution of DNA from one single adult specimen. This assay is the first real-time PCR based method for accurate, robust, sensitive and specific identification of *B. cockerelli* from all life stages. It can be used as a surveillance and monitoring tool to further study this important crop pest and to aid the prevention of outbreaks, or to prevent their spread after establishment in new areas.

1. Introduction

The psyllid *Bactericera cockerelli* (Šulc, 1909), (commonly known as "Potato Psyllid~~s~~" or "Tomato-Potato Psyllid"), is a major pest of cultivated Solanaceous crops including potato

and tomato [1]. Feeding by this psyllid causes severe damage to potato plants including: deformed tubers; production of numerous small, poor quality tubers; curling of leaves and petioles; and yellowing or purpling of leaves. This leads to stunted growth and loss of yield [2]. *Bactericera cockerelli* is also the main vector of '*Candidatus* Liberibacter solanacearum' (Lso) which is associated with Zebra Chip in Central and North America and New Zealand [3–8].

While *B. cockerelli* prefers to complete its life cycle on Solanaceous plants it can also complete development on species of Convolvulaceae (Bindweeds and Morning Glories) [9]. In addition, adult *B. cockerelli* have been found on over 40 species belonging to 20 families, however most of these are either casual, food or shelter plants on which the psyllid is unable to complete a full life cycle [2,10–16]. Four biotypes of *B. cockerelli* have been described according to polymorphisms in the mitochondrial cytochrome *c oxidase subunit I* (COI) gene and represent geographically distinct populations; central, western, north-western, and south-western [17,18]. Evidence suggest that these genetic types may differ in their ability to spread Lso [18,19].

Bactericera cockerelli is thought to originate from South-Western USA and Mexico [2,11] and from here has spread via natural and human-mediated dispersal to extend its range [20]. Outside America it is now established in New Zealand [21] and more recently Western Australia [22].

The phloem-limited bacterium '*Candidatus* Liberibacter solanacearum' (Lso) is a pathogen associated with Zebra Chip disease of potatoes [3,23–25] and disease in other Solanaceous crops such as cultivated tomato [1,3,26,27], pepper [28], eggplant [29], tobacco [30,31] and tomatillo [26]. Currently, *B. cockerelli* is the main vector of Lso in field and glasshouse-grown

Solanaceous plants in the United States, Mexico, areas of Central America [27–30], Canada [32], New Zealand [5,6,25] and recently Ecuador [33]. Ten Lso haplotypes have been described, only three of which are associated with disease in Solanaceous plants.Haplotypes A, B, and F are associated with Zebra chip disease in America [3,34,35], whereas only haplotype A has been found in New Zealand [5,36]. Haplotype B has also been found in *Bactericera maculipennis* (Crawford) [37]. The remaining haplotypes are not vectored by *B. cockerelli* but by closely related species in the Triozidae family.

The impact of *B. cockerelli* and associated Lso transmission on agriculture is significant. Since its arrival in New Zealand circa 2005 via human-mediated dispersal it has caused millions of dollars of economic losses [6,21]. Similarly, management of *B. cockerelli* in the US is reported to have cost millions of dollars per year in major potato growing areas such as Texas [38] and the Pacific ~~North-West~~ [39]. The introduction of *B. cockerelli* into potato growing regions in Europe or Asia would be devastating to the agricultural industry of those regions. If *B. cockerelli,* or a sufficient vector of Solanaceous Lso haplotypes, were to invade Europe it is estimated that the effects of Lso damage on potato and tomato would cost € 222 million per year and the negative impact of social welfare could cost an additional estimated € 114 million [40].

Currently, *B. cockerelli* is considered an A1 quarantine pest in the EPPO region [4]. Consignments of aubergine and *Capsicum* from Mexico infested with immature and adult stages of *B. cockerelli* were intercepted four times during UK border inspections between 2017-2018; indicating that there is a real threat of this pest making an incursion into the EPPO region if not properly monitored [41]. Monitoring and prevention of the spread of *B. cockerelli* is essential to prevent the risk of an outbreak of Lso on potato, tomato and other

Solanaceous crops in areas where it is not currently found [42]. There is therefore an evident need for a rapid and accurate diagnostic test to identify *B. cockerelli* at all life stages not only as a tool to support import inspections, but also to assist monitoring, eradication and control strategies.

We designed a species-specific real-time PCR diagnostic assay to detect all life-stages of *B. cockerelli*, eggs, immatures and adults. The assay provides a rapid diagnostic test to quickly determine the presence of *B. cockerelli*, allowing for the early detection of invasions/introductions and aiding in the prevention of spread of this psyllid.

2. Materials and Methods

2.1. Specimen collection

The assay was tested on 28 target adults *B. cockerelli* specimens and 73 non-target species consisting of 110 specimens see results section 3.1 for more info on samples. The classification follows Burckhardt & Ouvrard [43], and a complete taxonomic account of each species is given in Ouvrard [20]. Psyllid identifications were confirmed against reference type specimens in the NHM London collections. To account for intraspecific genetic variation, we obtained *B. cockerelli* specimens from Mexico (Universidad Autónoma Agraria Antonio Narro) and USA (USDA, Agricultural Research Services) from colony collections of each of the four recognised biotypes of *B. cockerelli* in Central America, the Central, Western, ~~North-Western~~, and S~~outh-Western~~ biotypes [19]. Specimens of *B. cockerelli* were also obtained from New Zealand lab-reared colonies (Plant Research, New Zealand). Non-target specimens were mainly obtained from 12.2 m suction-traps in the United Kingdom

that form part of the Rothamsted Insect Survey network described here [44]. Specimens were also obtained from suction-traps in Finland, Germany, Spain and Sweden; as well as from field collections from Finland, Israel, Mexico, Serbia, Spain, UK and USA. Non-target specimens from different regions ~~of the USA~~ were used to test assay specificity on species that are commonly found in the same region as *B. cockerelli*. As immatures and eggs are the most likely life stages that inspectors might find on imported plant material, we also tested the assay on DNA extracted from immatures and eggs from Mexico and the USA for validation.

2.2. <u>DNA extraction, PCR, and DNA sequencing for identification of psyllids</u>

DNA for sequencing and assay validation was extracted from psyllids using a non-destructive method first described in [45] and adapted from [46]. Psyllid specimens were preserved in 95% Ethanol: 5% Glycerol solution. Using a 15mm long, 0.15mm diameter stainless steel entomological head-less pin (A3 size, Watkins and Doncaster) mounted in a holder, specimens were initially pierced fully through the abdomen and half-way through the thorax from the dorsal side while attempting to minimise damage to head, legs, wings, terminalia and other body parts that are used for taxonomic identification. Pierced specimens were placed in a microcentrifuge tube containing 180 µl of ATL buffer and 20 µl of proteinase-k as outlined in the DNeasy Blood and Tissue Kit from Animal Tissues (Qiagen). Samples were placed in a shaking incubator over-night (~8-10 hrs) at 56 °C at 300 rpm. The protocol for DNA extr[illegible]on in DNeasy Blood and Tissue Kit Protocol from Animal Tissues (Qiagen) was followed and the psyllid integument voucher specimen was stored in 95% Ethanol: 5% Glycerol for morphological identification. Psyllids were DNA barcoded using one or two gene regions. The internal transcribed spacer 2 (ITS2) and cytochrome c oxidase subunit 1 (CO1)

were amplified and sequenced for identification of different psyllid species. For amplification of ITS2 primers CA55p8sFcm-F and CA28sB1d-R [47] were used; and for amplification of CO1 gene regions arthropod barcoding Primers LCO1490 and HCO2198 [48] were used. All reactions were performed in 20 µl consisting of: 10 µl 2x Type-It Microsatellite PCR Kit Master Mix (Qiagen); 0.2 µM each forward and reverse primer; 7.2 µl molecular grade water (Sigma-Aldrich) and 2 µl of psyllid template DNA. Reactions were run on a Veriti 96-well thermal cycler (Applied Biosystems) using the following programs. ITS2: 95°C for 5 mins; 25 x cycles of (95°C for 30 s, 56°C for 90 s, 72°C for 30 s); and a final extension at 72°C for 10 mins. CO1: 94°C for 5 mins; 5 x cycles of (94°C for 30s, 45°C for 30s, 72°C for 1 min); 25 x cycles of (94°C for 30s, 51°C for 1 min, 72°C for 1 min); and a final extension of 72°C for 10 mins. PCR amplified gene regions were cleaned-up using EXO-SAP and Ethanol precipitation, then sequenced using the BigDye Terminator Cycle Sequencing Kit (Applied Biosystems), forward and reverse complimentary DNA strands were sequenced separately for each sample and analysed using a 3500xL Genetic Analyser (Applied Biosystems).

<u>2.3. Bioinformatics and Real-Time PCR Assay Design</u>

Sequence editing, assembly and alignment were performed on ".AB1" trace files uploaded to Geneious R11 v 11.1.5 (Biomatters Ltd.). Contigs were assembled after trimming sections of low-quality sequence and aligning the complimentary strands using CLUSTAL-W multiple sequence alignment method [49]. Final contigs for each species and each gene region were aligned to identify variable areas suitable as targets for *B. cockerelli* specific primer and probe sets. Primers and probes were designed using manual selection of target-specific regions analysed using the "Basic Local Alignment Search Tool" (BLAST) [50] against the

NCBI GenBank database [51] and processing of selected regions for suitability/ specificity in “Primer3” [52] and “Primer-BLAST” software [53]. Primer annealing temperature, hairpin formation, self-complementarity, GC content and were assessed using “Primer3” [52]. Potential amplification of non-specific insect species was checked using Primer BLAST which includes all psyllid species present in the GenBank database. Primer and probe sets were selected/rejected based on the following parameters: primer annealing temperature 59-62°C; primer annealing temperature + 8-10°C for probe annealing temperature; no more than 2°C difference in annealing temperature between primers, max probe length 30bp, no more than 3 Gs in a row in probe, amplicon length max 300bp and specificity to *B. cockerelli.*

2.4. Real-time PCR Set-up and Standards

To calculate standard curves DNA standards of *B. cockerelli* were prepared using dilution series of linearized cloned plasmid DNA. DNA was extracted as above using the non-destructive method, amplified and cloned into competent *Escherichia coli* cells using the TOPO TA cloning kit (Thermo-Fisher). DNA from successfully transformed colonies was extracted using “PureYield Plasmid Miniprep System” (Promega). For assay validation ITS2 DNA was cloned from other psyllid species (see results section 3.1). Stock DNA 10 ng/µl was linearised from cloned plasmid DNA using EcoRI restrictions enzyme (New England Biolabs), 0.5 µl of enzyme was added to 100 µl of stock DNA, this solution was incubated in a heat block (Thermomixer C, Eppendorf) at 37 °C for 15 mins. The enzyme was then deactivated at 65 °C for 20mins. Real-time PCRs were performed in 15 µl volumes including: 6.75 µl Jumpstart Taq Ready Mix (Sigma); 1.2 µl $MgCl_2$ (25mM); 0.45 µl of each primer; 0.15 µl probe; 4 µl of molecular grade water (Sigma); and 2 µl of template DNA. The standard real-time PCR cycle program was as follows. Hold stage: 50 °C for 2 mins then; 95 °C for 10 mins.

PCR stage: 40 cycles of (95 °C for 15 secs; X °C for 1 min), with primer annealing temperature X being 58, 60, 62, 64, or 68; depending on the experiment. Primer concentration, $MgCl_2$ concentration and temperature was adjusted for validation and optimization of the assay as described below. Reactions were performed on a “QuantStudio 6 Flex” (Applied Biosystems) real-time PCR machine and analysis was done on the “QuantStudio Real-Time PCR Software” (Applied Biosystems).

2.5. <u>Assay validation</u>

2.5.1. Specificity

The final primer and probe set was tested on genomic DNA from 47 *B. cockerelli* specimens from different life stages. These included the 4 US biotypes [17,54] and specimens from New Zealand to determine false negatives. The assay was tested for specificity against genomic DNA of 73 non-target psyllid species collected as mentioned above, to detect false positives. This included a total of 8 other closely related *Bactericera* spp. and the major vectors of Lso on Apiaceous crops (*B. nigricornis*, *B. trigonica* and *Trioza apicalis*). Information regarding samples tested is in results section 3.1. The assay was also checked for cross-reaction against potato genomic DNA (*Solanum tuberosum*), 3 samples of *S. tuberosum* ‘Maris Piper’ were tested in replicates of 8. All reactions with non-target DNA were run in conjunction with a TaqMan Exogenous Internal Positive Control Reagent Kit (Applied Biosystems) to rule out the possibility that false positives were not obtained due to inhibition within the reaction. DNA from all non-target psyllids was sequenced in either ITS2, CO1 or both to ensure psyllid DNA was present in all reactions to rule out false negatives due to inefficient DNA extraction. Reactions were performed in duplicate at least, with a higher number of replicates for species closely related to *B. cockerelli.* False positives were

defined as reactions with non-target DNA that showed fluorescence above the cycle threshold during 40 cycles; and false negatives were defined as reactions with *B. cockerelli* DNA that did not give a C_t after 40 cycles.

2.5.2. Sensitivity

Experiments were performed to determine the limit of detection of the assays. DNA standards were produced using *B. cockerelli* linearized cloned DNA from the ITS2 region. A nine point 10-fold dilution series starting with 10 ng/µl DNA up to 10^$^{-8}$ ng/µl of linearised plasmid DNA and genomic DNA was used to determine the limit of detection. 100ng/µl stock DNA concentration was initially checked using QuBit 4 Fluorometer (Invitrogen) and 5 µl was added to 45 µl of molecular grade water (Sigma-Aldrich) to dilute 1:10; eight subsequent dilutions were made. Linearised and non-linearised DNA was compared along with genomic DNA. The ability of the assay to detect immatures and eggs was also tested. DNA from various instars of immatures was extracted using the non-destructive protocol described above. Batches of 1 egg, 5 eggs and 10 eggs were extracted using the DNeasy Blood & Tissue kit (Qiagen) and initially broken with a pestle.

2.5.3. Repeatability and Reproducibility

Variation in the performance of the assay between runs and within runs was assessed at a 0.2 µM primer concentration, with 1.5mM $MgCl_2$, and 60 °C annealing temperature. Linearised plasmid DNA from *Escherichia coli* transformed with *B. cockerelli* ITS2 DNA was used. A six point 1:10 dilution series starting at 10ng/µl was used with each dilution being performed in triplicate. The same experiment was repeated 3x simultaneously. Runs and variations between the three experiments were recorded and analysed using QuantStudio 6

Real-Time PCR Software. An identical plate following the same plate set-up and reaction mix was run simultaneously on another QuantStudio 6 real-time PCR machine to compare inter-run variation.

2.5.4. Robustness/Optimization

Amplification of target DNA, specificity and sensitivity at different $MgCl_2$ concentration, primer concentrations and annealing temperatures were performed to assess robustness. The assay was tested with 1.5, 3.5, 5.5, 7.5 and 9.5mM $MgCl_2$ concentration. For primers, 0.1, 0.2, 0.3, 0.5 and 1.0 µM concentrations were tested. The assay was also tested at different annealing temperatures 58, 60, 62, 64, 68 °C across. For each tested parameter, optimization was performed across a nine point 1:10 dilution series starting at 10ng/µl DNA. All samples were tested in triplicates. Closely related *Bactericera* species were included in these assays to assess specificity under different assay conditions. After optimization of the assay a multifactorial robustness test was performed across two different real-time PCR machines to test the combined effects of small changes/errors in the PCR set-up. The assays were run on a “QuantStudio 6 Flex” (Applied Biosystems) and “CFX96 Real-Time System” (BioRad); results were analysed using “QuantStudio 6 Real-Time PCR Software” (Applied Biosystems) and “CFX Manager 3.1” (BioRad). The methodology used followed the European Network of GMO Laboratories (ENGL) recommendations [55].

3. RESULTS

3.1. DNA extraction, PCR, and DNA sequencing for identification of psyllids

DNA from 110 psyllid specimens comprising 73 different species were extracted, amplified and sequenced successfully from either CO1 or ITS2 gene regions, or both (Tab. 1).

Family	Genus	Species	Voucher ID	Collection Location	Collection method	CO1 Ac#	ITS2 Ac#	Tech Reps	Voucher Location
**Aphalaridae**	*Aphalara*	*avicularis*	160718.A.avi.23	Wellesbourne, UK	suction trap	MT021761	/	2	1
		polygoni	160718.A.pol.22	Wellesbourne, UK	suction trap	/	MT038907	2	1
	Blastopsylla	*occidentalis*	180312.Bl.occ.24	Salamanca, Spain	suction trap	MN272146	MN316692	2	***3***
	Craspedolepta	*gutierreziae*	160825.5 US	Nevada, USA	field collection	MT021786	MT038962	2	1
		minutissima	160825.1 US	Nevada, USA	field collection	MT021787	MT038963	2	1
			160825.10 US	Oregon, USA	field collection	MT021789	/	2	1
			160825.4 US	Nevada, USA	field collection	MT021788	/	2	1
		nervosa	160728.Cra.ner.2	Gogarbank, UK	suction trap	MT021790	MT038964	2	1
		pinicola	160825.2 US	Nevada, USA	field collection	/	MT038965	2	1
		subpunctata	160421.C.sub.5	Gogarbank, UK	suction trap	MT021791	MT038966	2	1
	Rhinocola	*aceris*	151014.R.ace.14	Wellesbourne, UK	suction trap	MT021810	MT038979	2	2
**Liviidae**	*Diaphorina*	*citri*	160309.D.cit.6	Lab Colony,	Lab Reared	MT021794	MT038969	2	1
	Euphyllura	*olivina*	180125.Eup.oli.3	imports from Italy	imported *Olea europeae*	MT021797	MT038970	2	***3***
	Livia	*crefeldensis*	180312.L.cre.5	Salamanca, Spain	suction trap	MN316678	MN272127	2	***3***
		junci	160404.L.jun.1	Broom' s Barn, UK	suction trap	MT021801	/	2	2
		opaqua	160825.6 US	Nevada, USA	field collection	MT021802	MT038973	2	1
**Psyllidae**	*Arytaina*	*genistae*	151203.A.gen.2J	Ayr, UK	suction trap	/	MT038909	2	1
	Arytainilla	*gredi*	180312.A.gre.1	Salamanca, Spain	suction trap	MN272123	MN316677	2	***3***
		spartiophila	180716.A.spa.29	Edinburgh, UK	suction trap	MT021762	MT038908	2	***3***
	Baeopelma	*foersteri*	151203.B.foe.1J	Ayr, UK	suction trap	/	MT038944	2	1
		foersteri	160928.B.foe.2	SASA, UK	suction trap	MT021776	/	2	1
	Cacopsylla	*affinis*	151203.C.aff.1	Wye, UK	suction trap	MT021777	MT038945	2	2
		ambigua	160404.C.amb.4	Wye, UK	suction trap	/	MT038946	2	2
		ambigua	161024.C.amb.3	Preston, UK	suction trap	/	MT038947	2	1
		americana	160825.3 US	Nevada, USA	field collection	MT021778	MT038948	2	1
		brunneipennis	160309.C.bru.8	Wye, UK	suction trap	/	MT038949	2	2
		crataegi	160404.C.cra.3	Broom' s Barn, UK	suction trap	MT021779	MT038950	2	2
		mali	180910.C.mal.30	Elcho, UK	field collection	/	MT038951	2	***3***
		melanoneura	160718.C.mel.6	Kirton, UK	suction trap	/	MT038952	2	***3***
		moscovita	190109.C.mos.1	Germany	suction trap	/	/	2	***3***
		peregrina	161024.C.per.11	Silwood Park, UK	suction trap	MT021780	MT038953	2	1
		pruni	160203.C.pru.18	Wellesbourne, UK	suction trap	/	MT038954	2	2
		pulchra	160718.C.pul.15	Elgin, UK	suction trap	/	MT038955	2	1
		pyricola	160203.C.pco.2	Wye, UK	suction trap	MT021781	MT038956	2	2
		saliceti	161024.C.sal.7	York, UK	suction trap	/	MT038958	2	1
		sorbi	161024.C.sor.8	Preston, UK	suction trap	MT021782	MT038959	2	1
		rhamnicola	151014.C.rha.8	Wellesbourne, UK	suction trap	/	MT038957	2	2
		ulmi	171011.C.ulm.13	Germany	suction trap	MT021783	MT038960	2	***3***
	Ceanothia	*ceanothi*	160825.9 US	Oregon, USA	field collection	MT021784	/	2	1
	Chamaepsylla	*hartigii*	160728.Ch.har.1	Gogarbank, UK	suction trap	MT021785	MT038961	2	1
	Euglyptoneura	*fuscipennis*	160825.7 US	Oregon, USA	field collection	MT021795	/	2	1
		robusta	160825.8 US	Oregon, USA	field collection	MT021796	/	2	1
	Heteropsylla	*texana*	160825.11 US	Texas, USA	field collection	MT021798	/	2	1
	Psylla	*alni*	161019.P.aln.1	Sweden	suction trap	MT021804	/	2	1
		buxi	180622.P.bux.22	Scotland, UK	suction trap	MT021806	MT038976	2	***3***
		betulae	161123.P.bet.20	Jokioinen, Finland	suction trap	MT021805	MT038975	2	***3***
	Psyllopsis	*discrepans*	151002.P.dis.8	Sweden	suction trap	MT021807	/	2	1
		fraxini	180716.P.fri.33	Edinburgh, UK	suction trap	MT021808	MT038977	2	***3***
		fraxinicola	160203.P.fra.6	Wellesbourne, UK	suction trap	MT021809	MT038978	2	2
	Spanioneura	*fonscolombii*	180802.S.fon.29	Edinburgh, UK	field collection	/	MT038980	2	***3***
**Spondyliaspidae**	*Ctenarytaina*	*spatulata*	160404.Ct.spa.6	Wye, UK	suction trap	MT021792	MT038967	2	2
		spatulata	161024.Ct.spa.5	Wye, UK	suction trap	MT021793	MT038968	2	1
**Triozidae**	*Bactericera*	*albiventris*	171214.B.alb.11	Jokioinen, Finland	suction trap	/	MT038910	5	***3***
		curvatinervis	161123.B.cur.42	Jokioinen, Finland	suction trap	/	MT038911	5	***3***
		dorsalis	160803.B.dor.2	Florida, USA	lab colony	MT021763	MT038912	5	***3***
		maculipennis	190604.B.mac.1	Lab Colony, USA	Lab Reared	/	MT038913	2	***3***
			190604.B.mac.2	Lab Colony, USA	Lab Reared	/	MT038914	2	***3***
			190604.B.mac.3	Lab Colony, USA	Lab Reared	/	MT038915	2	***3***
			190604.B.mac.4	Lab Colony, USA	Lab Reared	/	MT038916	2	***3***
			190604.B.mac.5	Lab Colony, USA	Lab Reared	/	MT038917	2	***3***
			190604.B.mac.6	Lab Colony, USA	Lab Reared	/	MT038918	2	***3***
			190604.B.mac.7	Lab Colony, USA	Lab Reared	/	MT038919	2	***3***
		nigricornis	170324.B.nig.18	Spain	field collection	MT021764	MT038920	5	***3***
			170324.B.nig.22	Spain	field collection	MT021765	MT038921	5	***3***
		salicivora	190116.B.sal.1	Elgin, UK	suction trap	/	/	6	***3***
		striola	161123.B.str.9	Jokioinen, Finland	suction trap	/	MT038922		
		tremblayi	170731.B.tre.5	Belgrade, Serbia	field collection	/	MT038923	5	***3***

		190604.B.tre.17	Spain	Lab Colony	/	MT038924	2	3
		190604.B.tre.18	Spain	Lab Colony	/	MT038925	2	3
		190604.B.tre.19	Spain	Lab Colony	/	MT038926	2	3
		190604.B.tre.20	Spain	Lab Colony	/	MT038927	2	3
		190604.B.tre.21	Spain	Lab Colony	/	MT038928	2	3
	trigonica	170629.B.tri.16	Tunisia	field collection	MT021766	MT038929	3	3
		170629.B.tri.17	Tunisia	field collection	/	MT038930	3	3
		170629.B.tri.18	Tunisia	field collection	MT021767	MT038931	3	3
		181010.B.tri.17	Spain	Lab Colony	MT021768	MT038932	2	3
		181010.B.tri.18	Spain	Lab Colony	MT021769	MT038933	2	3
		181010.B.tri.19	Spain	Lab Colony	/	MT038934	2	3
		181010.B.tri.20	Spain	Lab Colony	MT021770	MT038935	2	3
		181010.B.tri.21	Spain	Lab Colony	/	MT038936	2	3
		190604.B.tri.23	Spain	Lab Colony	MT021771	MT038937	2	3
		190604.B.tri.24	Spain	Lab Colony	/	MT038938	2	3
		190604.B.tri.25	Spain	Lab Colony	MT021772	MT038939	2	3
		190604.B.tri.26	Spain	Lab Colony	MT021773	MT038940	2	3
		190604.B.tri.27	Spain	Lab Colony	MT021774	MT038941	2	3
		190604.B.tri.28	Spain	Lab Colony	/	MT038942	2	3
		190604.B.tri.29	Spain	Lab Colony	MT021775	MT038943	2	3
Heterotrioza	*chenopodii*	160203.H.che.11	Kirton, UK	suction trap	/	MT038971	2	2
		160825.12 US	Washington, USA	field collection	MT021799	/	2	1
Lauritrioza	*alacris*	160816.L.ala.2	Spain	suction trap	MT021800	MT038972	2	1
Powellia	*vitreoradiata*	161024.P.vit.10	Kirton, UK	suction trap	MT021803	MT038974	2	1
Trioza	*albifrons*	160825.18.US	Nevada, USA	field collection	MT021811	MT038981	2	1
	anthrisci	150708.T.ant.11	Jokioinen, Finland	field collection	MT021812	/	2	3
	apicalis	161019.T.api.5	Sweden	field collection	MT021813	/	2	3
	buxtoni	170324.T.bux.11	Israel	field collection	MT021814	MT038982	2	3
	centranthi	161024.T.cen.9	Wye, UK	suction trap	MT021815	/	2	1
	cerastii	171214.T.cer.32	Vikki, Finland	suction trap	MT021816	MT038983	2	3
	dispar	160718.T.dis.26	Hellfreda, Sweden	suction trap	MT021817	/	2	1
	erytreae	160808.ICA.19	Spain	Lab Colony	/	MT038984	2	1
	flavipennis	160421.T.fla.3	Sweden	suction trap	MT021818	MT038985	2	1
	galii	160203.T.gal.23	Wellesbourne, UK	suction trap	/	MT038986	2	2
	remota	160718.T.rem.8	Sweden	suction trap	/	MT038987	2	1
		180424.T.rem.1	Dundee, UK	Suction trap	MT021819	MT038988	3	3
		180424.T.rem.6	Dundee, UK	Suction trap	MT021820	MT038989	3	3
		180424.T.rem.16	Dundee, UK	Suction trap	MT021821	MT038990	3	3
		180424.T.rem.18	Dundee, UK	Suction trap	MT021822	MT038991	3	3
		180424.T.rem.19	Dundee, UK	Suction trap	/	MT038992	3	3
		190116.T.rem.7	UK	Suction trap	MT021823	MT038993	3	3
	rhamni	151002.T.rha.13	Sweden	suction trap	MT021824	MT038994	2	1
	tatrensis	160718.T.tat.27	Sweden	suction trap	/	MT038995	2	1
	urticae	160816.T.urt.17	Spain	field collection	/	MT038996	2	1

Table 1: Information on non-target psyllid species and plant specimens tested using the *B. cockerelli* real-time PCR assay Bcoc_JSK2 showing number of technical replicates and false positives. All non-target species gave 0% false positives. GenBank Accession numbers are included for ITS2 and CO1 regions if sequencing was successful. Voucher Location: 1= 1; 2= 2 Research Insect Survey; 3= SASA Hemipteran DNA Database. All DNA samples are stored in the SASA Hemipteran DNA database. “/” = no sequence obtained

3.2. Bioinformatics and Real-Time PCR Assay Design.

While differentiation within both the ITS2 and CO1 gene regions was sufficient to discriminate between psyllid species, the ITS2 gene region was more suitable for TaqMan

assay design for *B. cockerelli*. Similarities between CO1 gene sequences between members of the *Bactericera* genus and *B. cockerelli* were higher than in the ITS2 region (average % similarity = 82.51 ± 0.68 for CO1 and 77.80 ± 4.79 for ITS2) (Tab. 2). The ITS2 region showed larger sections of variability along the gene on which to design primers and probes. Several primer and probe sets passed the selection criteria, but most were unsuitable due to high rate of false positives from closely related *Bactericera* species. The final primer and probe set Bcoc_JSK2 (Tab. 3) targets a 187bp region of the ITS2 gene (Fig. 1).

Species	ITS2 % Similarity	ITS2 bp	ITS2 GC content %	CO1 % Similarity	CO1 bp	CO1 GC content %
B. trigonica	78.96	662	59.3	82.88	509	35.4
B. tremblayi	79.16	665	59.1	82.97	682	33
B. curvatinervis	80.30	655	58	82.23	678	34.7
B. nigricornis	81.16	668	59.3	81.28	521	36.7
B. albiventris	76.67	667	59.2	83.41	663	32.9
B. dorsalis	65.59	560	61.3	82.31	685	32.6
B. maculipennis	80.67	674	61.6	nd	nd	nd
B. salicivora	nd	nd	nd	nd	nd	nd
B. striola	79.91	663	59.1	nd	nd	nd
B.cockerelli	N/A	569	61.0	N/A	595	32.6

Table 2. Closely related Bactericera species tested with Bcoc_JSK2 assay. ITS similarity = % identity to DNA sample 150727.B.coc.02. CO1 similarity = % identity to a consensus sequences of all *B. cockerelli* sequences obtained during this study. CO1 genes showed higher similarity and fewer variable regions compared to ITS2 regions. Highest % similarity to *B. cockerelli* in the ITS2 region was found in *B. nigricornis* (81.16) and to *B. albiventris* in the CO1 region (83.41). The Bcoc_JSK2 assay does not give false positives with any of the species listed here. (nd= not determined due to sequencing failing).

Oligo Name	Function	Sequence 5'-3'	Tm	Length (bp)
Bcoc_JSK2-f	forward primer	GAGGTCTCCTCATCGTGCGT	61	25
Bcoc_JSK2-r	reverse primer	GGACGAGCATTGCTGCTGC	62.2	23
Bcoc_JSK2-p	probe (FAM-BHQ)	GCAAACGCGGCACAAGTACCGCGC	70.9	25

Table 3. Final oligonucleotide sequences for the Bcoc_JSK2 TaqMan real-time PCR assay to identify *B. cockerelli*. The assay targets a 187 bp region of the ITS2 gene region.

Sample name	Life Stage	Origin	C_t ave	Tech reps	CO1 Ac#	ITS2 Ac#	DNA Source
181119.B.coc.06	1 egg	Mexico	29.80	2	/	MT027568	Genomic
191003.B.coc.01	1 egg	Mexico	33.41	3	/	MT027592	Genomic
191003.B.coc.02	1 egg	Mexico	24.95	3	/	MT027593	Genomic
191003.B.coc.03	1 egg	Mexico	33.79	3	/	MT027594	Genomic
191003.B.coc.04	1 egg	Mexico	22.43	6	/	MT027595	Genomic
181119.B.coc.07	5 eggs	Mexico	24.42	2	/	MT027569	Genomic
181119.B.coc.21	5 eggs	Mexico	28.32	2	/	MT027582	Genomic
181119.B.coc.08	10 eggs	Mexico	29.61	2	/	MT027570	Genomic
181119.B.coc.22	10 eggs	Mexico	26.43	2	/	MT027583	Genomic
181119.B.coc.03	immature	Mexico	22.56	2	/	MT027565	Genomic
181119.B.coc.04	immature	Mexico	22.33	2	/	MT027566	Genomic
181119.B.coc.05	immature	Mexico	21.46	2	/	MT027567	Genomic
181119.B.coc.11	immature	Mexico	23.16	2	/	MT027573	Genomic
181119.B.coc.12	immature	Mexico	24.15	2	/	MT027574	Genomic
181119.B.coc.13	immature	Mexico	23.94	2	/	MT027575	Genomic
181119.B.coc.14	immature	Mexico	25.75	2	/	MT027576	Genomic
181119.B.coc.16	immature	Mexico	23.49	2	/	MT027578	Genomic
181119.B.coc.18	immature	Mexico	22.45	2	/	MT027580	Genomic
181119.B.coc.19	immature	Mexico	23.50	2	/	MT027581	Genomic
190604.B.coc.13	immature	Mexico	24.96	2	/	MT027588	Genomic
190604.B.coc.14	immature	Mexico	25.09	2	/	MT027589	Genomic
190604.B.coc.15	immature	Mexico	28.37	2	/	MT027590	Genomic
150727.B.coc.02	Adult	South Western, USA	22.18	2	MT040955	MG719775	Genomic
150827.B.coc.02	Adult	South Western, USA	22.18	2	MT040956	MT027597	Genomic
150827.B.coc.03	Adult	Central USA	24.49	6	MT040957	MT027598	Genomic
150827.B.coc.04	Adult	North Western, USA	24.77	2	MT040958	MT027599	Genomic
150827.B.coc.06	Adult	North Western, USA	23.68	2	MT040960	MT027552	Genomic
150827.B.coc.12	Adult	Western, USA	20.39	2	MT040961	MT027596	Genomic
150827.B.coc.17	Adult	South Western, USA	19.65	2	MT040962	MT027553	Genomic
160725.B.coc.05	Adult	Central, USA	21.45	2	MT040963	/	Genomic
160726.B.coc.01	Adult	New Zealand	21.56	2	/	MT027557	Genomic
160726.B.coc.02	Adult	New Zealand	21.02	2	/	MT027558	Genomic
160726.B.coc.03	Adult	New Zealand	20.48	2	/	MT027559	Genomic
160726.B.coc.04	Adult	New Zealand	21.98	2	/	MT027560	Genomic
160726.B.coc.05	Adult	New Zealand	19.43	2	/	MT027561	Genomic
160726.B.coc.06	Adult	New Zealand	20.96	2	/	MT027562	Genomic
180731.B.coc.04	Adult	North Western, USA	24.42	6	MT040964	/	Genomic
180731.B.coc.05	Adult	Western, USA	22.91	6	MT040965	/	Genomic
180731.B.coc.06	Adult	Western, USA	27.14	6	MT040966	/	Genomic
181119.B.coc.01	Adult	Mexico	21.47	2	/	MT027563	Genomic
181119.B.coc.02	Adult	Mexico	19.98	2	/	MT027564	Genomic
181119.B.coc.09	Adult	Mexico	21.83	2	/	MT027571	Genomic
181119.B.coc.10	Adult	Mexico	19.48	2	/	MT027572	Genomic

181119.B.coc.15	Adult	Mexico	21.27	2	/	MT027577	Genomic
181119.B.coc.17	Adult	Mexico	23.74	2	/	MT027579	Genomic
190604.B.coc.09	Adult	USDA, Lab Colony	21.51	2	/	MT027584	Genomic
190604.B.coc.10	Adult	Mexico	20.33	2	/	MT027585	Genomic
190604.B.coc.11	Adult	Mexico	22.67	2	/	MT027586	Genomic
190604.B.coc.12	Adult	Mexico	24.37	2	/	MT027587	Genomic
190604.B.coc.16	Adult	Mexico	27.15	2	/	MT027591	Genomic
150827.B.coc.05.col.04	transformed *E. coli*	Lab	11.23	6	MT040959	MT027551	Cloned, 10ng
160725.B.coc.01.col.06	transformed *E. coli*	Lab	11.55	6	/	MT027554	Cloned, 10ng
160725.B.coc.06.col.04	transformed *E. coli*	Lab	11.78	6	/	MT027555	Cloned, 10ng
160725.B.coc.07.col.08	transformed *E. coli*	Lab	11.67	6	/	MT027556	Cloned, 10ng

Table 4: Information on *Bactericera cockerelli* samples tested with Bcoc_JSK2 assay including genomic DNA from adults, immatures, single eggs and egg batches. Location of samples collection is also included. All samples gave 100% positives. Accession numbers for CO1 and ITS2 (MT027551-MT027599) regions are included. "/" = no sequence obtained.

Figure 1. CLUSTAL-W alignment of ITS2 regions from closely related *Bactericera* species showing variable regions and the gene target for the Bcoc_JSK2 primer and probe set. Bases shades with black show differences to *B. cockerelli* sequence. Colour highlights locations of forward primer (blue highlight); reverse primer (green highlight) and probe (yellow highlight). The probe and reverse primer are reverse compliments of the highlighted regions here.

3.3. Specificity and Sensitivity

This assay did not amplify DNA from any of the 73 non-target psyllid species or *Solanum tuberosum* DNA when tested at 60 °C with primer concentration 0.2 µM. Samples included nine closely related *Bactericera* species with similar ITS2 and CO1 sequences (Tab. 2). Under optimal conditions, false negatives = 0% for all non-target species tested with pure genomic DNA, giving a diagnostic specificity of 100%. Some suboptimal reaction conditions showed 33% false positives against high concentrations (10 ng / 1 ng) of *Bactericera albiventris* cloned DNA (see below). All *B. cockerelli* genomic DNA samples gave positive results (Tab. 4) giving 0% false negatives across 54 biological replicates and 147 technical replicates;

resulting in a diagnostic sensitivity of 100%. These included *B. cockerelli* specimens from each of the four US biotypes as well as specimens from New Zealand. These specimens included adults, immature stages and eggs. The assay can amplify *B. cockerelli* DNA from both cloned and genomic samples. Under optimal conditions for PCR efficiency and specificity (60 °C, 0.2 µM primer, 1.5 mM $MgCl_2$) the limit of detection was 0.000001 ng DNA across a range of different reaction parameters this equates to 200 copy numbers of ITS2 calculated using the following equation: Number of Copies = (ng DNA x 6.022×10^{23}) ÷ (length of plasmid (4656) + cloned fragment (700)bp) * 1×10^{9} * 660). The copy number calculator available at http://scienceprimer.com/copy-number-calculator-for-realtime-pcr was used. Diagnostic sensitivity was 100% on all DNA extracted from *B. cockerelli* immatures. False negatives from DNA from egg extractions were 0% for single eggs and 0% for batches of 3 and 10 eggs.

3.4. <u>Repeatability and Reproducibility</u>

No significant differences were found between C_t means across the different replicates at different concentrations as tested by two-way ANOVA ($F_{5, 25}$= 0.54, p = 0.955). The assay also performed consistently across different machines and there was no significant difference between runs across the two machines as tested by two-way ANOVA ($F_{1, 5}$= 1.28, p = 0.279).

3.5. <u>Robustness/Optimization</u>

The assays amplified *B. cockerelli* DNA at all primer concentrations, $MgCl_2$ concentrations and annealing temperatures with varying levels of efficiency, precision, and sensitivity (Supp Tabs. S1-S3). At primer concentration 0.5 µM, the assay was less sensitive only amplifying

down to 0.0001 ng DNA. At higher primer concentrations (1.0 µM,) the assay showed higher sensitivity, but efficiency was outside the range for acceptable use. The assay performed optimally at 0.2 µM primer concentration showing good efficiency and high sensitivity (0.000001 ng DNA) (Supp Tab. S1). Generally, standard deviation of the C_t was lower at higher DNA concentrations and some of the primer concentrations showed SD slightly above the accepted level for quantitative real-time PCR, however this module is intended for qualitative use. At high DNA concentrations all primer concentrations are suitable for use with Bcoc_JSK2 primer and probe set to detect *B. cockerelli* but 0.2 µM is recommended for best results. The assay did not amplify non-target DNA from the 8 other *Bactericera* species tested at the different primer concentrations (0.1, 0.2, 0.3, 0.5 and 1.0 µM).

The MgCl2 concentration of the assay made only small differences to the overall performance of the assay (Supp Tab. S2) and the assay was able to amplify *B. cockerelli* DNA at low concentrations (0.000001 ng) at each $MgCl_2$ concentration. The precision of the assay was lower at higher $MgCl_2$ concentrations 7.5mM and 9.5mM (Supp Tab. S2).

Sensitivity was slightly higher at 64 °C giving 33.33% (n=3) positives for only 20 copies of *B. cockerelli* DNA (0.0000001 ng), however at 64 °C and 66 °C 33.33% (n=3) false positives were found with 10ng and 1 ng of *B. albiventris* cloned DNA (Supp Tab. S3). Reactions at 58 °C were 10 to 100-fold less sensitive than reactions at 64 °C. For best sensitivity and specificity, it is suggested that assays using the Bcoc_JSK2 primer and probe set should be performed at 60 °C or 62 °C. While higher temperatures appear to be more sensitive, they are not recommended on unknown samples due to the small likelihood of returning false positives with *B. albiventris* and possibly other un-tested *Bactericera* spp.

It is recommended that this assay be performed at 60 °C – 62 °C, with a $MgCl_2$ concentration of 1.5mM and a primer concentration of 0.2 µM. To test the robustness of these conditions a multifactorial approach was taken [55]. The assay performed satisfactorily across the different treatments and was shown to be robust and unaffected by small changes in assay set-up (Supp Tab. S4). Each treatment gave 100% positives for amplification of *B. cockerelli* genomic DNA.

4. <u>Discussion</u>

The Tomato-Potato psyllid is an economically damaging pest of solanaceous plants that has spread by human mediated dispersal. It causes feeding damage to plants but also is the major vector of '*Candidatus* Liberibacter solanacearum' (Lso), a phloem limited bacterium that is associated with disease in solanaceous and apiaceous plants. Management of this insect pest requires accurate identification of *B. cockerelli*, this is often difficult if eggs or immature life stages only are available for identification. Hitherto, identification of *B. cockerelli* required either considerable expertise in psyllid taxonomy or the lengthy process of DNA barcoding [54].

We have designed and validated the first species-specific, quantitative real-time PCR TaqMan assay for *B. cockerelli* by using the comparison of 73 non-target species to identify unique gene regions that were suitable for primer/probe design and species differentiation. The genus *Bactericera* currently contains 160 species [20] and <1% of these have been tested in the current study due to the difficulty in obtaining other specimens from the field or lab colonies. However Europe is home to 26 different species of *Bactericera* [20], 30% of which have been tested for false positives using this assay. Psyllid species that were tested

are most commonly found in potato and carrot fields in Europe and the wider EPPO region which should minimize the potential for false positives and ensure the assay is efficient at detecting outbreaks in European fields. The assay was also tested on nine closely related *Bactericera* species. The number of species used in our study is relatively high compared to other reported TaqMan assays for plant pests that report lower numbers of non-target species [56,57].

The assay is based on a 187 bp region of the ITS2 gene which was suitable as it contained high interspecific variation consisting of stretches of insertions and deletions (INDELs). The ITS2 region has been used to distinguish species phylogenetically and to identify cryptic species in the *Cacopsylla pruni* complex [47]. DNA sequences obtained from this study will improve psyllid representation on online DNA databases, reducing the chance of Type II errors (i.e. misidentification due to lack of conspecific references) [58]. The *B. cockerelli* sequences on which we tested this assay (and many of the non-target psyllid species) were from different geographic locations to account for intraspecific variation. *Bactericera cockerelli* specimens from the four USA biotypes and specimens from New Zealand all gave 100% true positives.

The success rates of eradications are dependent on the length of time between introduction, detection, and implementation of eradication measures as Lso displays a short transmission time from *B. cockerelli* to potatoes [4,25]. Feasibly, methodology described in this study could be used to extract DNA from a specimen and test for *B. cockerelli* positives within 6-12 hrs or quicker. This is faster than identification by DNA barcoding and could aid in eradications/ prevention of incursions. This time could be reduced further if the real-time assay is used in conjunction with faster DNA extraction protocols.

There are currently no methods described within the EPPO “agreed diagnostic protocol for identification of *B. cockerelli”* [4]. In addition, the current EPPO control system for *B. cockerelli* and Lso [4] highlights the importance of identifying psyllid eggs and immatures on various plant materials during inspections and monitoring but gives minimal guidelines for achieving this. Validation of this assay demonstrates that it would be a reliable and accurate tool for use in this area and it will therefore be prepared for consideration by the EPPO diagnostic panel. This assay is also useful for monitoring *B. cockerelli* occurrence at several spatial scales, from local border checks to regional surveys which use different trapping methods (water, sticky, suction, aerial balloon traps) where no host plant data is available. Given the sensitivity of this assay it should be possible to detect *B. cockerelli* DNA from insect fragments (e.g. legs, heads) if DNA extraction is adequate. However, further validation should be performed to ensure the assay performs adequately on samples obtained from different traps. This assay should be tested on additional congeneric species and other closely related Triozidae psyllids. Another limitation of this assay is that it cannot yet be taken out into the field, making it less portable than LAMP assays or other NGS sequencing techniques such as Nanopore technology.

In conclusion a rapid, specific, robust, repeatable and reliable real-time PCR assay has now been validated and can be used to detect the important pest *B. cockerelli*. This will be an important tool for providing much-needed support to prevent new outbreaks. The assay can be implemented by practitioners with molecular biology experience and does not require personnel to have classical taxonomic knowledge of insects or psyllids; making this tool more accessible than traditional methods. The assay can be used to complement field surveillance and may facilitate further ecological studies of *B. cockerelli* requiring the

identification of immatures and eggs. The strength of this assay lies in the collaboration of molecular biologists and classical taxonomists working together to build a reliable database for DNA barcoding of psyllids.

6. Funding

This work was supported by the EU Horizon2020 Programme under grant agreement No. 635646, POnTE (Pest Organisms Threatening Europe) and the Scottish Government [RRL/001/14]. The Rothamsted Insect Survey, a National Capability, is funded by the Biotechnology and Biological Sciences Research Council under the Core Capability Grant BBS/E/C/000J0200. The authors declare there is no conflict of interest regarding the publishing of this article.

7. Acknowledgements

We thank A. Fereres & C. A. Antolínez Delgado (Institute of Agricultural Sciences, CSIC, Spain), A. Nissinen (Natural Resources Institute Finland), J. Munyaneza, R. Cooper, M. Heidt, K. Swisher Grimm (USDA Agricultural Research Services), S. Bulman (Plant and Food Research, New Zealand), A. Jensen, S. Halbert (Florida Department of Agriculture & Consumer Services, Dept. of Plant Industry) and Alberto Flores (Universidad Autónoma Agraria Antonio Narro) for specimens; and thank C. Jeffries, L. Webster, V. Mulholland, and A. Reid (SASA) for providing advice. We also thank SASA Potato Genotyping team for providing Potato DNA.

8. Author Contributions

JS-K: assay design and validation, investigation, performed analysis, collected data, bioinformatics, visualization, writing- original draft, supervision, project administration; **MJS:** assay design and validation, investigation, performed analysis, collected data, bioinformatics, visualization, writing- original draft, supervision, project administration; **YA:** investigation, performed analysis, validation, data curation, collected data; **MC:** provided resources, data analysis, curation of data, investigation, taxonomy expertise; **FH:** conceived the project, methodology, supervision, writing- original draft; **DO:** provided resources, data analysis, curation of data, taxonomy expertise, writing – review and edit; **AG:** provided resources, data analysis, taxonomy expertise, writing- review and edit; **JB:** conceived the project, provided resources, methodology, writing- review and edit, supervision, project administration; **RS:** provided resources, data analysis, methodology; **DK:** conceived the project, conceptualization, funding acquisition, methodology, supervision, project administration.

9. References

1. Munyaneza JE, Sengoda VG, Crosslin JM, Garzon-Tiznado JA, Cardenas-Valenzuela OG. First report of "*Candidatus* Liberibacter solanacearum" in tomato plants in Mexico. Plant Dis. 2009;93: 1076. doi:10.1094/PDIS-93-10-1076A

2. Wallis RL. Ecological studies on the Potato Psyllid as a pest of potatoes. USDA Tech Bull. 1955;1107: 25.

3. Hansen AK, Trumble JT, Stouthamer R, Paine TD. A new huanglongbing species, "*Candidatus* Liberibacter psyllaurous," found to infect tomato and potato, is vectored by the psyllid *Bactericera cockerelli* (Sulc). Appl Environ Microbiol. 2008;74: 5862–5865. doi:10.1128/AEM.01268-08

4. EPPO. PM 9/25 (1) *Bactericera cockerelli* and '*Candidatus* Liberibacter solanacearum.' EPPO Bull. 2017;47: 513–523. doi:10.1111/epp.12442

5. Liefting LW, Perez-Egusquiza ZC, Clover GRG, Anderson JAD. A new '*Candidatus* Liberibacter' species in *Solanum tuberosum* in New Zealand. Plant Dis. 2008;92: 1474–1474. doi:10.1094/pdis-92-10-1474a

6. Liefting LW, Sutherland PW, Ward LI, Paice KL, Weir BS, Clover GRG. A new '*Candidatus* Liberibacter' species associated with diseases of solanaceous crops. Plant Dis. 2009;93: 208–214. doi:10.1094/PDIS-93-3-0208

7. Munyaneza JE. Zebra Chip disease of potato: Biology, epidemiology, and management. Am J Potato Res. 2012;89: 329–350. doi:10.1007/s12230-012-9262-3

8. Crosslin JM, Hamm PB, Eggers JE, Rondon SI, Sengoda VG, Munyaneza JE. First report of Zebra Chip disease and "*Candidatus* Liberibacter solanacearum" on potatoes in Oregon and Washington State. Plant Dis. 2012;96: 452. doi:10.1094/PDIS-10-11-0894

9. Kaur N, Cooper WR, Duringer JM, Badillo-Vargas IE, Esparza-Díaz G, Rashed A, et al. Survival and development of Potato Psyllid (Hemiptera: Triozidae) on Convolvulaceae: Effects of a plant-fungus symbiosis (Periglandula). PLoS One. 2018;13: 1–19. doi:10.1371/journal.pone.0201506

10. Knowlton G, Thomas W. Host plants of the Potato Psyllid. J Econ Entomol. 1934;27: 547.

11. Pletsch D. The Potato Psyllid *Paratrioza cockerelli* (Sulc), its biology and control. Bull Mont Agric Exp Stn. 1947;446: 1–95.

12. Wallis RL. Potato Psyllid selection of host plants. J Econ Entomol. 1951;44: 815–817. doi:10.1093/jee/44.5.815

13. Butler CD, Trumble JT. The Potato Psyllid, *Bactericera cockerelli* (Sulc) (Hemiptera: Triozidae): life history, relationship to plant diseases, and management strategies. Terr Arthropod Rev. 2012;5: 87–111. doi:10.1163/187498312X634266

14. Prager SM, Esquivel I, Trumble JT. Factors influencing host plant choice and larval performance in *Bactericera cockerelli*. PLoS One. 2014;9. doi:10.1371/journal.pone.0094047

15. Martin NA. Host plants of the Potato / Tomato Psyllid : A cautionary tale. Weta. 2008;35: 12–16.

16. Cooper WR, Horton DR, Miliczky E, Wohleb CH, Waters TD. The weed link in Zebra Chip epidemiology: suitability of non-crop Solanaceae and Convolvulaceae to Potato Psyllid and “*Candidatus* Liberibacter solanacearum.” Am J Potato Res. 2019; 262–271. doi:10.1007/s12230-019-09712-z

17. Swisher KD, Henne DC, Crosslin JM. Identification of a fourth haplotype of *Bactericera cockerelli* (Hemiptera: Triozidae) in the United States. J Insect Sci. 2014;14: 1–7. doi:10.1093/jisesa/ieu023

18. Swisher KD, Munyaneza JE, Crosslin JM. High resolution melting analysis of the Cytochrome Oxidase I gene identifies three haplotypes of the Potato Psyllid in the United States. Environ Entomol. 2012;41: 1019–1028. doi:10.1603/EN12066

19. Swisher KD, Sengoda VG, Dixon J, Echegaray E, Murphy AF, Rondon SI, et al. Haplotypes of the potato psyllid, *Bactericera cockerelli*, on the wild host plant, *Solanum dulcamara*, in the pacific Northwestern United States. Am J Potato Res. 2013;90: 570–577. doi:10.1007/s12230-013-9330-3

20. Ouvrard D. 2018 Psyl'list - The World Psylloidea Database. 2019. Available: http://www.hemiptera-databases.com/psyllist

21. Teulon DA., Workman PJ, Thomas KL, Nielsen MC. *Bactericera cockerelli*: Incursion, dispersal and current distribution on vegetable crops in New Zealand. New Zeal Plant Prot. 2009;62: 136–144.

22. Australian Government Department of Agriculture and Water Resources. Detection of *Bactericera cockerelli* (Tomato-potato psyllid) in Western Australia. Canberra; 2017. Available: https://www.ippc.int/en/countries/australia/pestreports/2017/02/detection-of-bactericera-cockerelli-tomato-potato-psyllid-in-western-australia/

23. Munyaneza JE, Crosslin JM, Upton JE. Association of *Bactericera cockerelli* (Homoptera: Psyllidae) with "Zebra Chip" a New Potato Disease in Southwestern United States and Mexico. J Econ Entomol. 2007;100: 656–663. doi:10.1603/0022-0493(2007)100[656:AOBCHP]2.0.CO;2

24. Munyaneza JE, Fisher TW, Sengoda VG, Garczynski SF, Nissinen A, Lemmetty A. Association of "*Candidatus* Liberibacter solanacearum" with the psyllid, *Trioza apicalis* (Hemiptera: Triozidae) in Europe. J Econ Entomol. 2010;103: 1060–1070. doi:10.1603/EC10027

25. Munyaneza JE, Sengoda VG, Stegmark R, Arvidsson AK, Anderbrant O, Yuvaraj JK, et al. First report of "*Candidatus* Liberibacter solanacearum" associated with psyllid-affected carrots in Sweden. Plant Dis. 2012;96: 453–453. doi:10.1094/PDIS-10-11-0871

26. Liefting LW, Weir BS, Pennycook SR, Clover GRG. "*Candidatus* Liberibacter solanacearum", associated with plants in the family Solanaceae. Int J Syst Evol Microbiol. 2009;59: 2274–2276. doi:10.1099/ijs.0.007377-0

27. Bextine B, Aguilar E, Rueda A, Caceres O, Sengoda VG, McCue KF, et al. First report of "*Candidatus* Liberibacter solanacearum" on tomato in El Salvador. Plant Dis. 2013;97: 1245. doi:10.1094/PDIS-03-13-0248-PDN

28. Munyaneza JE, Sengoda VG, Aguilar E, Bextine B, McCue KF. First report of "*Candidatus* Liberibacter solanacearum" on pepper in Honduras. Plant Dis. 2014;98: 154. doi:10.1094/PDIS-06-13-0598-PDN

29. Munyaneza JE, Sengoda VG, Aguilar E, Bextine BR, McCue KF. First report of "*Candidatus* Liberibacter solanacearum" infecting eggplant in Honduras. Plant Dis. 2013;97: 1654. doi:10.1094/PDIS-06-13-0641-PDN

30. Aguilar E, Sengoda VG, Bextine B, McCue KF, Munyaneza JE. First report of "*Candidatus* Liberibacter solanacearum" on tobacco in Honduras. Plant Dis. 2013;97: 1376. doi:10.1094/PDIS-04-13-0453-PDN

31. Munyaneza JE, Sengoda VG, Aguilar E, Bextine B, McCue KF. First report of "*Candidatus* Liberibacter solanacearum" associated with psyllid-infested tobacco in Nicaragua. Plant Dis. 2013;97: 1244. doi:10.1094/PDIS-03-13-0247-PDN

32. Johnson D. Canadian Potato Psyllid and Zebra Chip Monitoring Network Report. News Lett Can Potato Psyllid Zebra Chip Monit Netw. 2017; 1–19. doi:10.15713/ins.mmj.3

33. Castillo Carrillo C, Fu Z, Burckhardt D. First record of the Tomato Potato Psyllid

Bactericera cockerelli from South America. Bull Insectology. 2019;72: 85–91.

34. Swisher Grimm KD, Garczynski SF. Identification of a new haplotype of '*Candidatus* Liberibacter solanacearum' in *Solanum tuberosum*. Plant Dis. 2019;103: 468–474. doi:10.1094/PDIS-06-18-0937-RE

35. Nelson WR, Fisher TW, Munyaneza JE. Haplotypes of "*Candidatus* Liberibacter solanacearum" suggest long-standing separation. Eur J Plant Pathol. 2011;130: 5–12. doi:10.1007/s10658-010-9737-3

36. Wen A, Mallik I, Alvarado VY, Pasche JS, Wang X, Li W, et al. Detection, distribution, and genetic variability of '*Candidatus* Liberibacter' species associated with zebra complex disease of potato in North America. Plant Dis. 2009;93: 1102–1115. doi:10.1094/pdis-93-11-1102

37. Borges KM, Cooper WR, Garczynski SF, Thinakaran J, Jensen AS, Horton DR, et al. "*Candidatus* Liberibacter solanacearum" associated with the psyllid, *Bactericera maculipennis* (Hemiptera: Triozidae). Environ Entomol. 2017;46: 210–216. doi:10.1093/ee/nvw174

38. CNAS. Economic Impacts of Zebra Chip on the Texas Potato Industry. Texas; 2006.

39. Greenway G, Rondon SI. Economic impacts of Zebra Chip in Idaho, Oregon, and Washington. Am J Potato Res. 2018;95: 362–367.

40. Soliman T. Economic impact assessment of invasive plant pests in the European Union. Wageningen University, Wageningen, Netherlands. 2012.

41. DEFRA. Pest Alert :*Bactericera cockerelli*. 2017. Available:

https://planthealthportal.defra.gov.uk/ph-api/pests/27077/notices/6682/documents/4183/document

42. EPPO. *Bactericera cockerelli*. EPPO Bull. 2013;43: 202–208. doi:10.1111/epp.12044

43. Burckhardt D, Ouvrard D. A revised classification of the jumping plant-lice (Hemiptera: Psylloidea). Zootaxa. 2012;34: 1–34.

44. Bell JR, Alderson L, Izera D, Kruger T, Parker S, Pickup J, et al. Long-term phenological trends, species accumulation rates, aphid traits and climate: Five decades of change in migrating aphids. J Anim Ecol. 2015;84: 21–34. doi:10.1111/1365-2656.12282

45. Sjolund MJ, Ouvrard D, Kenyon D, Highet F. Developing an RT-PCR assay for the identification of psyllid species. Proc Crop Prot North Britain. 2016; 279–282.

46. Percy DM. Radiation, diversity, and host-plant interactions among island and continental legume-feeding psyllids. Evolution (N Y). 2003;57: 2540–2556. doi:10.1111/j.0014-3820.2003.tb01498.x

47. Peccoud J, Labonne G, Sauvion N. Molecular test to assign individuals within the *Cacopsylla pruni* complex. PLoS One. 2013;8: 1–8. doi:10.1371/journal.pone.0072454

48. EPPO. PM 7/129 (1) DNA barcoding as an identification tool for a number of regulated pests. EPPO Bull. 2016;46: 501–537. doi:10.1111/epp.12344

49. Thompson JD, Higgins DG, Gibson TJ. CLUSTAL W: Improving the sensitivity of progressive multiple sequence alignment through sequence weighting, position-specific gap penalties and weight matrix choice. Nucleic Acids Res. 1994;22: 4673–4680. doi:10.1093/nar/22.22.4673

50. Altschul SF, Gish W, Miller W, Myers EW, Lipman DJ. Basic local alignment search tool. J Mol Biol. 1990;215: 403–410. doi:https://doi.org/10.1016/S0022-2836(05)80360-2

51. Clark K, Karsch-Mizrachi I, Lipman DJ, Ostell J, Sayers EW. GenBank. Nucleic Acids Res. 2015/11/20. 2016;44: D67–D72. doi:10.1093/nar/gkv1276

52. Untergasser A, Cutcutache I, Koressaar T, Ye J, Faircloth B, Remm M, et al. Primer3--new capabilities and interfaces. Nucleic Acids Res. 2012;40: e115. Available: http://www.ncbi.nlm.nih.gov/pmc/articles/PMC3424584/

53. Ye J, Coulouris G, Zaretskaya I, Cutcutache I, Rozen S, Madden T. Primer-BLAST: A tool to design target-specific primers for polymerase chain reaction. BMC Bioinfomatics. 2012;13: 134.

54. Liu D, Trumble JT, Stouthamer R. Genetic differentiation between eastern populations and recent introductions of Potato Psyllid (*Bactericera cockerelli*) into western North America. Entomol Exp Appl. 2006;118: 177–183. doi:10.1111/j.1570-7458.2006.00383.x

55. European Network of GMO Laboratories. Definition of minimum performance requirements for analytical methods of GMO testing. 2015. doi:10.2788/65827

56. Li D, Fan Q-H, Waite DW, Gunawardana D, George S, Kumarasinghe L. Development and validation of a Real-Time PCR assay for rapid detection of Two-Spotted Spider Mite, *Tetranychus urticae* (Acari: Tetranychidae). PLoS One. 2015;10: e0131887. doi:10.1371/journal.pone.0131887

57. Dhami MK, Dsouza M, Waite DW, Anderson D, Li D. Real-Time PCR assay for the

identification of the Brown Marmorated Stink Bug (*Halyomorpha halys*). Front Mol Biosci. 2016;3: 1–6. doi:10.3389/fmolb.2016.00005

58. Virgilio M, Jordaens K, Breman FC, Backeljau T, de Meyer M. Identifying insects with incomplete DNA barcode libraries, African fruit flies (Diptera: Tephritidae) as a test case. PLoS One. 2012;7. doi:10.1371/journal.pone.0031581

10. Supporting Information Captions

Supplementary Table 1. Assay performance across a range of primer concentrations at 60 °C and 1.5mM $MgCl_2$. Optimum primer concentration was 0.2 µM showing the best combination of r^2, slope, efficiency, and sensitivity.

Supplementary Table 2. Performance of *B. cockerelli* real-time PCR assay at different magnesium chloride ($MgCl_2$) concentrations.

Supplementary Table 3. Summary of standard curves from optimisation of temperature on Bcoc_JSK2 real-time PCR assay for identification of B. cockerelli. All DNA concentrations tested above the limit of detection (10ng, 1 ng, 0.1ng, 0.01ng 0.001ng, 0.0001ng, 0.00001ng, 0.000001ng) gave 100% positives across 3 x replicates. LOD is given for each temperature. All non-target Bactericera species tested at different DNA concentration gave 0% false positives except for B. albiventris cloned DNA which cross reacted at 64 and 66 °C. (*reactions at 64 °C gave 33.33% positives at 20 copy numbers).

Supplementary Table 4. Set-up and results of multifactorial robustness experiment testing the Bcoc_JSK2 assay on *B. cockerelli* genomic DNA. All treatments showed 100% positives despite small changes to the overall set-up.

Figure 1

Click here to access/download;Figure;Fig1.tif

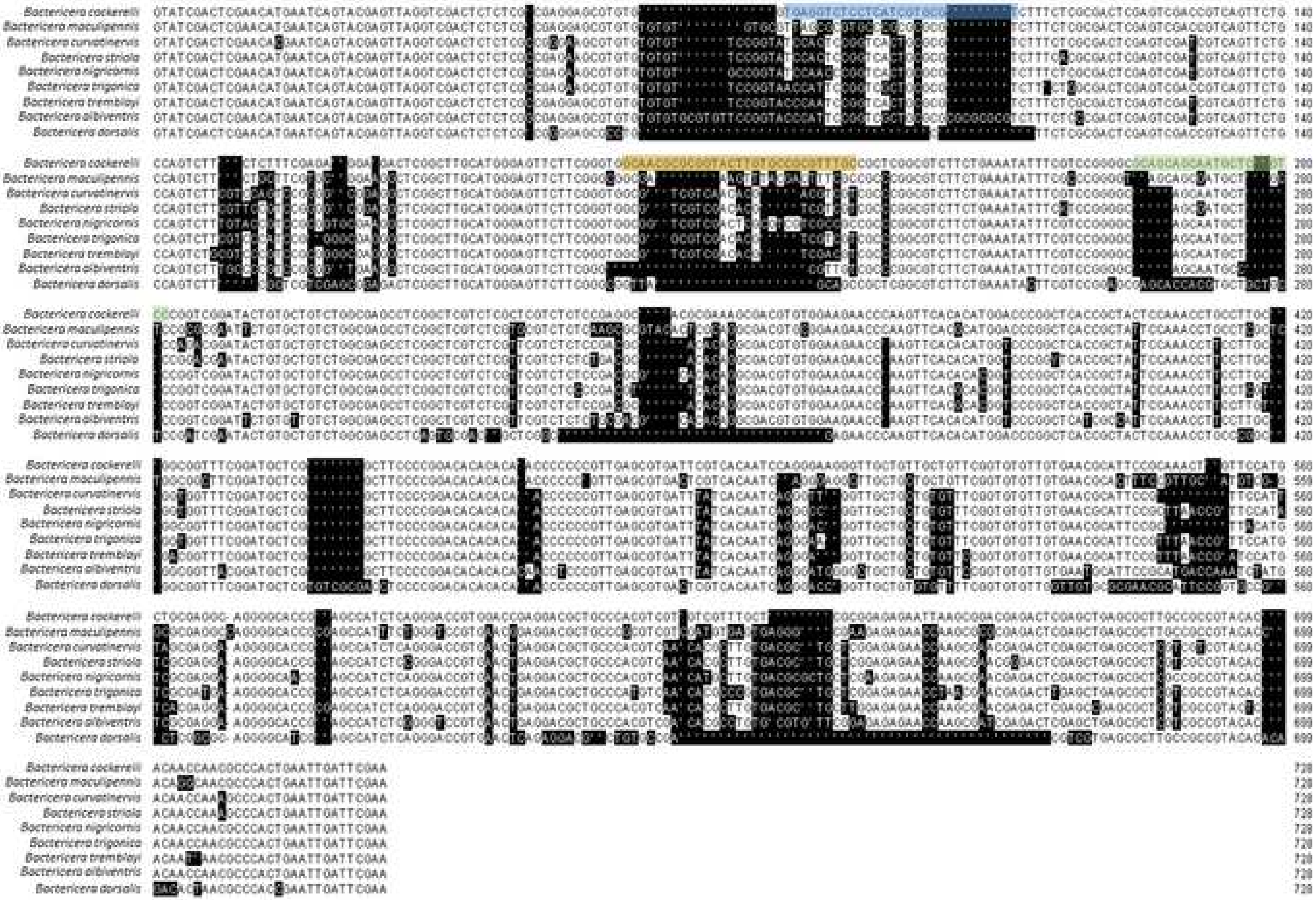

Sup tab S1

Click here to access/download
Supporting Information
Sup Tab S1.docx

Sup tab S2

Click here to access/download
Supporting Information
Sup Tab S2.docx

Sup tab S3

Click here to access/download
Supporting Information
Sup Tab S3.docx

Sup tab S4

Click here to access/download
Supporting Information
Sup Tab S4.docx

Full Title: A diagnostic real-time PCR assay for the rapid identification of the tomato-potato psyllid, *Bactericera cockerelli* (Šulc, 1909) and development of a psyllid barcoding database.

Authors

JC Sumner-Kalkun[1]*, MJ Sjölund[1], YM Arnsdorf[1], M Carnegie[1], F Highet[1], D Ouvrard[2,5], AFC Greenslade[3], JR Bell[3], R Sigvald[4], DM Kenyon[1]

[1] SASA, Roddinglaw Road, Edinburgh EH12 9FJ, UK. [2] Department of Life Sciences, Natural History Museum, Cromwell Road, London SW7 5BD, UK. [3] Rothamsted Insect Survey, Rothamsted Research, West Common, Harpenden, Hertfordshire, AL5 2JQ, UK. [4] Department of Ecology, Swedish University of Agricultural Sciences, Box 7044 750 07 Uppsala, Sweden. [5] Entomology and invasive plants Unit, Plant Health Laboratory, ANSES, 755 avenue du campus Agropolis, CS 30016, 34988 Montferrier-sur-Lez Cedex, France

*Corresponding author: jason.sumner-kalkun@sasa.gov.scot

Short Title: *Bactericera cockerelli* diagnostic assay

Keywords: *Bactericera cockerelli,* Biosecurity, Phytosanitary, Diagnostic, Pest, Vector, '*Candidatus* Liberibacter solanacearum', real-time PCR

Abstract

The accurate and rapid identification of many insect pests is an important step in the prevention and control of outbreaks in areas that are otherwise pest free. The potato-tomato psyllid *Bactericera cockerelli* (Šulc, 1909) is the main vector of '*Candidatus* Liberibacter solanacearum' on potato and tomato crops in Central and Northern America and New Zealand. This study describes the design and validation of the first species-specific TaqMan probe-based real-time PCR assay, targeting the ITS2 gene region of *B. cockerelli*. The assay successfully detected *B. cockerelli* genomic DNA from adults (100% accuracy, n=72); immatures (100% accuracy, n=26) and eggs (100% accuracy, n=25). This assay also detected DNA from cloned plasmids containing the ITS2 region of *B. cockerelli* (100% accuracy, n=24). The assay showed 0% false positives when tested on genomic and cloned DNA from 73 other psyllid species collected from across Europe, New Zealand and Mexico. This included 8 other species in the *Bactericera* genus and the main vectors of '*Candidatus* Liberibacter solanacearum' worldwide. ~~The assay can amplify *B. cockerelli* DNA across a range of $MgCl_2$ concentrations (1.5,3.5,5.5,7.5,9.5 µM), primer concentrations (0.1,0.2,0.3,0.5,1.0 µM) and annealing temperatures (58,60,62,64,66,68 °C).~~ The limit of detection for this assay at optimum conditions was 0.000001ng DNA (~200 copies) of ITS2 DNA which equates to around a 1:10000 dilution of DNA from one single adult specimen. ~~No significant differences were found between 6 x repeats of a 1:10 dilution series with 7 points when tested by two-way ANOVA ($F_{5, 25}$= 0.54, p = 0.955); two independent runs of experiments on two separate real-time PCR machines also showed no significant differences when tested by two-way ANOVA ($F_{1, 5}$= 1.28, p = 0.279).~~ This assay is the first real-time PCR

based method for accurate, robust, sensitive and specific identification of *B. cockerelli* from all life stages. It can be used as a surveillance and monitoring tool to further study this important crop pest and to aid the prevention of outbreaks, or to prevent their spread after establishment in new areas.

1. Introduction

The psyllid *Bactericera cockerelli* (Šulc, 1909), (commonly known as “Potato Psyllids” or “Tomato-Potato Psyllid”), is a major pest of cultivated Solanaceous crops including potato and tomato [1]. F~~The f~~eeding by~~of~~ this psyllid causes severe damage to potato plants including: deformed tubers; production of numerous small, poor quality tubers; curling of leaves and petioles; and yellowing or purpling of leaves. This leads to stunted growth and loss of yield [2]. *Bactericera cockerelli* is also the main vector of ‘*Candidatus* Liberibacter solanacearum’ (Lso) which is associated with Zebra Chip ~~and psyllid yellows~~ in Central and North America and New Zealand [3–8].

While *B. cockerelli* prefers to complete its life cycle on Solanaceous plants it ~~is also able to reproduce on~~can also complete development on species of Convolvulaceae (Bindweeds and Morning Glories) [9]. ~~There may also be an association with *B. cockerelli* and plants in the Lamiaceae (mints and deadnettles) but this is based on incidental observations from glasshouses with high *B. cockerelli* infection [10].~~ In addition, adult *B. cockerelli* have been found on over 40 species belonging to 20 families, however most of these are either casual, food or shelter plants on which the psyllid is unable to complete a full life cycle ~~and/or transmit Lso~~ [2,10–16]. Four biotypes of *B. cockerelli* have been described according to polymorphisms in the mitochondrial cytochrome *c oxidase subunit I* (COI) gene and represent geographically distinct populations; central, western, north-western, and south-

western [17,18]. Evidence suggest that these genetic types may ~~These populations have been observed to~~ differ in their ability to spread Lso [18,19].

Bactericera cockerelli is thought to originate from South-Western USA and Mexico [2,11] and from here has spread via natural and human-mediated dispersal to extend its range [20]. Outside America ~~in Northern and Central America and to Oceania where~~ it is now established in New Zealand [21] and more recently Western Australia [22]. ~~In Central America, *B. cockerelli* has been found as far south as Nicaragua [23] but has not currently been found in Costa Rica or Panama [24]. This psyllid has now established small populations in Canada [25] where previously it was not thought to overwinter. After a period of surveillance, in 2017, *B. cockerelli* was found in areas of Western Australia [22], however Lso has not yet been detected in psyllids or plants in these locations. In 2017, all developmental stages of *B. cockerelli* were found in two locations in Ecuador on potato plants. This represents the first finding of *B. cockerelli* in South America [26].~~

The phloem-limited bacterium ‘*Candidatus* Liberibacter solanacearum’ (Lso) is a pathogen associated with Zebra Chip disease of potatoes [3,23–25] and disease in other Solanaceous crops such as cultivated tomato [1,3,26,27], pepper [28], eggplant [29], tobacco [30,31] and tomatillo [26]. Currently, *B. cockerelli* is the main vector of Lso in field and glasshouse-grown Solanaceous plants in the United States, Mexico, areas of Central America [27–30], Canada [32], ~~and~~ New Zealand [5,6,25] and recently Ecuador [33]. Ten Lso haplotypes have been described, only three of which are associated with disease in Solanaceous plants. ~~While 9 haplotypes of Lso have hitherto been described in the literature (A, B, C, D, E, F, G, H, and U) only three of the haplotypes, A, B and F, are associated with disease in Solanaceous plants.~~ Haplotypes A, B, and F are associated with Zebra chip disease in America [3,34,35], whereas

only haplotype A has been found in New Zealand [5,36]. Haplotype B has also been found in *Bactericera maculipennis* (Crawford) [37]. The remaining haplotypes are not vectored by *B. cockerelli* but by closely related species in the Triozidae family. Haplotype G was found in 49 year-old herbarium specimens of *Solanum umbelliferum* [39] but it is not known if it is able to infect potato. Haplotypes C, D, and E are associated with disease in Apiaceous crops in Europe [28,40–43] and Northern Africa [44,45]. Lso Haplotypes D and E are vectored mainly by *Bactericera trigonica* and to a lesser extent *Bactericera nigricornis* and *Bactericera tremblayi* [42,45,46]. These closely related *Bactericera* spp. are morphologically similar and substantial taxonomic expertise is required to accurately identify species. Currently the geographic distribution of *B. cockerelli* does not overlap with *B. trigonica* and *B. nigricornis* which makes field identification easier. However, in the event of an outbreak of *B. cockerelli* molecular diagnostic methods allow rapid identification by non-specialists and is particularly valuable in areas where other *Bactericera* spp. co-occur.

Haplotype C is found in Northern Europe where it is mainly vectored by *Trioza apicalis* which, together with psyllid feeding, causes up to 100% loss of Apiaceaous crops in Finland [47,48] and Sweden [28] following transmission. Haplotype U is found in asymptomatic *Urtica dioica* (stinging nettle) and is vectored by the psyllid *Trioza urticae* but is not yet known to cause economic damage [49]. It seems that psyllid-mediated infection of host plants with Lso only occurs when the psyllid vector is on its preferred host. Infection of carrot plants with Lso haplotypes vectored by *B. cockerelli* and infection of potato plants by carrot psyllid vectored Lso only occurs very rarely *in-vitro* and suggests that an efficient vector is necessary for wide-scale spread of Lso [46]. Monitoring and prevention of the

Formatted: Space After: 22 pt, Adjust space between Latin and Asian text, Adjust space between Asian text and numbers

~~spread of *B. cockerelli* is essential to prevent the risk of an outbreak of Lso on potato, tomato and other Solanaceous crops in areas where it is not currently found [38].~~

The impact of *B. cockerelli* and associated Lso transmission on agriculture is significant. Since its arrival in New Zealand circa 2005 via human-mediated dispersal it has caused millions of dollars of economic losses [6,21]. Similarly, management of *B. cockerelli* in the US is reported to have cost millions of dollars per year in major potato growing areas such as Texas [38] and the Pacific North-West [39]. ~~In 2008, the reduction in exports of *capsicum* and losses in exports of tomato due to closure of New Zealand's export markets were estimated to have cost $NZ 5.22 million and $NZ 3 million respectively [21]. Direct crop losses caused by symptoms of Lso on tomato in glasshouse were estimated at $NZ 1 million [6]. Within 3 years in the mid 2000s the management of *B. cockerelli* in Texas was estimated to have cost $US 25.86 million [39]. Control of *B. cockerelli* in the Pacific North-West, where over 50% of US potatoes are grown, costs approximately $US 11 million a year [40].~~ The introduction of *B. cockerelli* into potato growing regions in Europe or Asia would be devastating to the agricultural industry of those regions. If *B. cockerelli,* or a sufficient vector of Solanaceous Lso haplotypes, were to invade Europe it is estimated that the effects of Lso damage on potato and tomato would cost € 222 million per year and the negative impact of social welfare could cost an additional estimated € 114 million [40].

Currently, *B. cockerelli* is considered an A1 quarantine pest in the EPPO region ~~and is not present in any part of the region~~ [4]. Consignments of aubergine and *Capsicum* from Mexico ~~that were~~ infested with immature~~s~~ and adult stage~~s~~ of *B. cockerelli* were intercepted four times during UK border inspections between 2017-2018~~at Heathrow Airport (London, England, UK) in 2017~~;~~,~~ indicating that there is a real threat of this pest making an incursion

Formatted: Font: Italic

Formatted: Font: Italic

into the EPPO region if not properly monitored [41]. Monitoring and prevention of the spread of *B. cockerelli* is essential to prevent the risk of an outbreak of Lso on potato, tomato and other Solanaceous crops in areas where it is not currently found [42]. There is therefore an evident need for a rapid and accurate diagnostic test to identify *B. cockerelli* at all life stages not only as a tool to support import inspections, but also to assist monitoring, eradication and control strategies. is evidentMonitoring and prevention of the spread of *B. cockerelli* is essential to prevent the risk of an outbreak of Lso on potato, tomato and other Solanaceous crops in areas where it is not currently found [38]. . It will assist the prevention of accidental introductions via human-mediated dispersal and, in the event of an existing outbreak, will support the rapid identification of this pest and subsequent control strategies.

We designed a species-specific real-time PCR diagnostic assay to detect all life-stages of *B. cockerelli*, eggs, immatures and adults. The assay provides a rapid diagnostic test to quickly determine the presence of *B. cockerelli*, allowing for the early detection of invasions/introductions and aiding in the prevention of spread of this psyllid.

2. Materials and Methods

2.1. Specimen collection

The assay was tested on 28 target adults *B. cockerelli* specimens and 73 non-target species consisting of 110 specimens see results section 3.1 for more info on samples(Supp Tab. S1). The classification follows Burckhardt & Ouvrard [43], and a complete taxonomic account of each species is given in Ouvrard [20]. Psyllid identifications were confirmed against reference type specimens in the NHM London collections. To account for intraspecific

genetic variation, we obtained *B. cockerelli* specimens from Mexico (Universidad Autónoma Agraria Antonio Narro) and USA (USDA, Agricultural Research Services) from colony collections of each of the four recognised biotypes of *B. cockerelli* in Central America, the Central, Western, North-Western, and South-Western biotypes [19]. Specimens of *B. cockerelli* were also obtained from New Zealand lab-reared colonies (Plant Research, New Zealand). Non-target specimens were mainly obtained from 12.2 m suction-traps in the United Kingdom that form part of the Rothamsted Insect Survey network described here [44]. Specimens were also obtained from suction-traps in Finland, Germany, Spain and Sweden; as well as from field collections from Finland, Israel, Mexico, Serbia, Spain, UK and USA. Non-target specimens from different regions of the USA were used to test assay specificity on species that are commonly found in the same region as *B. cockerelli*. As immatures and eggs are the most likely life stages that inspectors might find on imported plant material, we also tested the assay on DNA extracted from immatures and eggs from Mexico and the USA for validation.

2.2. DNA extraction, PCR, and DNA sequencing for identification of psyllids

DNA for sequencing and assay validation was extracted from psyllids using a non-destructive method first described in [45] and adapted from [46]. Psyllid specimens were preserved in 95% Ethanol: 5% Glycerol solution. Using a 15mm long, 0.15mm diameter stainless steel entomological head-less pin (A3 size, Watkins and Doncaster) mounted in a holder, specimens were initially pierced fully through the abdomen and half-way through the thorax from the dorsal side while attempting to minimise damage to head, legs, wings, terminalia and other body parts that are used for taxonomic identification. Pierced ~~Micro-dissected~~ specimens were placed in a microcentrifuge tube containing 180 µl of ATL buffer and 20 µl

of proteinase-k as outlined in the DNeasy Blood and Tissue Kit from Animal Tissues (Qiagen). Samples were placed in a shaking incubator over-night (~8-10 hrs) at 56 °C at 300 rpm. The protocol for DNA extraction in DNeasy Blood and Tissue Kit Protocol from Animal Tissues (Qiagen) was followed and the psyllid integument voucher specimen was stored in 95% Ethanol: 5% Glycerol for morphological identification. Psyllids were DNA barcoded using one or two gene regions. The internal transcribed spacer 2 (ITS2) and cytochrome c oxidase subunit 1 (CO1) were amplified and sequenced for identification of different psyllid species. For amplification of ITS2 primers CA55p8sFcm-F and CA28sB1d-R [47] were used; and for amplification of CO1 gene regions arthropod barcoding Primers LCO1490 and HCO2198 [48] were used. All reactions were performed in 20 µl consisting of: 10 µl 2x Type-It Microsatellite PCR Kit Master Mix (Qiagen); 0.2~~4~~ µM ~~µl (10 µM stock)~~ each forward and reverse primer; 7.2 µl molecular grade water (Sigma-Aldrich) and 2 µl of psyllid template DNA. Reactions were run on a Veriti 96-well thermal cycler (Applied Biosystems) using the following programs. ITS2: 95°C for 5 mins; 25 x cycles of (95°C for 30 s, 56°C for 90 s, 72°C for 30 s); and a final extension at 72°C for 10 mins. CO1: 94°C for 5 mins; 5 x cycles of (94°C for 30s, 45°C for 30s, 72°C for 1 min); 25 x cycles of (94°C for 30s, 51°C for 1 min, 72°C for 1 min); and a final extension of 72°C for 10 mins. PCR amplified gene regions were cleaned-up using EXO-SAP and Ethanol precipitation, then sequenced using the BigDye Terminator Cycle Sequencing Kit (Applied Biosystems), forward and reverse complimentary DNA strands were sequenced separately for each sample and analysed using a 3500xL Genetic Analyser (Applied Biosystems).

2.3. Bioinformatics and Real-Time PCR Assay Design

Sequence editing, assembly and alignment were performed on ".AB1" trace files uploaded to Geneious R11 v 11.1.5 (Biomatters Ltd.). Contigs were assembled after trimming sections of low-quality sequence and aligning the complimentary strands using CLUSTAL-W multiple sequence alignment method [49]. Final contigs for each species and each gene region were aligned to identify variable areas suitable as targets for *B. cockerelli* specific primer and probe sets. Primers and probes were designed using manual selection of target-specific regions analysed using the "Basic Local Alignment Search Tool" (BLAST) [50] against the NCBI GenBank database [51] and processing of selected regions for suitability/ specificity in "Primer3" [52] and "Primer-BLAST" software [53]. Primer annealing temperature, hairpin formation, self-complementarity, GC content and were assessed using "Primer3" [52]. Potential amplification of non-specific insect species was checked using Primer BLAST which includes all psyllid species present in the GenBank database. Primer and probe sets were selected/rejected based on the following parameters: primer annealing temperature 59-62°C; primer annealing temperature + 8-10°C for probe annealing temperature; no more than 2°C difference in annealing temperature between primers, max probe length 30bp, no more than 3 Gs in a row in probe, amplicon length max 300bp and specificity to *B. cockerelli.*

2.4. Real-time PCR Set-up and Standards

To calculate standard curves DNA standards of *B. cockerelli* were prepared using dilution series of linearized cloned plasmid DNA. DNA was extracted as above using the non-destructive method, amplified and cloned into competent *Escherichia coli* cells using the TOPO TA cloning kit (Thermo-Fisher). DNA from successfully transformed colonies was extracted using "PureYield Plasmid Miniprep System" (Promega). For assay validation DNA was cloned from other psyllid species (see results section 3.1)~~(Supp Tab. S1)~~. Stock DNA 10

ng/µl was linearised from cloned plasmid DNA using EcoRI restrictions enzyme (New England Biolabs), 0.5 µl of enzyme was added to 100 µl of stock DNA, this solution was incubated in a heat block (Thermomixer C, Eppendorf) at 37 °C for 15 mins. The enzyme was then deactivated at 65 °C for 20mins. Real-time PCRs were performed in 15 µl volumes including: 6.75 µl Jumpstart Taq Ready Mix (Sigma); 1.2 µl $MgCl_2$ (25mM); 0.45 µl of each primer; 0.15 µl probe; 4 µl of molecular grade water (Sigma); and 2 µl of template DNA. The standard real-time PCR cycle program was as follows. Hold stage: 50 °C for 2 mins then; 95 °C for 10 mins. PCR stage: 40 cycles of (95 °C for 15 secs; X °C for 1 min), with primer annealing temperature X being 58, 60, 62, 64, or 68; depending on the experiment. Primer concentration, $MgCl_2$ concentration and temperature was adjusted for validation and optimization of the assay as described below. Reactions were performed on a “QuantStudio 6 Flex” (Applied Biosystems) real-time PCR machine and analysis was done on the “QuantStudio Real-Time PCR Software” (Applied Biosystems).

2.5. Assay validation

2.5.1. Specificity

The final primer and probe set was tested on genomic DNA from 47 *B. cockerelli* specimens from different life stages. These included the 4 US biotypes [17,54] and specimens from New Zealand to determine false negatives. The assay was tested for specificity against genomic DNA of 73 non-target psyllid species collected as mentioned above, to detect false positives. This included a total of 8 other closely related *Bactericera* spp. and the major vectors of Lso on Apiaceous crops (*B. nigricornis*, *B. trigonica* and *Trioza apicalis*). Information regarding samples tested is in results section 3.1. The assay was also checked for cross-reaction against potato genomic DNA (*Solanum tuberosum*), 3 samples of *S.*

tuberosum 'Maris Piper' were tested in replicates of 8. ~~All information on specimens and DNA samples can be found in (Supp Tab. S1).~~ All reactions with non-target ~~psyllid~~ DNA were run in conjunction with a TaqMan Exogenous Internal Positive Control Reagent Kit (Applied Biosystems) to rule out the possibility that~~ensure~~ false positives were not obtained due to inhibition within the reaction. DNA from all non-target psyllids was sequenced in either ITS2, CO1 or both ~~-~~to ensure psyllid DNA was present in all reactions to rule out false negatives due to inefficient DNA extraction. Reactions were performed in duplicate at least, with a higher number of replicates for species closely related to *B. cockerelli* ~~(Supp Tab. S1)~~. False positives were defined as reactions with non-target DNA that showed fluorescence above the cycle threshold during 40 cycles; and false negatives were defined as reactions with *B. cockerelli* DNA that did not give a C_t after 40 cycles.

2.5.2. Sensitivity

Experiments were performed to determine the limit of detection of the assays. DNA standards were produced using *B. cockerelli* linearized cloned DNA from the ITS2 region. A nine point 10-fold dilution series starting with 10 ng/µl DNA up to $10^{\wedge -8}$ ng/µl of linearised plasmid DNA and genomic DNA was used to determine the limit of detection. 100ng/µl stock DNA concentration was initially checked using QuBit 4 Fluorometer (Invitrogen) and 5 µl was added to 45 µl of molecular grade water (Sigma-Aldrich) to dilute 1:10; eight~~6~~ subsequent dilutions were made. ~~Stock DNA 10 ng/µl was linearised using EcoRI restrictions enzyme (New England Biolabs), 0.5 µl of enzyme was added to 100 µl of stock DNA, this solution was incubated in a heat block (Thermomixer C, Eppendorf) at 37 °C for 15 mins. The enzyme was then deactivated at 65 °C for 20mins.~~ Linearised and non-linearised DNA was compared along with genomic DNA. The ability of the assay to detect immatures and eggs

was also tested. DNA from various instars of immatures was extracted using the non-destructive protocol described above. Batches of 1 egg, 5 eggs and 10 eggs were extracted using the DNeasy Blood & Tissue kit (Qiagen) and initially broken with a pestle.

2.5.3. Repeatability and Reproducibility

Variation in the performance of the assay between runs and within runs was assessed at a 0.2 µM primer concentration, with 1.5mM $MgCl_2$, and 60 °C annealing temperature. Linearised plasmid DNA from *Escherichia coli* transformed with *B. cockerelli* ITS2 DNA was used. A six point 1:10 dilution series starting at 10ng/µl was used with each dilution being performed in triplicate. The same experiment was repeated 3x simultaneously. Runs and variations between the three experiments were recorded and analysed using QuantStudio 6 Real-Time PCR Software. An identical plate following the same plate set-up and reaction mix was run simultaneously on another QuantStudio 6 real-time PCR machine to compare inter-run variation.

2.5.4. Robustness/Optimization

Amplification of target DNA, specificity and sensitivity at different $MgCl_2$ concentration, primer concentrations and annealing temperatures were performed to assess robustness. The assay was tested with 1.5, 3.5, 5.5, 7.5 and 9.5mM $MgCl_2$ concentration. For primers, 0.1, 0.2, 0.3, 0.5 and 1.0 µM concentrations were tested. The assay was also tested at different annealing temperatures 58, 60, 62, 64, 68 °C across. For each tested parameter, optimization was performed across a nine~~n eight~~ point 1:10 dilution series starting at 10ng/µl DNA. All samples were tested in triplicates. Closely related *Bactericera* species were included in these assays to assess specificity under different assay conditions. After

optimization of the assay a multifactorial robustness test was performed across two different real-time PCR machines to test the combined effects of small changes/errors in the PCR set-up. The assays were run on a “QuantStudio 6 Flex” (Applied Biosystems) and “CFX96 Real-Time System” (BioRad); results were analysed using “QuantStudio 6 Real-Time PCR Software” (Applied Biosystems) and “CFX Manager 3.1” (BioRad). The methodology used followed the European Network of GMO Laboratories (ENGL) recommendations [55].

3. RESULTS

3.1. DNA extraction, PCR, and DNA sequencing for identification of psyllids

DNA from 110 psyllid specimens comprising 73 different species were extracted, amplified and sequenced successfully from either CO1 or ITS2 gene regions, or both (~~Supp~~ Tab. ~~S~~1).

Family	Genus	Species	Voucher ID	Collection Location	Collection method	CO1 Ac#	ITS2 Ac#	Tech Reps	Vouc he
Aphalaridae	Aphalara	avicularis	160718.A.avi.23	Wellesbourne, UK	suction trap	MT021761	/	2	1
		polygoni	160718.A.pol.22	Wellesbourne, UK	suction trap	/	MT038907	2	1
	Blastopsylla	occidentalis	180312.Bl.occ.24	Salamanca, Spain	suction trap	MN272146	MN316692	2	3
	Craspedolepta	gutierreziae	160825.5 US	Nevada, USA	field collection	MT021786	MT038962	2	1
		minutissima	160825.1 US	Nevada, USA	field collection	MT021787	MT038963	2	1
			160825.10 US	Oregon, USA	field collection	MT021789	/	2	1
			160825.4 US	Nevada, USA	field collection	MT021788	/	2	1
		nervosa	160728.Cra.ner.2	Gogarbank, UK	suction trap	MT021790	MT038964	2	1
		pinicola	160825.2 US	Nevada, USA	field collection	/	MT038965	2	1
		subpunctata	160421.C.sub.5	Gogarbank, UK	suction trap	MT021791	MT038966	2	1
	Rhinocola	aceris	151014.R.ace.14	Wellesbourne, UK	suction trap	MT021810	MT038979	2	2
Liviidae	Diaphorina	citri	160309.D.cit.6	Lab Colony,	Lab Reared	MT021794	MT038969	2	1
	Euphyllura	olivina	180125.Eup.oli.3	Plants imported	on imported Olea	MT021797	MT038970	2	3
	Livia	crefeldensis	180312.L.cre.5	Salamanca, Spain	Suction trap	MN316678	MN272127	2	3
		junci	160404.L.jun.1	Broom' s Barn, UK	suction trap	MT021801	/	2	2
		opaqua	160825.6 US	Nevada, USA	field collection	MT021802	MT038973	2	1
Psyllidae	Arytaina	genistae	151203.A.gen.2J	Ayr, UK	suction trap	/	MT038909	2	1
	Arytainilla	gredi	180312.A.gre.1	Salamanca, Spain	suction trap	MN272123	MN316677	2	3
		spartiophila	180716.A.spa.29	SASA, Edinburgh,	suction trap	MT021762	MT038908	2	3
	Baeopelma	foersteri	151203.B.foe.1J	Ayr, UK	suction trap	/	MT038944	2	1
		foersteri	160928.B.foe.2	SASA, UK	suction trap	MT021776	/	2	1
	Cacopsylla	affinis	151203.C.aff.1	Wye, UK	suction trap	MT021777	MT038945	2	2
		ambigua	160404.C.amb.4	Wye, UK	suction trap	/	MT038946	2	2
		ambigua	161024.C.amb.3	Preston, UK	suction trap	/	MT038947	2	1
		americana	160825.3 US	Nevada, USA	field collection	MT021778	MT038948	2	1
		brunneipennis	160309.C.bru.8	Wye, UK	suction trap	/	MT038949	2	2
		crataegi	160404.C.cra.3	Broom' s Barn, UK	suction trap	MT021779	MT038950	2	2
		mali	180910.C.mal.30	Elcho Castle	field collection	/	MT038951	2	3
		melanoneura	160718.C.mel.6	Kirton, UK	suction trap	/	MT038952	2	3
		moscovita	190109.C.mos.1	Quedlinburg,	suction trap	/	/	2	3
		peregrina	161024.C.per.11	Silwood Park, UK	suction trap	MT021780	MT038953	2	1
		pruni	160203.C.pru.18	Wellesbourne, UK	suction trap	/	MT038954	2	2
		pulchra	160718.C.pul.15	Elgin, UK	suction trap	/	MT038955	2	1
		pyricola	160203.C.pco.2	Wye, UK	suction trap	MT021781	MT038956	2	2
		saliceti	161024.C.sal.7	York, UK	suction trap	/	MT038958	2	1
		sorbi	161024.C.sor.8	Preston, UK	suction trap	MT021782	MT038959	2	1
		rhamnicola	151014.C.rha.8	Wellesbourne, UK	suction trap	/	MT038957	2	2
		ulmi	171011.C.ulmi.1	Quedlinburg,	suction trap	MT021783	MT038960	2	3
	Ceanothia	ceanothi	160825.9 US	Oregon, USA	field collection	MT021784	/	2	1
	Chamaepsylla	hartigii	160728.Ch.har.1	Gogarbank, UK	suction trap	MT021785	MT038961	2	1
	Euglyptoneura	fuscipennis	160825.7 US	Oregon, USA	field collection	MT021795	/	2	1
		robusta	160825.8 US	Oregon, USA	field collection	MT021796	/	2	1
	Heteropsylla	texana	160825.11 US	Texas, USA	field collection	MT021798	/	2	1
	Psylla	alni	161019.P.aln.1	Sweden	suction trap	MT021804	/	2	1
		buxi	180622.P.buxi.22	Scotland, UK	suction trap	MT021806	MT038976	2	3
		betulae	161123.P.bet.20	Jokioinen, Finland	suction trap	MT021805	MT038975	2	3
	Psyllopsis	discrepans	151002.P.dis.8	Sweden	suction trap	MT021807	/	2	1
		fraxini	180716.P.fri.33	SASA HQ,	suction trap	MT021808	MT038977	2	3
		fraxinicola	160203.P.fra.6	Wellesbourne, UK	suction trap	MT021809	MT038978	2	2
	Spanioneura	fonscolombii	180802.S.fon.29	SASA HQ,	field collection	/	MT038980	2	3
Spondyliaspidae	Ctenarytaina	spatulata	160404.Ct.spa.6	Wye, UK	suction trap	MT021792	MT038967	2	2
		spatulata	161024.Ct.spa.5	Wye, UK	suction trap	MT021793	MT038968	2	1
Triozidae	Bactericera	albiventris	171214.B.alb.11	Jokioinen, Finland	suction trap	/	MT038910	5	3
		curvatinervis	161123.B.cur.42	Jokioinen, Finland	suction trap	/	MT038911	5	3
		dorsalis	160803.B.dor.2	Florida, USA	lab colony	MT021763	MT038912	5	3
		maculipennis	190604.B.mac.1	Lab Colony, USA	Lab Reared	/	MT038913	2	3
			190604.B.mac.2	Lab Colony, USA	Lab Reared	/	MT038914	2	3
			190604.B.mac.3	Lab Colony, USA	Lab Reared	/	MT038915	2	3
			190604.B.mac.4	Lab Colony, USA	Lab Reared	/	MT038916	2	3
			190604.B.mac.5	Lab Colony, USA	Lab Reared	/	MT038917	2	3
			190604.B.mac.6	Lab Colony, USA	Lab Reared	/	MT038918	2	3
			190604.B.mac.7	Lab Colony, USA	Lab Reared	/	MT038919	2	3
		nigricornis	170324.B.nig.18	Spain	field collection	MT021764	MT038920	5	3
			170324.B.nig.22	Spain	field collection	MT021765	MT038921	5	3
		salicivora	190116.B.sal.1	Elgin, Scotland,	suction trap	/	/	6	3
		striola	161123.B.str.9	Jokioinen, Finland	suction trap	/	MT038922	-	
		tremblayi	170731.B.tre.5	Slanci, Belgrade,	field collection	/	MT038923	5	3
			190604.B.trem.1	Spain	Lab Colony	/	MT038924	2	3
			190604.B.trem.1	Spain	Lab Colony	/	MT038925	2	3
			190604.B.trem.1	Spain	Lab Colony	/	MT038926	2	3
			190604.B.trem.2	Spain	Lab Colony	/	MT038927	2	3
			190604.B.trem.2	Spain	Lab Colony	/	MT038928	2	3
		trigonica	170629.B.tri.16	Tunisia	field collection	MT021766	MT038929	3	3
			170629.B.tri.17	Tunisia	field collection	/	MT038930	3	3
			170629.B.tri.18	Tunisia	field collection	MT021767	MT038931	3	3

Formatted Table

		181010.B.tri.17	Spain	Lab Colony	MT021768	MT038932	2	3
		181010.B.tri.18	Spain	Lab Colony	MT021769	MT038933	2	3
		181010.B.tri.19	Spain	Lab Colony	/	MT038934	2	3
		181010.B.tri.20	Spain	Lab Colony	MT021770	MT038935	2	3
		181010.B.tri.21	Spain	Lab Colony	/	MT038936	2	3
		190604.B.tri.23	Spain	Lab Colony	MT021771	MT038937	2	3
		190604.B.tri.24	Spain	Lab Colony	/	MT038938	2	3
		190604.B.tri.25	Spain	Lab Colony	MT021772	MT038939	2	3
		190604.B.tri.26	Spain	Lab Colony	MT021773	MT038940	2	3
		190604.B.tri.27	Spain	Lab Colony	MT021774	MT038941	2	3
		190604.B.tri.28	Spain	Lab Colony	/	MT038942	2	3
		190604.B.tri.29	Spain	Lab Colony	MT021775	MT038943	2	3
Heterotrioza	*chenopodii*	160203.H.che.11	Kirton, UK	suction trap	/	MT038971	2	2
		160825.12 US	Washington, USA	field collection	MT021799	/	2	1
Lauritrioza	*alacris*	160816.L.ala.2	Spain	suction trap	MT021800	MT038972	2	1
Powellia	*vitreoradiata*	161024.P.vit.10	Kirtkon, UK	suction trap	MT021803	MT038974	2	1
Trioza	*albifrons*	160825.18.US	Nevada, USA	field collection	MT021811	MT038981	2	1
	anthrisci	150708.T.ant.11	Jokionen, Finland	field collection	MT021812	/	2	3
	apicalis	161019.T.api.5	Sweden	field collection	MT021813	/	2	3
	buxtoni	170324.T.bux.11	Israel	field collection	MT021814	MT038982	2	3
	centranthi	161024.T.cen.9	Wye, UK	suction trap	MT021815	/	2	1
	cerastii	171214.T.cer.32	Vikki, Finland	suction trap	MT021816	MT038983	2	3
	dispar	160718.T.disp.26	Hellfreda, Sweden	suction trap	MT021817	/	2	1
	erytreae	160808.ICA.19	Spain	Lab Colony	/	MT038984	2	1
	flavipennis	160421.T.fla.3	Sweden	suction trap	MT021818	MT038985	2	1
	galii	160203.T.gal.23	Wellesbourne, UK	suction trap	/	MT038986	2	2
	remota	160718.T.rem.8	Sweden	suction trap	/	MT038987	2	1
		180424.T.rem.1	Dundee, UK	Suction trap	MT021819	MT038988	3	3
		180424.T.rem.6	Dundee, UK	Suction trap	MT021820	MT038989	3	3
		180424.T.rem.16	Dundee, UK	Suction trap	MT021821	MT038990	3	3
		180424.T.rem.18	Dundee, UK	Suction trap	MT021822	MT038991	3	3
		180424.T.rem.19	Dundee, UK	Suction trap	/	MT038992	3	3
		190116.T.rem.7	UK	Suction trap	MT021823	MT038993	3	3
	rhamni	151002.T.rha.13	Sweden	suction trap	MT021824	MT038994	2	1
	tatrensis	160718.T.tat.27	Sweden	suction trap	/	MT038995	2	1
	urticae	160816.T.urt.17	Spain	field collection	/	MT038996	2	1

Table 1: Information on non-target psyllid species and plant specimens tested using the *B. cockerelli* real-time PCR assay Bcoc_JSK2 showing number of technical replicates and false positives. All non-target species gave 0% false positives. GenBank Accession numbers are included for ITS2 and CO1 regions if sequencing was successful. Voucher Location: 1= 1; 2= 2 Research Insect Survey; 3= SASA Hemipteran DNA Database. All DNA samples are stored in the SASA Hemipteran DNA database.

Formatted: Line spacing: 1.5 lines

3.2. Bioinformatics and Real-Time PCR Assay Design.

While differentiation within both the ITS2 and CO1 gene regions was sufficient to discriminate between psyllid species, the ITS2 gene region was more suitable for TaqMan assay design for *B. cockerelli*. Similarities between CO1 gene sequences between members of the *Bactericera* genus and *B. cockerelli* were higher than in the ITS2 region (average % similarity = 82.51 ± 0.68 for CO1 and 77.80 ± 4.79 for ITS2) (Tab. 2~~1~~). The ITS2 region showed larger sections of variability along the gene on which to design primers and probes.

Several primer and probe sets passed the selection criteria, but most were unsuitable due to high rate of false positives from closely related *Bactericera* species. The final primer and probe set Bcoc_JSK2 (Tab. 3~~2~~) targets a 187bp region of the ITS2 gene (Fig. 1).

Species	ITS2			CO1		
	% Similarity	bp	GC content %	% Similarity	bp	GC content %
B. trigonica	78.96	662	59.3	82.88	509	35.4
B. tremblayi	79.16	665	59.1	82.97	682	33
B. curvatinervis	80.30	655	58	82.23	678	34.7
B. nigricornis	81.16	668	59.3	81.28	521	36.7
B. albiventris	76.67	667	59.2	83.41	663	32.9
B. dorsalis	65.59	560	61.3	82.31	685	32.6
B. maculipennis	80.67	674	61.6	nd	nd	nd
B. salicivora	nd	nd	nd	nd	nd	nd
B. striola	79.91	663	59.1	nd	nd	nd
B.cockerelli	N/A	569	61.0	N/A	595	32.6

**Oligo Name**	**Function**	**Sequence 5'-3'**	**Tm**	**Length (bp)**

Bcoc_JSK2-f	forward primer	GAGGTCTCCTCATCGTGCGT	61	25
Bcoc_JSK2-r	reverse primer	GGACGAGCATTGCTGCTGC	62.2	23
Bcoc_JSK2-p	probe (FAM-BHQ)	GCAAACGCGGCACAAGTACCGCGC	70.9	25

Table 21. Closely related Bactericera species tested with Bcoc_JSK2 assay. ITS similarity = % identity to DNA sample 150727.B.coc.1. CO1 similarity = % identity to a consensus sequences of all *B. cockerelli* sequences obtained during this study. CO1 genes showed higher similarity and fewergenerally less conserved and variable regions compared to ITS2 regions. Highest % similarity in to *B. cockerelli* in the ITS2 region was found in *B. nigricornis* (81.16) and to *B. albiventris* in the CO1 region (83.41). The Bcoc_JSK2 assay does not give false positives with any of the species listed here. (nd= not determined due to sequencing failing).

Table 32. Final oligonucleotide sequences for the Bcoc_JSK2 TaqMan real-time PCR assay to identify *B. cockerelli*. The assay targets a 187 bp region of the ITS2 gene region.

Sample name	Life Stage	Origin	Ct ave	Tech reps	CO1 Ac#	ITS2 Ac#	DNA Source
181119.B.coc.06	1 egg	Mexico	29.80	2	/	MT027568	Genomic
191003.B.coc.01	1 egg	Mexico	33.41	3	/	MT027592	Genomic
191003.B.coc.02	1 egg	Mexico	24.95	3	/	MT027593	Genomic
191003.B.coc.03	1 egg	Mexico	33.79	3	/	MT027594	Genomic
191003.B.coc.04	1 egg	Mexico	22.43	6	/	MT027595	Genomic
181119.B.coc.07	5 eggs	Mexico	24.42	2	/	MT027569	Genomic
181119.B.coc.21	5 eggs	Mexico	28.32	2	/	MT027582	Genomic
181119.B.coc.08	10 eggs	Mexico	29.61	2	/	MT027570	Genomic
181119.B.coc.22	10 eggs	Mexico	26.43	2	/	MT027583	Genomic
181119.B.coc.03	immature	Mexico	22.56	2	/	MT027565	Genomic
181119.B.coc.04	immature	Mexico	22.33	2	/	MT027566	Genomic
181119.B.coc.05	immature	Mexico	21.46	2	/	MT027567	Genomic
181119.B.coc.11	immature	Mexico	23.16	2	/	MT027573	Genomic
181119.B.coc.12	immature	Mexico	24.15	2	/	MT027574	Genomic
181119.B.coc.13	immature	Mexico	23.94	2	/	MT027575	Genomic
181119.B.coc.14	immature	Mexico	25.75	2	/	MT027576	Genomic
181119.B.coc.16	immature	Mexico	23.49	2	/	MT027578	Genomic
181119.B.coc.18	immature	Mexico	22.45	2	/	MT027580	Genomic
181119.B.coc.19	immature	Mexico	23.50	2	/	MT027581	Genomic
190604.B.coc.13	immature	Mexico	24.96	2	/	MT027588	Genomic
190604.B.coc.14	immature	Mexico	25.09	2	/	MT027589	Genomic
190604.B.coc.15	immature	Mexico	28.37	2	/	MT027590	Genomic
150727.B.coc.02	Adult	South Western, USA	22.18	2	MT040955	MG719775	Genomic
150827.B.coc.02	Adult	South Western, USA	22.18	2	MT040956	MT027597	Genomic
150827.B.coc.03	Adult	Central USA	24.49	6	MT040957	MT027598	Genomic
150827.B.coc.04	Adult	North Western, USA	24.77	2	MT040958	MT027599	Genomic
150827.B.coc.06	Adult	North Western, USA	23.68	2	MT040960	MT027552	Genomic
150827.B.coc.12	Adult	Western, USA	20.39	2	MT040961	MT027596	Genomic
150827.B.coc.17	Adult	South Western, USA	19.65	2	MT040962	MT027553	Genomic
160725.B.coc.05	Adult	Central, USA	21.45	2	MT040963	/	Genomic
160726.B.coc.01	Adult	New Zealand	21.56	2	/	MT027557	Genomic
160726.B.coc.02	Adult	New Zealand	21.02	2	/	MT027558	Genomic
160726.B.coc.03	Adult	New Zealand	20.48	2	/	MT027559	Genomic
160726.B.coc.04	Adult	New Zealand	21.98	2	/	MT027560	Genomic
160726.B.coc.05	Adult	New Zealand	19.43	2	/	MT027561	Genomic
160726.B.coc.06	Adult	New Zealand	20.96	2	/	MT027562	Genomic
180731.B.coc.04	Adult	North Western, USA	24.42	6	MT040964	/	Genomic
180731.B.coc.05	Adult	Western, USA	22.91	6	MT040965	/	Genomic
180731.B.coc.06	Adult	Western, USA	27.14	6	MT040966	/	Genomic
181119.B.coc.01	Adult	Mexico	21.47	2	/	MT027563	Genomic
181119.B.coc.02	Adult	Mexico	19.98	2	/	MT027564	Genomic
181119.B.coc.09	Adult	Mexico	21.83	2	/	MT027571	Genomic
181119.B.coc.10	Adult	Mexico	19.48	2	/	MT027572	Genomic
181119.B.coc.15	Adult	Mexico	21.27	2	/	MT027577	Genomic
181119.B.coc.17	Adult	Mexico	23.74	2	/	MT027579	Genomic
190604.B.coc.09	Adult	USDA, Lab Colony	21.51	2	/	MT027584	Genomic
190604.B.coc.10	Adult	Mexico	20.33	2	/	MT027585	Genomic
190604.B.coc.11	Adult	Mexico	22.67	2	/	MT027586	Genomic
190604.B.coc.12	Adult	Mexico	24.37	2	/	MT027587	Genomic

190604.B.coc.16	Adult	Mexico	27.15	2	/	MT027591	Genomic
150827.B.coc.05.col.04	transformed *E. coli*	Lab	11.23	6	MT040959	MT027551	Cloned, 10ng
160725.B.coc.01.col.06	transformed *E. coli*	Lab	11.55	6	/	MT027554	Cloned, 10ng
160725.B.coc.06.col.04	transformed *E. coli*	Lab	11.78	6	/	MT027555	Cloned, 10ng
160725.B.coc.07.col.08	transformed *E. coli*	Lab	11.67	6	/	MT027556	Cloned, 10ng

~~Sample name~~	~~Life Stage~~	~~Origin~~	~~Ct ave~~	~~Tech~~	~~%~~	~~CO1 Accession~~	~~ITS2 Accession~~	~~DNA Type~~
~~181119.B.coc.06~~	~~1 egg~~	~~Mexico~~	~~29.80~~	~~2~~	~~100~~	~~/~~	~~TBC~~	~~Genomic~~
~~191003.B.coc.01~~	~~1 egg~~	~~Mexico~~	~~33.41~~	~~3~~	~~100~~	~~/~~	~~TBC~~	~~Genomic~~
~~191003.B.coc.02~~	~~1 egg~~	~~Mexico~~	~~24.95~~	~~3~~	~~100~~	~~/~~	~~TBC~~	~~Genomic~~
~~191003.B.coc.03~~	~~1 egg~~	~~Mexico~~	~~33.79~~	~~3~~	~~100~~	~~/~~	~~TBC~~	~~Genomic~~
~~191003.B.coc.04~~	~~1 egg~~	~~Mexico~~	~~22.43~~	~~6~~	~~100~~	~~/~~	~~TBC~~	~~Genomic~~
~~181119.B.coc.07~~	~~5 eggs~~	~~Mexico~~	~~24.42~~	~~2~~	~~100~~	~~/~~	~~TBC~~	~~Genomic~~
~~181119.B.coc.21~~	~~5 eggs~~	~~Mexico~~	~~28.32~~	~~2~~	~~100~~	~~/~~	~~TBC~~	~~Genomic~~
~~181119.B.coc.08~~	~~10 eggs~~	~~Mexico~~	~~29.61~~	~~2~~	~~100~~	~~/~~	~~TBC~~	~~Genomic~~
~~181119.B.coc.22~~	~~10 eggs~~	~~Mexico~~	~~26.43~~	~~2~~	~~100~~	~~/~~	~~TBC~~	~~Genomic~~
~~181119.B.coc.03~~	~~immature~~	~~Mexico~~	~~22.56~~	~~2~~	~~100~~	~~/~~	~~TBC~~	~~Genomic~~
~~181119.B.coc.04~~	~~immature~~	~~Mexico~~	~~22.33~~	~~2~~	~~100~~	~~/~~	~~TBC~~	~~Genomic~~
~~181119.B.coc.05~~	~~immature~~	~~Mexico~~	~~21.46~~	~~2~~	~~100~~	~~/~~	~~TBC~~	~~Genomic~~
~~181119.B.coc.11~~	~~immature~~	~~Mexico~~	~~23.16~~	~~2~~	~~100~~	~~/~~	~~TBC~~	~~Genomic~~
~~181119.B.coc.12~~	~~immature~~	~~Mexico~~	~~24.15~~	~~2~~	~~100~~	~~/~~	~~TBC~~	~~Genomic~~
~~181119.B.coc.13~~	~~immature~~	~~Mexico~~	~~23.94~~	~~2~~	~~100~~	~~/~~	~~TBC~~	~~Genomic~~
~~181119.B.coc.14~~	~~immature~~	~~Mexico~~	~~25.75~~	~~2~~	~~100~~	~~/~~	~~TBC~~	~~Genomic~~
~~181119.B.coc.16~~	~~immature~~	~~Mexico~~	~~23.49~~	~~2~~	~~100~~	~~/~~	~~TBC~~	~~Genomic~~
~~181119.B.coc.18~~	~~immature~~	~~Mexico~~	~~22.45~~	~~2~~	~~100~~	~~/~~	~~TBC~~	~~Genomic~~
~~181119.B.coc.19~~	~~immature~~	~~Mexico~~	~~23.50~~	~~2~~	~~100~~	~~/~~	~~TBC~~	~~Genomic~~
~~190604.B.coc.13~~	~~immature~~	~~Mexico~~	~~24.96~~	~~2~~	~~100~~	~~/~~	~~TBC~~	~~Genomic~~
~~190604.B.coc.14~~	~~immature~~	~~Mexico~~	~~25.09~~	~~2~~	~~100~~	~~/~~	~~TBC~~	~~Genomic~~
~~190604.B.coc.15~~	~~immature~~	~~Mexico~~	~~28.37~~	~~2~~	~~100~~	~~/~~	~~TBC~~	~~Genomic~~
~~150727.B.coc.02~~	~~Adult~~	~~South Western,~~	~~22.18~~	~~2~~	~~100~~	~~/~~	~~TBC~~	~~Genomic~~
~~150827.B.coc.02~~	~~Adult~~	~~South Western,~~	~~22.18~~	~~2~~	~~100~~	~~/~~	~~TBC~~	~~Genomic~~
~~150827.B.coc.03~~	~~Adult~~	~~Central USA~~	~~24.49~~	~~6~~	~~100~~	~~/~~	~~TBC~~	~~Genomic~~
~~150827.B.coc.04~~	~~Adult~~	~~North Western,~~	~~24.77~~	~~2~~	~~100~~	~~/~~	~~TBC~~	~~Genomic~~
~~150827.B.coc.06~~	~~Adult~~	~~North Western,~~	~~23.68~~	~~2~~	~~100~~	~~/~~	~~TBC~~	~~Genomic~~
~~150827.B.coc.12~~	~~Adult~~	~~Western, USA~~	~~20.39~~	~~2~~	~~100~~	~~/~~	~~TBC~~	~~Genomic~~
~~150827.B.coc.17~~	~~Adult~~	~~South Western,~~	~~19.65~~	~~2~~	~~100~~	~~/~~	~~TBC~~	~~Genomic~~

Formatted Table

Formatted: Centered

Formatted: Centered

Formatted: Centered

Formatted: Centered

Formatted: Centered

Formatted: Centered

Formatted: Centered

Formatted: Centered

Formatted: Centered

Formatted: Centered

Formatted: Centered

Formatted: Centered

Formatted: Centered

Formatted: Centered

Formatted: Centered

Formatted: Centered

Formatted: Centered

Formatted: Centered

Formatted: Centered

Formatted: Centered

Formatted: Centered

Formatted: Centered

Formatted: Centered

Formatted: Centered

Formatted: Centered

Formatted: Centered

Formatted: Centered

Formatted: Centered

Formatted: Centered

160725.B.coc.05	Adult	Central, USA	21.45	2	100	/	TBC	Genomic
160726.B.coc.01	Adult	New Zealand	21.56	2	100	/	TBC	Genomic
160726.B.coc.02	Adult	New Zealand	21.02	2	100	/	TBC	Genomic
160726.B.coc.03	Adult	New Zealand	20.48	2	100	/	TBC	Genomic
160726.B.coc.04	Adult	New Zealand	21.98	2	100	/	TBC	Genomic
160726.B.coc.05	Adult	New Zealand	19.43	2	100	/	TBC	Genomic
160726.B.coc.06	Adult	New Zealand	20.96	2	100	/	TBC	Genomic
180731.B.coc.04	Adult	North Western,	24.42	6	100	/	TBC	Genomic
180731.B.coc.05	Adult	Western, USA	22.91	6	100	/	TBC	Genomic
180731.B.coc.06	Adult	Western, USA	27.14	6	100	/	TBC	Genomic
181119.B.coc.01	Adult	Mexico	21.47	2	100	/	TBC	Genomic
181119.B.coc.02	Adult	Mexico	19.98	2	100	/	TBC	Genomic
181119.B.coc.09	Adult	Mexico	21.83	2	100	/	TBC	Genomic
181119.B.coc.10	Adult	Mexico	19.48	2	100	/	TBC	Genomic
181119.B.coc.15	Adult	Mexico	21.27	2	100	/	TBC	Genomic
181119.B.coc.17	Adult	Mexico	23.74	2	100	/	TBC	Genomic
190604.B.coc.09	Adult	USDA, Lab Colony	21.51	2	100	/	TBC	Genomic
190604.B.coc.10	Adult	Mexico	20.33	2	100	/	TBC	Genomic
190604.B.coc.11	Adult	Mexico	22.67	2	100	/	TBC	Genomic
190604.B.coc.12	Adult	Mexico	24.37	2	100	/	TBC	Genomic
190604.B.coc.16	Adult	Mexico	27.15	2	100	/	TBC	Genomic
150827.B.coc.05.col.	transformed *E.*	Lab	11.23	6	100	/	TBC	Cloned,
160725.B.coc.01.col.	transformed *E.*	Lab	11.55	6	100	/	TBC	Cloned,
160725.B.coc.06.col.	transformed *E.*	Lab	11.78	6	100	/	TBC	Cloned,
160725.B.coc.07.col.	transformed *E.*	Lab	11.67	6	100	/	TBC	Cloned,

Table 43: Information on *Bactericera cockerelli* samples tested with Bcoc_JSK2 assay including genomic DNA from adults, immatures, single eggs and egg batches. Location of samples collection is also included. All samples gave 100% positives with the assay, samples were run in at least duplicate. GenBank accession numbers relating to sequenced CO1 and ITS2 (MT027551-MT027599) regions are included.

Figure 1. CLUSTAL-W alignment of ITS2 regions from closely related *Bactericera* species showing variable regions and the gene target for the Bcoc_JSK2 primer and probe set. Bases

Formatted: Centered
Formatted: Centered
Formatted: Centered
Formatted: Centered
Formatted: Centered
Formatted: Centered
Formatted: Centered
Formatted: Centered
Formatted: Centered
Formatted: Centered
Formatted: Centered
Formatted: Centered
Formatted: Centered
Formatted: Centered
Formatted: Centered
Formatted: Centered
Formatted: Centered
Formatted: Centered
Formatted: Centered
Formatted: Centered
Formatted: Centered
Formatted: Centered
Formatted: Centered
Formatted: Centered
Formatted: Centered
Formatted: Font: 12 pt
Formatted: Font: 18 pt

shades with black show differences to *B. cockerelli* sequence. Colour highlights locations of forward primer (blue highlight); reverse primer (green highlight) and probe (yellow highlight). The probe and reverse primer are reverse compliments of the highlighted regions here.

3.3. Specificity and Sensitivity

This assay did not amplify DNA from any of the 73 non-target psyllid species or *Solanum tuberosum* DNA when tested at 60 °C with primer concentration 0.2 µM~~/mol~~. ~~This~~ Samples included nine~~8~~ closely related *Bactericera* species with similar ITS2 and CO1 sequences (Tab. 2~~1~~). Under optimal conditions, false negatives = 0% for all non-target species tested with pure genomic DNA, giving a diagnostic specificity of 100%. Some suboptimal reaction conditions showed 33% false positives against high concentrations (10 ng / 1 ng) of *Bactericera albiventris* cloned DNA (see ~~as mentioned~~ below). All *B. cockerelli* genomic DNA samples gave positive results (Tab. 4~~3~~) giving 0% false negatives across 54 biological replicates and 147 technical replicates; resulting in a diagnostic sensitivity of 100%. These included *B. cockerelli* specimens from each of the four US biotypes as well as specimens from New Zealand. These specimens included adults, immature stages~~s~~ and eggs. The assay can amplify *B. cockerelli* DNA from both cloned and genomic samples. Under optimal conditions for PCR efficiency and specificity (60 °C, 0.2 µM primer, 1.5 mM $MgCl_2$) the limit of detection was 0.000001 ng DNA across a range of different reaction parameters this equates to 200 copy numbers of ITS2 calculated using the following equation: Number of Copies = (ng DNA x 6.022×10^{23}) ÷ ((length of ~~gene region in~~ plasmid (4656) + cloned fragment (700)bp~~ase pairs~~) * 1×10^9 * 660). The copy number calculator available at http://scienceprimer.com/copy-number-calculator-for-realtime-pcr was used. Diagnostic

sensitivity was 100% on all DNA extracted from *B. cockerelli* immatures. False negatives from DNA from egg extractions were 0% for single eggs and 0% for batches of 3 and 10 eggs.

3.4. Repeatability and Reproducibility

No significant differences were found between C_t means across the different replicates at different concentrations as tested by two-way ANOVA ($F_{5, 25}$= 0.54, p = 0.955). The assay also performed consistently across different machines and there was no significant difference between runs across the two machines as tested by two-way ANOVA ($F_{1, 5}$= 1.28, p = 0.279).

3.5. Robustness/Optimization

The assays amplified *B. cockerelli* DNA at all primer concentrations, $MgCl_2$ concentrations and annealing temperatures with varying levels of efficiency, precision, and sensitivity (Supp Tabs. S1~~2~~-S3~~4~~). At primer concentration~~,~~ 0.5 µM, the assay was less sensitive only amplifying down~~up~~ to 0.0001 ng DNA. At higher primer concentrations ~~(~~(~~0.5 and ~~1.0 µM,~~)~~) the assay showed higher sensitivity, but efficiency was outside the range for acceptable use. The assay performed optimally at 0.2 µM primer concentration showing good efficiency and high sensitivity (0.000001 ng DNA) (Supp Tab. S1~~2~~). Generally, standard deviation of the C_t was lower at higher DNA concentrations and some of the primer concentrations showed SD slightly above the accepted level for quantitative real-time PCR, however this module is intended for qualitative use. At high DNA concentrations all primer concentrations are suitable for use with Bcoc_JSK2 primer and probe set to detect *B. cockerelli* but 0.2 µM is recommended for best results. The assay did not amplify non-target DNA from the 8 other

Bactericera species tested at the different primer concentrations (0.1, 0.2, 0.3, 0.5 and 1.0 µM).

The MgCl2 concentration of the assay made only small differences to the overall performance of the assay (Supp Tab. S23) and the assay was able to amplify *B. cockerelli* DNA at low concentrations (0.000001 ng) at each $MgCl_2$ concentration. The precision of the assay was lower at higher $MgCl_2$ concentrations 7.56mM and 9.58mM (Supp Tab. S23).

Sensitivity was slightly higher at 64 °C giving 33.33% (n=3) positives for only 20 copies of *B. cockerelli* DNA (0.0000001 ng), however at 64 °C and 66 °C 33.33% (n=3) false positives were found with 10ng and 1 ng of *B. albiventris* cloned DNA (Supp Tab. S34). Reactions at 58 °C were 10 to 100-fold less sensitive than reactions at 6458 °C. For best sensitivity and specificity, it is suggested that assays using the Bcoc_JSK2 primer and probe set should be performed at 60 °C or 62 °C. While higher temperatures appear to be more sensitive, they are not recommended on unknown samples due to the small likelihood of returning false positives with *B. albiventris* and possibly other un-tested *Bactericera* spp.

It is recommended that this assay be performed at 60 °C – 62 °C, with a $MgCl_2$ concentration of 1.5mM and a primer concentration of 0.2 µM. To test the robustness of these conditions a multifactorial approach was taken [55]. The assay performed satisfactorily across the different treatments and was shown to be robust and unaffected by small changes in assay set-up (Supp Tab. S45). Each treatment gave 100% positives for amplification of *B. cockerelli* genomic DNA.

4. ***Discussion***

Formatted: Font: Bold, Italic, Underline

The Tomato-Potato psyllid is an economically damaging pest of solanaceous plants that has spread by human mediated dispersal. It causes feeding damage to plants but also is the major vector of '*Candidatus* Liberibacter solanacearum' (Lso), a phloem limited bacterium that is associated with disease in solanaceous and apiaceous plants. Management of this insect pest requires accurate identification of *B. cockerelli*, this is often difficult if eggs or immature life stages only are available for identification. Hitherto, identification of *B. cockerelli* required either considerable expertise in psyllid taxonomy or the lengthy process of DNA barcoding [54].

Formatted: Normal, No bullets or numbering

We have designed and validated the first species-specific, qualitative real-time PCR TaqMan assay for *B. cockerelli* by using the comparison of 73 non-target species to identify unique gene regions that were suitable for primer/probe design and species differentiation. The genus *Bactericera* currently contains 160 species [20] and <1% of these have been tested in the current study due to the difficulty in obtaining other specimens from the field or lab colonies. However Europe is home to 26 different species of *Bactericera* [20], 30% of which have been tested for false positives using this assay. Psyllid species that were tested are most commonly found in potato and carrot fields in Europe and the wider EPPO region which should minimize the potential for false positives and ensure the assay is efficient at detecting outbreaks in European ~~Th~~fields. The~~e~~ assay was also tested on nine~~9~~ closely related *Bactericera* species. The number of species used in our study is relatively high compared to other reported TaqMan assays for plant pests that report lower numbers of non-target species [56,57].

The assay is based on a 187 bp region of the ITS2 gene~~region was found to be suitable for assay design~~which was suitable as it contained high inter~~ra~~specific variation consisting of

stretches of insertions and deletions (INDELs)~~, which are ideal diagnostic sites for primer and probe design~~. ~~Although COI is classically used for species discrimination, mutations were less clustered which is likely due to third base degeneracy.~~ The ITS2 region ~~can also~~has been used to distinguish species phylogenetically and ~~has been used~~ to identify cryptic species in the *Cacopsylla pruni* complex [47]. DNA s~~The s~~equences obtained from this study will improve psyllid ~~the current species~~ representation on online DNA databases, reducing the chance of Type II errors (i.e. misidentification due to lack of conspecific references) [58]. The *B. cockerelli* sequences on which we tested this assay (and many of the non-target psyllid species) were from different geographic locations to account for intraspecific variation. *Bactericera cockerelli* specimens from the four USA biotypes and specimens from New Zealand all gave 100% true positives.~~It is important to note that the target and non-target specimens consisted of multiple samples of each species from different geographical regions. This reduced the risk of false negatives that may arise if during assay design, the target specimens were obtained from a limited geographic spread and were therefore not representative of the genetic variation within the species. To account for interspecific variation, *B. cockerelli* samples used in the assay validation consisted of specimens from the four USA biotypes and specimens from New Zealand.~~

~~Species identification can be achieved for *B. cockerelli* by sequencing the ITS2 or COI regions as both loci, to date, have entries in the National Centre for Biotechnology Information (NCBI) database. However, DNA sequencing is a lengthy process compared to real-time PCR, which in contrast is a rapid identification method involving fewer pieces of equipment, reagents, and time both for running the sample, and processing it digitally.~~ The success rates of eradications are dependent on the length of time between introduction, detection, and

implementation of eradication measures as Lso displays a short transmission time from *B. cockerelli* to potatoes [4,25]. Feasibly, methodology described in this study could be used to extract DNA from a specimen and test for *B. cockerelli* positives within 6-12 hrs or quicker. This is faster than identification by DNA barcoding and could aid in eradications/ prevention of incursions. This time could be reduced further if the real-time assay is used in conjunction with faster DNA extraction protocols.

~~The assay described in this study can identify *B. cockerelli* specimens from all life stages. It is robust, reliable and can detect 200 copies (0.00001 ng DNA) of the ITS2 gene region. This is the first assay which uses a TaqMan real-time PCR module to specifically identify this important pest species. The assay performs well across a range of annealing temperatures, $MgCl_2$ concentrations and primer concentrations and is a robust tool that can be used to give reliable results despite human error, different lab practices, equipment, standard operating procedures or PCR set-ups.~~

~~The assay can be used on eggs and immatures, as well as adults of *B. cockerelli,* which will enable the rapid identification of this species from specimens that may be otherwise impossible to identify. For example, identification of eggs of *B. cockerelli* is not possible using classical taxonomy methods alone and it is now possible to rapidly identify this cryptic life stage. In addition, adult females and immatures are considerably more difficult to identify compared to adult males. The genus *Bactericera* currently contains 160 species [20] and <1% of these have been tested in the current study due to the difficulty obtaining other specimens from the field or lab colonies. However Europe is home to 26 different species of *Bactericera* 30% of which have been validated using this assay [20]. This assay has been tested against the *Bactericera* species which are most commonly found in potato and carrot~~

Formatted: Font: Italic

fields in Europe and the wider EPPO region which should minimize the potential for false positives and ensure the assay is efficient at detecting outbreaks in European fields. There are currently no methods described within the EPPO “agreed diagnostic protocol for identification of *B. cockerelli”* [4]. In addition, the current EPPO control system for *B. cockerelli* and Lso [4] highlights the importance of identifying psyllid eggs and immatures on various plant materials during inspections and monitoring but gives minimal guidelines for achieving this. Validation of this assay demonstrates that it would be a reliable and accurate tool for use in this area and it will therefore be prepared for consideration by the EPPO diagnostic panel. This assay is also useful for monitoring *B. cockerelli* occurrence at several spatial scales, from local border checks to regional surveys which use different trapping methods (water, sticky, suction, aerial balloon traps) where no host plant data is available. Given the sensitivity of this assay it should be possible to detect *B. cockerelli* DNA from insect fragments (e.g. legs, heads) if DNA extraction is adequate. However, further validation should be performed to ensure the assay performs adequately on samples obtained from different traps. This assay should be tested on additional congeneric species and other closely related Triozidae psyllids. Another limitation of this assay is that it cannot yet be taken out into the field, making it less portable than LAMP assays or other NGS sequencing techniques such as Nanopore technology.

The ability to identify insects to species accurately and quickly is essential to support national biosecurity measures for the prevention and early detection of new pests and vectors of disease. Psyllids can be difficult to identify and certain species of concern, such as *B. cockerelli,* require skilled specialist taxonomists to accurately differentiate between closely related species that may or may not be of risk to plant health. Entomologists with

the skills required to do so are a valuable yet sparse resource and incorrect identification of a psyllid species could lead to a delay in national response or inappropriate allocation of resources to resolve a problem that is not present, possibly leading to the unnecessary destruction of crops. We have designed a functional tool for reliable, rapid, robust and accurate identification of the tomato-potato psyllid *B. cockerelli* to support national biosecurity measures should this pest be found within the EPPO region or other regions where it could become damaging to agriculture. The situation in New Zealand and recent interceptions of *B. cockerelli* on aubergine in plant material imported into the UK from Mexico highlight the risk and pathways for introduction into new areas [42]. Furthermore immature stages of *B. cockerelli* are commonly intercepted on vegetable material from the Western-United States and Mexico and adults are occasionally found on leafy vegetables such as lettuce [42]. Recent studies suggest *B. cockerelli* can utilize multiple host-plants within the Solanaceae and other plant families; the potential for eggs and immatures of *B. cockerelli* to be present on yet undetermined host-plants is likely.

The identification of psyllids using classical taxonomy is often aided using information about the host-plant on which the specimen was found [59]. However, although psyllids are typically monophagous, some species feed on a few closely related plant species. For example, northern hemisphere pest species, such as *B. cockerelli* and *B. trigonica,* feed on several plant species within a family [60]. In the case of *B. cockerelli* its range of host-plants (reproductive, food and incidental) is much wider than originally thought [9,16,61,62]. Adult psyllids may also settle on plants that they do not feed on, known as a casual plant [62]. It is possible that: A) *B. cockerelli* can utilise other host-plants and could be overlooked; B) other psyllid species morphologically/ecologically similar to *B. cockerelli* could be mistaken for this

pest if only considering the host plant on which they are found. Therefore, the identification of suspected host-plant material alone cannot be reliably used for psyllid identification and for most psyllid species their host-plant range is unknown or fragmentary at best. This assay can be used to study life parameters of *B. cockerelli* in the field such as oviposition on reproductive host-plants, as eggs can now be identified.

Additionally, host-plant data is sometimes unavailable depending on the trapping method employed. For example, indirect host-plant data is available from trapping methods at ground level (e.g. water/sticky traps) with less data available for insects caught beyond the vegetation layer (e.g. 12.2m suction traps or aerial balloon traps). Unlike spot checks performed by inspectors directly on plant produce, canopy-level or aerial traps which provide data at field or regional level [63,64]. A diagnostic assay for *B. cockerelli* is therefore useful for monitoring its occurrence at several spatial scales, from local border checks to regional surveys. As some collection methods obtain specimens where host-plant data is not available, this tool enables rapid screening of psyllids from suction-traps or those collected in sticky traps, pan traps or similar. However, further validation should be performed to ensure the assay performs adequately on *B. cockerelli* fragments obtained from these kinds of traps. The assay can also be used in areas currently known to have *B. cockerelli* to easily quantify numbers from the field or to monitor migration, distribution and spread of this pest species.

Further validation of this assay however should include its use in several different laboratories with different practitioners. Different reaction mixes should be tested for their suitability with this assay and its compatibility with alternative qPCR machines. It is also

~~valuable to test these primers in a set-up using an inter-collating dye such as Sybr Green. Additionally, this assay should be tested on further samples of *Bactericera* and other closely related Triozidae psyllids. Further validation of this assay should be performed on more *Bactericera* species not included in this study and on DNA from Solanaceous host plants. This will ensure false positives are not obtained from DNA extracted from complex matrices. Due to being based on real-time PCR chemistry, one limitation of this assay is that it cannot be taken out into the field, making it less portable than a LAMP assay or other NGS sequencing techniques such as Nanopore technology.~~

In conclusion a rapid, specific, robust, repeatable and reliable real-time PCR assay has now been validated and can be used to detect the important pest *B. cockerelli*. This will be ~~work has developed~~ an important tool for ~~detection of this pest and will~~ providinge much-needed support to prevent new outbreaks ~~of this pest~~. The assay can be implemented by practitioners with molecular biology experience and does not require personnel to have classical taxonomic knowledge of insects or psyllids; making this tool more accessible than traditional methods. The assay can be used to complement field surveillance and may facilitate further ecological studies of *B. cockerelli*~~.~~ requiring the identification of immatures and eggs~~that were previously not possible as they required the identification of immatures and eggs in the field or were too time consuming without this assay~~. The strength of this assay lies in the collaboration of molecular biologists and classical taxonomists working together to build a reliable database for DNA barcoding of psyllids.

6. Funding

This work was supported by the EU Horizon2020 Programme under grant agreement No. 635646, POnTE (Pest Organisms Threatening Europe) and the Scottish Government [RRL/001/14]. The Rothamsted Insect Survey, a National Capability, is funded by the Biotechnology and Biological Sciences Research Council under the Core Capability Grant BBS/E/C/000J0200. The authors declare there is no conflict of interest regarding the publishing of this article.

7. Acknowledgements

We thank A. Fereres & C. A. Antolínez Delgado (Institute of Agricultural Sciences, CSIC, Spain), A. Nissinen (Natural Resources Institute Finland), J. Munyaneza, R. Cooper, M. Heidt, K. Swisher Grimm (USDA Agricultural Research Services), S. Bulman (Plant and Food Research, New Zealand), A. Jensen, S. Halbert (Florida Department of Agriculture & Consumer Services, Dept. of Plant Industry) and Alberto Flores (Universidad Autónoma Agraria Antonio Narro) for specimens; and thank C. Jeffries, L. Webster, V. Mulholland, and A. Reid (SASA) for providing advice. We also thank SASA Potato Genotyping team for providing Potato DNA.

8. Author Contributions

JS-K: assay design and validation, investigation, performed analysis, collected data, bioinformatics, visualization, writing- original draft, supervision, project administration; **MJS:** assay design and validation, investigation, performed analysis, collected data, bioinformatics, visualization, writing- original draft, supervision, project administration; **YA:** investigation, performed analysis, validation, data curation, collected data; **MC:** provided resources, data analysis, curation of data, investigation, taxonomy expertise; **FH:** conceived the project, methodology, supervision, writing- original draft; **DO:** provided resources, data

analysis, curation of data, taxonomy expertise, writing – review and edit; **AG:** provided resources, data analysis, taxonomy expertise, writing- review and edit; **JB:** conceived the project, provided resources, methodology, writing- review and edit, supervision, project administration; **RS:** provided resources, data analysis, methodology; **DK:** conceived the project, conceptualization, funding acquisition, methodology, supervision, project administration.

9. References

1. Munyaneza JE, Sengoda VG, Crosslin JM, Garzon-Tiznado JA, Cardenas-Valenzuela OG. First report of “*Candidatus* Liberibacter solanacearum” in tomato plants in Mexico. Plant Dis. 2009;93: 1076. doi:10.1094/PDIS-93-10-1076A

2. Wallis RL. Ecological studies on the Potato Psyllid as a pest of potatoes. USDA Tech Bull. 1955;1107: 25.

3. Hansen AK, Trumble JT, Stouthamer R, Paine TD. A new huanglongbing species, “*Candidatus* Liberibacter psyllaurous,” found to infect tomato and potato, is vectored by the psyllid *Bactericera cockerelli* (Sulc). Appl Environ Microbiol. 2008;74: 5862–5865. doi:10.1128/AEM.01268-08

4. EPPO. PM 9/25 (1) *Bactericera cockerelli* and ‘*Candidatus* Liberibacter solanacearum.’ EPPO Bull. 2017;47: 513–523. doi:10.1111/epp.12442

5. Liefting LW, Perez-Egusquiza ZC, Clover GRG, Anderson JAD. A new ‘*Candidatus* Liberibacter’ species in *Solanum tuberosum* in New Zealand. Plant Dis. 2008;92: 1474–1474. doi:10.1094/pdis-92-10-1474a

6. Liefting LW, Sutherland PW, Ward LI, Paice KL, Weir BS, Clover GRG. A new ‘

Candidatus Liberibacter' species associated with diseases of solanaceous crops. Plant Dis. 2009;93: 208–214. doi:10.1094/PDIS-93-3-0208

7. Munyaneza JE. Zebra Chip disease of potato: Biology, epidemiology, and management. Am J Potato Res. 2012;89: 329–350. doi:10.1007/s12230-012-9262-3

8. Crosslin JM, Hamm PB, Eggers JE, Rondon SI, Sengoda VG, Munyaneza JE. First report of Zebra Chip disease and "*Candidatus* Liberibacter solanacearum" on potatoes in Oregon and Washington State. Plant Dis. 2012;96: 452. doi:10.1094/PDIS-10-11-0894

9. Kaur N, Cooper WR, Duringer JM, Badillo-Vargas IE, Esparza-Díaz G, Rashed A, et al. Survival and development of Potato Psyllid (Hemiptera: Triozidae) on Convolvulaceae: Effects of a plant-fungus symbiosis (Periglandula). PLoS One. 2018;13: 1–19. doi:10.1371/journal.pone.0201506

10. Knowlton G, Thomas W. Host plants of the Potato Psyllid. J Econ Entomol. 1934;27: 547.

11. Pletsch D. The Potato Psyllid *Paratrioza cockerelli* (Sulc), its biology and control. Bull Mont Agric Exp Stn. 1947;446: 1–95.

12. Wallis RL. Potato Psyllid selection of host plants. J Econ Entomol. 1951;44: 815–817. doi:10.1093/jee/44.5.815

13. Butler CD, Trumble JT. The Potato Psyllid, *Bactericera cockerelli* (Sulc) (Hemiptera: Triozidae): life history, relationship to plant diseases, and management strategies. Terr Arthropod Rev. 2012;5: 87–111. doi:10.1163/187498312X634266

14. Prager SM, Esquivel I, Trumble JT. Factors influencing host plant choice and larval

performance in *Bactericera cockerelli*. PLoS One. 2014;9. doi:10.1371/journal.pone.0094047

15. Martin NA. Host plants of the Potato / Tomato Psyllid : A cautionary tale. Weta. 2008;35: 12–16.

16. Cooper WR, Horton DR, Miliczky E, Wohleb CH, Waters TD. The weed link in Zebra Chip epidemiology: suitability of non-crop Solanaceae and Convolvulaceae to Potato Psyllid and "*Candidatus* Liberibacter solanacearum." Am J Potato Res. 2019; 262–271. doi:10.1007/s12230-019-09712-z

17. Swisher KD, Henne DC, Crosslin JM. Identification of a fourth haplotype of *Bactericera cockerelli* (Hemiptera: Triozidae) in the United States. J Insect Sci. 2014;14: 1–7. doi:10.1093/jisesa/ieu023

18. Swisher KD, Munyaneza JE, Crosslin JM. High resolution melting analysis of the Cytochrome Oxidase I gene identifies three haplotypes of the Potato Psyllid in the United States. Environ Entomol. 2012;41: 1019–1028. doi:10.1603/EN12066

19. Swisher KD, Sengoda VG, Dixon J, Echegaray E, Murphy AF, Rondon SI, et al. Haplotypes of the potato psyllid, *Bactericera cockerelli*, on the wild host plant, *Solanum dulcamara*, in the pacific Northwestern United States. Am J Potato Res. 2013;90: 570–577. doi:10.1007/s12230-013-9330-3

20. Ouvrard D. 2018 Psyl'list - The World Psylloidea Database. 2019. Available: http://www.hemiptera-databases.com/psyllist

21. Teulon DA., Workman PJ, Thomas KL, Nielsen MC. *Bactericera cockerelli*: Incursion,

dispersal and current distribution on vegetable crops in New Zealand. New Zeal Plant Prot. 2009;62: 136–144.

22. Australian Government Department of Agriculture and Water Resources. Detection of *Bactericera cockerelli* (Tomato-potato psyllid) in Western Australia. Canberra; 2017. Available: https://www.ippc.int/en/countries/australia/pestreports/2017/02/detection-of-bactericera-cockerelli-tomato-potato-psyllid-in-western-australia/

23. Munyaneza JE, Crosslin JM, Upton JE. Association of *Bactericera cockerelli* (Homoptera: Psyllidae) with "Zebra Chip" a New Potato Disease in Southwestern United States and Mexico. J Econ Entomol. 2007;100: 656–663. doi:10.1603/0022-0493(2007)100[656:AOBCHP]2.0.CO;2

24. Munyaneza JE, Fisher TW, Sengoda VG, Garczynski SF, Nissinen A, Lemmetty A. Association of "*Candidatus* Liberibacter solanacearum" With the Psyllid, *Trioza apicalis* (Hemiptera: Triozidae) in Europe. J Econ Entomol. 2010;103: 1060–1070. doi:10.1603/EC10027

25. Munyaneza JE, Sengoda VG, Stegmark R, Arvidsson AK, Anderbrant O, Yuvaraj JK, et al. First report of "*Candidatus* Liberibacter solanacearum" associated with psyllid-affected carrots in Sweden. Plant Dis. 2012;96: 453–453. doi:10.1094/PDIS-10-11-0871

26. Liefting LW, Weir BS, Pennycook SR, Clover GRG. "*Candidatus* Liberibacter solanacearum", associated with plants in the family Solanaceae. Int J Syst Evol Microbiol. 2009;59: 2274–2276. doi:10.1099/ijs.0.007377-0

27. Bextine B, Aguilar E, Rueda A, Caceres O, Sengoda VG, McCue KF, et al. First report of “*Candidatus* Liberibacter solanacearum” on tomato in El Salvador. Plant Dis. 2013;97: 1245. doi:10.1094/PDIS-03-13-0248-PDN

28. Munyaneza JE, Sengoda VG, Aguilar E, Bextine B, McCue KF. First report of “*Candidatus* Liberibacter solanacearum” on pepper in Honduras. Plant Dis. 2014;98: 154. doi:10.1094/PDIS-06-13-0598-PDN

29. Munyaneza JE, Sengoda VG, Aguilar E, Bextine BR, McCue KF. First report of “Candidatus Liberibacter solanacearum” infecting eggplant in Honduras. Plant Dis. 2013;97: 1654. doi:10.1094/PDIS-06-13-0641-PDN

30. Aguilar E, Sengoda VG, Bextine B, McCue KF, Munyaneza JE. First report of “*Candidatus* Liberibacter solanacearum” on tobacco in Honduras. Plant Dis. 2013;97: 1376. doi:10.1094/PDIS-04-13-0453-PDN

31. Munyaneza JE, Sengoda VG, Aguilar E, Bextine B, McCue KF. First report of “*Candidatus* Liberibacter solanacearum” associated with psyllid-infested tobacco in Nicaragua. Plant Dis. 2013;97: 1244. doi:10.1094/PDIS-03-13-0247-PDN

32. Johnson D. Canadian Potato Psyllid and Zebra Chip Monitoring Network Report. News Lett Can Potato Psyllid Zebra Chip Monit Netw. 2017; 1–19. doi:10.15713/ins.mmj.3

33. Castillo Carrillo C, Fu Z, Burckhardt D. First record of the Tomato Potato Psyllid *Bactericera cockerelli* from South America. Bull Insectology. 2019;72: 85–91.

34. Swisher Grimm KD, Garczynski SF. Identification of a new haplotype of ‘*Candidatus* Liberibacter solanacearum’ in Solanum tuberosum. Plant Dis. 2019;103: 468–474.

doi:10.1094/PDIS-06-18-0937-RE

35. Nelson WR, Fisher TW, Munyaneza JE. Haplotypes of “*Candidatus* Liberibacter solanacearum” suggest long-standing separation. Eur J Plant Pathol. 2011;130: 5–12. doi:10.1007/s10658-010-9737-3

36. Wen A, Mallik I, Alvarado VY, Pasche JS, Wang X, Li W, et al. Detection, distribution, and genetic variability of ‘*Candidatus* Liberibacter’ species associated with zebra complex disease of potato in North America. Plant Dis. 2009;93: 1102–1115. doi:10.1094/pdis-93-11-1102

37. Borges KM, Cooper WR, Garczynski SF, Thinakaran J, Jensen AS, Horton DR, et al. “*Candidatus* Liberibacter solanacearum” associated with the psyllid, *Bactericera maculipennis* (Hemiptera: Triozidae). Environ Entomol. 2017;46: 210–216. doi:10.1093/ee/nvw174

38. CNAS. Economic Impacts of Zebra Chip on the Texas Potato Industry. Texas; 2006.

39. Greenway G, Rondon SI. Economic impacts of Zebra Chip in Idaho, Oregon, and Washington. Am J Potato Res. 2018;95: 362–367.

40. Soliman T. Economic impact assessment of invasive plant pest in the European Union. Wageningen University, Wageningen, Netherlands. 2012.

41. DEFRA. Pest Alert :*Bactericera cockerelli*. 2017. Available: https://planthealthportal.defra.gov.uk/ph-api/pests/27077/notices/6682/documents/4183/document

42. EPPO. *Bactericera cockerelli*. EPPO Bull. 2013;43: 202–208. doi:10.1111/epp.12044

43. Burckhardt D, Ouvrard D. A revised classification of the jumping plant-lice (Hemiptera: Psylloidea). Zootaxa. 2012;34: 1–34.

44. Bell JR, Alderson L, Izera D, Kruger T, Parker S, Pickup J, et al. Long-term phenological trends, species accumulation rates, aphid traits and climate: Five decades of change in migrating aphids. J Anim Ecol. 2015;84: 21–34. doi:10.1111/1365-2656.12282

45. Sjolund MJ, Ouvrard D, Kenyon D, Highet F. Developing an RT-PCR assay for the identification of psyllid species. Proc Crop Prot North Britain. 2016; 279–282.

46. Percy DM. Radiation, diversity, and host-plant interactions among island and continental legume-feeding psyllids. Evolution (N Y). 2003;57: 2540–2556. doi:10.1111/j.0014-3820.2003.tb01498.x

47. Peccoud J, Labonne G, Sauvion N. Molecular test to assign individuals within the *Cacopsylla pruni* complex. PLoS One. 2013;8: 1–8. doi:10.1371/journal.pone.0072454

48. EPPO. PM 7/129 (1) DNA barcoding as an identification tool for a number of regulated pests. EPPO Bull. 2016;46: 501–537. doi:10.1111/epp.12344

49. Thompson JD, Higgins DG, Gibson TJ. CLUSTAL W: Improving the sensitivity of progressive multiple sequence alignment through sequence weighting, position-specific gap penalties and weight matrix choice. Nucleic Acids Res. 1994;22: 4673–4680. doi:10.1093/nar/22.22.4673

50. Altschul SF, Gish W, Miller W, Myers EW, Lipman DJ. Basic local alignment search tool. J Mol Biol. 1990;215: 403–410. doi:https://doi.org/10.1016/S0022-2836(05)80360-2

51. Clark K, Karsch-Mizrachi I, Lipman DJ, Ostell J, Sayers EW. GenBank. Nucleic Acids Res.

2015/11/20. 2016;44: D67–D72. doi:10.1093/nar/gkv1276

52. Untergasser A, Cutcutache I, Koressaar T, Ye J, Faircloth B, Remm M, et al. Primer3--new capabilities and interfaces. Nucleic Acids Res. 2012;40: e115. Available: http://www.ncbi.nlm.nih.gov/pmc/articles/PMC3424584/

53. Ye J, Coulouris G, Zaretskaya I, Cutcutache I, Rozen S, Madden T. Primer-BLAST: A tool to design target-specific primers for polymerase chain reaction. BMC Bioinfomatics. 2012;13: 134.

54. Liu D, Trumble JT, Stouthamer R. Genetic differentiation between eastern populations and recent introductions of Potato Psyllid (*Bactericera cockerelli*) into western North America. Entomol Exp Appl. 2006;118: 177–183. doi:10.1111/j.1570-7458.2006.00383.x

55. European Network of GMO Laboratories. Definition of minimum performance requirements for analytical methods of GMO testing. 2015. doi:10.2788/65827

56. Li D, Fan Q-H, Waite DW, Gunawardana D, George S, Kumarasinghe L. Development and validation of a Real-Time PCR assay for rapid detection of Two-Spotted Spider Mite, *Tetranychus urticae* (Acari: Tetranychidae). PLoS One. 2015;10: e0131887. doi:10.1371/journal.pone.0131887

57. Dhami MK, Dsouza M, Waite DW, Anderson D, Li D. Real-Time PCR assay for the identification of the Brown Marmorated Stink Bug (*Halyomorpha halys*). Front Mol Biosci. 2016;3: 1–6. doi:10.3389/fmolb.2016.00005

58. Virgilio M, Jordaens K, Breman FC, Backeljau T, de Meyer M. Identifying insects with

incomplete DNA barcode libraries, African fruit flies (Diptera: Tephritidae) as a test case. PLoS One. 2012;7. doi:10.1371/journal.pone.0031581

10. Supporting Information Captions

~~**Supplementary Table 1.** Information on non-target psyllid species and specimens tested using the *B. cockerelli* real-time PCR assay Bcoc_JSK2 showing number of technical replicates and false positives. All non-target species gave 0% false positives. GenBank Accession numbers are included for ITS2 and CO1 regions if sequencing was successful.~~

Supplementary Table 1~~2~~. Assay performance across a range of primer concentrations at 60 °C and 1.5mM $MgCl_2$. Optimum primer concentration was 0.2 µM showing the best combination of r^2, slope, efficiency, and sensitivity.

Supplementary Table 2~~3~~. Performance of *B. cockerelli* real-time PCR assay at different magnesium chloride ($MgCl_2$) concentrations.

Supplementary Table 3~~4~~. Summary of standard curves from optimisation of temperature on Bcoc_JSK2 real-time PCR assay for identification of B. cockerelli. All DNA concentrations tested above the limit of detection (10ng, 1 ng, 0.1ng, 0.01ng 0.001ng, 0.0001ng, 0.00001ng, 0.000001ng) ~~were~~ gave 100% positives across 3 x replicates. LOD is given for each temperature. All non-target Bactericera species tested at different DNA concentration gave 0% false positives except for B. albiventris cloned DNA which cross reacted at 64 and 66 °C. (*reactions at 64 °C gave 33.33% positives at 20 copy numbers).

Supplementary Table 4~~5~~. Set-up and results of multifactorial robustness experiment testing the Bcoc_JSK2 assay on *B. cockerelli* genomic DNA. All treatments showed 100% positives despite small changes to the overall set-up.

RESPONSE TO REVIEWERS

Dear Dr Sean Michael Prager,

Please find enclosed our revised manuscript entitled "A diagnostic real-time PCR assay for the rapid identification of the tomato-potato psyllid, *Bactericera cockerelli* (Šulc, 1909) and development of a psyllid barcoding database". We thank you and the reviewers for your careful review of our submitted manuscript and the opportunity to resubmit an improved version. We find the comments to be fair and constructive and have helped to improve the final manuscript. Please see below our responses to points raised by the reviewer's comments and the amendments we have made to the final manuscript. We provide a copy of the resubmitted manuscript with track changes and track changes accepted. Line numbers refer to those given in the resubmitted manuscript with track changes accepted.

We hope that you will consider this revised manuscript of a high enough standard to be published in PLOS ONE.

Yours Sincerely,

Dr Jason C Sumner-Kalkun

(on behalf of all co-authors)

EDITOR'S COMMENTS TO AUTHORS

15/01/2020

Editor: Sean Michael Prager, PhD
Dear Dr. Sumner-Kalkun,

Thank you for submitting your manuscript to PLOS ONE. After careful consideration, we feel that it has merit but does not fully meet PLOS ONE's publication criteria as it currently stands. Therefore, we invite you to submit a revised version of the manuscript that addresses the points raised during the review process.

This manuscript fell in a grey area between minor and major revisions. Three different reviewers examined the manuscript, and I also reviewed it. I agree with the 1st reviewer that you may be stretching a bit and could possibly focus some. This work will provide a useful tool. I think that alone makes it worth publication, and that opinion is shared by the reviewers. I also think that it is a complete and comprehensive piece of work. I, therefore, encourage you to focus on the comments from reviewer 1 and those about length etc. when preparing a resubmission.

We look forward to receiving your revised manuscript.

Kind regards,

Sean Michael Prager, Ph.D.
Academic Editor
PLOS ONE

Response:

- Thank you for considering our work for publication in your journal. We found all reviewer comments to be useful and constructive and appreciate you overall assessment of the manuscript. We are pleased to have the opportunity to resubmit an improved version. We have made considerable efforts to condense the introduction and discussion section to include only relevant information and to streamline the manuscript. We agree that there was some duplication and repetition in the discussion, and it has been re-written accordingly. We decided, due to the technical nature of the paper that a separate results and discussion section was more appropriate. We have addressed the reviewer concerns regarding data availability and all sequence data has been uploaded to GenBank and is now free to be made publicly available. We hope that our amendments are deemed adequate to meet the high standards of PLOS ONE and are excited about the possibility of publishing with you.

REVIEWER COMMENTS TO AUTHOR

Reviewer #1: This manuscript describes a qPCR assay to identify potato psyllid intercepted in shipments. The assay is paramount to Europe's ability to detect potential introductions of this psyllid, which would be harmful to agricultural production. The authors describe the assay and confirmed that it does not amplify the ITS gene of other psyllids. My major concern for the manuscript is that it is overwritten and over-interpreted. The study is very simple - qPCR assay to detect potato psyllid - yet the text is over 50 pages long, includes unrelated information in the introduction, and includes an overly long discussion. The manuscript should be re-written to focus only on the assay and its use in trade commodities. Specific comments are provided in an attached document. I will apologize for my handwriting.

MAJOR POINTS

We appreciate your thorough assessment of our manuscript and thank you for your time. We found your comments very constructive and helpful. We have taken the care to reduce the introduction and discussion sections considerably to provide more focus on the assay and its uses, removing a lot of the duplication. The manuscript has been edited down to 31 pages + supplementary material. We attempted to produce a combined results and discussion section but felt that, due to the technical nature of the paper, keeping these separate was preferable. We hope that you will agree with this assessment on reading the improved version.

On the recommendation of the reviewer on line 486 of the previous manuscript we have performed the assay on Potato DNA to check for cross-reaction. No false positives were obtained from 8x reps of 3 Potato samples "Maris Piper" variety.

MINOR POINTS

1. **Line 45: Abstract overwritten, stats to be removed, word count reduced**
- The abstract Line 21-39 has been reduced in size with all stats removed and is now within the word limit (252 words)

2. **Line 47: Remove "-" in "Potato-Psyllids"**
- Changed to "Potato Psyllid" now line 41

3. **Line 49: "The feeding of…." To be changed to "Feeding by"**
- Changed as suggested now line 43

4. **Line 53: Psyllid yellows refers to the feeding damage described above.**
- Removed to avoid confusion and improve accuracy. Line 47

5. **Lines 55-56: Change "…is also able to reproduce on…" to "…can also complete development on species of…."**
- Changed as suggested line 49-50

6. **Lines 56-58: Statement not deemed true**
- Statement removed line 51

7. **Line 61: Remove statement on Lso transmission to non-host plants of *B. cockerelli***
- Statement removed line 53

8. **Line 64-65: Remove claims about *B.cockerelli* populations observed to differ in their ability to spread Lso**
- Changed to: "Evidence suggests that these genetic types may differ in their ability to spread Lso…" Lines 56-57

9. **Line 86: Haplotype B is also found in *Bactericera maculipennis***
- Information added to the text line 71

10. Line 111: typo capsicum not italics

- Changed to "…*Capsicum*…" line 84

11. Line 223:This table is referenced a lot, make it a real table

- Supp Tab. S1 now changed to Table 1. In results section Line 244-250. Cited on lines: 244. Supp Tabs 2-4 renumbered to Supp Tabs 1-3 and Tables 1-3 renumbered to Tables 2-4.

12. **Line 276: Submitted to NCBI? Provide accession numbers**

- Accession numbers added to Table 1. Lines 246-252 and Table 4. Lines 271-276

13. Line 314-315: change "….cloned DNA as mentioned below." To "..DNA below".

- Changed to "….cloned DNA (see below)." Line 290

14. Line 319: change "immatures" to "nymphs"

- The term "immatures" is preferred by leading psyllid taxonomists Daniel Burckhardt and David Ouvrard, that latter of whom is an author on this paper. See ref: (Burckhardt et al. 2014). We have kept the term "immatures" or "immature life stages" throughout.

Burckhardt D, Ouvrard D, Queiroz D, Percy D (2014) Psyllid Host-Plants (Hemiptera: Psylloidea): Resolving a Semantic Problem. Florida Entomol 97:242–246 . https://doi.org/10.1653/024.097.0132

15. Line 411: "…Bactericera…" to be italicised

- Changed to italics. Line 354

16. Lines 439-441: Section to be re-written as inaccurate wording used

- This section was removed in the re-write of the discussion.

17. Line 468: Suggestion to perform further validation on Solanaceous DNA

- 3 x samples of *Solanum tuberosum* 'Maris Piper' were tested and were negative results added to lines: 194-196 and 284-285. Also results of primer blast etc. did not return any hits for *Solanum* species or any plant sequences.

<u>Reviewer #2</u>

- We are thankful to the reviewer for their detailed and careful examination of our paper. They have provided very useful, constructive comments regarding the technical aspects of the paper and have informed us of errors in the finer details. We hope we have incorporated changes to their satisfaction, and we have endeavoured to clear up the technical details that were missing or incorrect.

1. Data availability

- Psyllid DNA sequences have been uploaded to GenBank and accession numbers are provided in Tab1. And Tab4; lines 246-252 and 271-276 respectively.

2. Page 8 Line 163: What part of the body is used for micro-dissection to extract DNA? The authors should describe the micro-dissection procedure in more detail rather than only citing the papers.

- The non-destructive DNA extraction method is described on lines 121 – 132. "Micro-dissection" was used here to describe the piercing of the abdomen and thorax. "Micro-dissection" has been changed to "pierced" as a more appropriate term (line 126).

3. Page 8 Line 172: "For amplification of ITS2 primers CA55p8sFcm-F and CA28sB1d-R [60] and for amplification of CO1 gene regions arthropod barcoding Primers LCO1490 and HCO2198 [61]." The authors should check the grammar here. It is not a complete sentence. It could be "For amplification of ITS2, primers CA55p8sFcm-F and CA28sB1d-R [60] were used, and for amplification of CO1 gene regions, arthropod barcoding Primers LCO1490 and HCO2198 [61] were used."

- Changed as suggested lines 135- 137

4. **Page 10 Line 204: "DNA was extracted as above using the non-destructive method, amplified and cloned into competent Escherichia coli cells using the TOPO TA cloning kit (Thermo-Fisher)." The authors should specify what genes (ITS2 or CO1?) they amplified for cloning, and what restriction enzyme (EcoRI?) they used to linearize the plasmid.**

- Information added and moved from later section 2.5.2 Sensitivity. Now line 171-178

5. **Page 10 Line 212: The authors need to list the real time PCR cycling conditions here, for example XX degrees for XX seconds.**

- Added lines 178-181

6. **Page 10 Line 223: "All reactions with non-target psyllid DNA were run in conjunction with a TaqMan Exogenous Internal Positive Control Reagent Kit (Applied Biosystems) to ensure false positives were not obtained due to inhibition within the reaction". Here, "ensure" should be "rule out the possibility that"**

- Changed as suggested lines 196-201

7. **Page 11 Line 226: "DNA from all non-target psyllids was sequenced to ensure psyllid DNA was present in all reactions to rule out false negatives due to inefficient DNA extraction." What DNA was sequenced? PCR product from ITS2 or CO1? The authors need to specify**

- Details now added to new Tab 1 and citation to table included on lines 246-252

8. **Page 11 Line 239: "6 subsequent dilutions were made. Stock DNA 10 ng/µl was linearised using EcoRI restrictions enzyme (New England Biolabs)," Here "6 subsequent dilutions" should be "8 subsequent dilutions", according to the nine point 10-fold dilution series mentioned on Page 11 Line 236.**

- Corrected Line 212-213

9. **Page 12 Line 252: "A six point 1:10 dilution series starting at 10ng/µl was used with each dilution being performed in triplicate." Here, "six point" should be "nine point" according to Page 11 Line 236.**

- Only 6 points were used for repeatability. This is sufficient to analyse standard curves between runs. Lines 222-223 refer to sensitivity experiments only.

10. **Page 12 Line 263: "For each tested parameter, optimization was performed across an eight point 1:10 dilution series starting at 10ng DNA." Here, "eight point" should be "nine point", "10ng" should be "10ng/µl".**

- Corrected. Line 234

11. **In Supplementary table S1, green and red color coding should be explained in the text. What does TBC mean? Accession numbers should be given for all the sequences. Accession numbers in Table 3 should also be given and TBC should be explained.**

- We apologise for this error; this colouring has been removed as was an artefact of preparing the table and shouldn't have been included in the submitted version. TBC was used to show we were waiting for accession numbers. Accession numbers are now added to tables and TBC removed. Tab. 1 lines: 246-247 Tab.4 lines:

12. **Page 14 Line 289: "CO1 genes showed higher similarity and generally less conserved and variable regions compared to ITS2 regions." Here "less conserved and variable" should be "less variable".**

- Corrected line 266

13. Page 17 Line 310: “0.2 µ/mol” should be “0.2 µM”.

- Corrected line 285

14. Page 18 Line 324: “The copy number calculator available at http://scienceprimer.com/copy-number-calculator-for-realtime-pcr was used.” Here a hyperlink should be created. According to the link and the formula given, 0.00001ng DNA equals 4.879×10000 copies, if length of gene region is considered 187bp (product length of ITS2 in real time PCR). However, the authors calculated that it equals to 200bp. Please double check the calculation.

- Limit of detection is actually 0.000001 ng DNA. This mistake of 10 fold higher amounts stated in the text was found throughout and in tables. We have now corrected them. The correct equation should be:

Number of Copies = (ng DNA(0.000001) x 6.022×10^{23}) ÷ ((length of plasmid 4656bp + cloned fragment 700bp) * 1×10^{9} * 660) = 170.36 copy numbers.

15. Page 18 Line 337: “At primer concentration, 0.5 µM the assay was less sensitive only amplifying up to 0.001 ng DNA.” It should be “At primer concentration 0.5 µM, the assay was less sensitive only amplifying up to 0.001 ng DNA.”

- Corrected. Lines 313-314

16. Page 18 Line 338: “At higher primer concentrations (0.5 and 1.0) the assay showed higher sensitivity” Here “(0.5 and 1.0)” should be “(1.0 µM)”.

- Corrected. Line 314

17. Page 19 Line 350: “The precision of the assay was lower at higher MgCl2 concentrations 6mM and 8mM (Supp Tab. S3).” Here “6mM and 8mM” should be “7.5mM and 9mM”.

- Corrected. Lines 326-327

18. Page 19 Line 354: “Reactions at 58 °C were 10 to 100-fold less sensitive than reactions at 58 °C.” Here it should be “Reactions at 58 °C were 10 to 100-fold less sensitive than reactions at 64 °C.”

- Corrected. Lines 330-331

19. Page 20 Line 367: “We have designed and validated the first species-specific, qualitative real-time PCR TaqMan assay for B. cockerelli by using the comparison of 73 non-target species to identify unique gene regions that were suitable for primer/probe design and species differentiation.” Here “qualitative” should be “quantitative”.

  - Changed to quantitative. Line 351

Reviewer #3

  - We thank the reviewer for their thoughtful assessment of our manuscript and are pleased that only minor corrections were found throughout. The corrections have improved the manuscript greatly and have ironed out some important technical errors.

We hope that our amendments are deemed satisfactory and have covered the issues they have raised.

1. **Page 8 line 175: please replace amount of primers used with final concentration of primers (or add this)**

- Added. Line 138

2. Page 10 line 213: please add cycling conditions of real time PCR, as done for CO1 and ITS2 amplification

- Added lines 178-181

3. Table 1: should include also B. cockerelli, so to include fragment size of amplicons for this species. In alternative, fragment sizes can be added to the main text

- *B. cockerelli* added to table 2. Line 262-263

4. Table 3: not clear what the "/" symbol in the CO1 column means

- Samples with / were not amplified in this region. Accession numbers for each sample have been added and this is explained better in the text. Lines: 252 Tab.1 ; 276 Tab. 4

5. Page 17 line 310: please check spelling of concentration

- Corrected to µM. Line 285

6. Page 17 line 316: numbers seem not to add up: how many technical replicates were used per sample?

- Information on technical reps is incorporated into table 4. Some samples were tested in duplicate, triplicate or 6x replicates.

7. Page 18 line 323: I have tried the formula myself using the concentration (0.00001 ng) and fragment size (187 bp) specified by the authors, but I get a quite different number of ITS2 copies (about 50,000 versus 200). Please double check, and add actual numbers to the formula.

- Limit of detection is actually 0.000001 ng DNA. This mistake of 10-fold higher amounts stated in the text was found throughout and in tables. We have now corrected them. The correct equation should be:

Number of Copies = (ng DNA(0.000001) x 6.022×10^{23}) ÷ ((length of plasmid 4656bp + cloned fragment 700bp) * 1×10^{9} * 660) = 170.36 copy numbers.

8. Of some concern is the author's answer to the data accessibility question. Authors stated that they are not going to make all data available, with a generic "Some restrictions will apply". Please explain what data will not be made accessible and why.

- All data will be made available. Accession numbers were not available at the time of submission as they were restricted by one or more of our projects until we had consent to upload them to public databases.
