# Supplementary material for: A diagnostic real-time PCR assay for the rapid identification of the tomato-potato psyllid, Bactericera cockerelli (Šulc, 1909) and development of a psyllid barcoding database
Source: PLoS One. 2020 Mar 26;15(3):e0230741. doi: 10.1371/journal.pone.0230741 (PMC7098582; doi:10.1371/journal.pone.0230741)
Supplement: Attachment — Submitted filename: Response to Reviewers_v2.docx [file pone.0230741.s008.docx]

**RESPONSE TO REVIEWERS**

Dear Dr Sean Michael Prager,

Please find enclosed our revised manuscript entitled “A diagnostic real-time PCR assay for the rapid identification of the tomato-potato psyllid, *Bactericera cockerelli* (Šulc, 1909) and development of a psyllid barcoding database”. We thank you and the reviewers for your careful review of our submitted manuscript and for deeming the manuscript worthy of publication with some small amendments. We provide a copy of the resubmitted manuscript with track changes and track changes accepted. Line numbers refer to those given in the resubmitted manuscript with track changes accepted.

We are glad to be publishing our work in this journal as it will enable us to reach a wide-ranging scientific audience.

Yours Sincerely,

Dr Jason C Sumner-Kalkun

(on behalf of all co-authors)

**EDITOR’S COMMENTS TO AUTHORS**

04/03/2020

PONE-D-19-35047R1
A diagnostic real-time PCR assay for the rapid identification of the tomato-potato psyllid, Bactericera cockerelli (Šulc, 1909) and development of a psyllid barcoding database.
PLOS ONE

Dear Dr. Sumner-Kalkun,

Thank you for submitting your manuscript to PLOS ONE. After careful consideration, we feel that it has merit but does not fully meet PLOS ONE’s publication criteria as it currently stands. Therefore, we invite you to submit a revised version of the manuscript that addresses the points raised during the review process.

==============================
I appreciate the effort you have taken in both addressing the reviewer concerns and revising this work. In that context, I have rendered a decision of minor changes. This is primarily to give you the opportunity to make or address the additional reviewer comments.

We look forward to receiving your revised manuscript.

Kind regards,

Sean Michael Prager, Ph.D.
Academic Editor
PLOS ONE

Response:

- Thank you for considering our work for publication in your journal. We thank all reviewers for taking the time to carefully consider our manuscript. All comments have improved the manuscript greatly. We are pleased that only minor edits are necessary and otherwise the manuscript has been deemed suitable for publication in your journal.

**REVIEWER COMMENTS TO AUTHOR**

Reviewer #1:

The revised version of this manuscript is a substantial improvement from the original submission. I have only a handful of minor suggestions in the attached PDF that the authors might consider.

Response:

We thank the reviewer for their careful assessment of the manuscript and the comments previously made to help us to improve and streamline the manuscript. We have incorporated most minor changes and edits as detailed below.

**MINOR POINTS**

Line 21: Remove “many”

- Removed. Line 21 (of document with track changes accepted).

Line 24: You can keep central america if you want to, but technically, Central America is a region within North America, so all you really need to say is "North America"

- Reduced to just “North America”. Line 24

Line 25: add “and is considered an threat for introduction in Europe and other pest-free regions.”

- Added: “…; and is considered a threat for introduction in Europe and other pest-free regions.” Lines 24- 25

Lines 27-30: remove “successfully” and restructure sentence to remove “(100% n=X)” after each sample type.

- Sentence restructured and percentages and sample numbers removed. Lines 27-30.

Lines 58-61: I think you need to find a way to combine this short paragraph with the preceding paragraph.

- Paragraph added to a previous paragraph. Lines 48-51

Lines 67-72: Since the manuscript focusses on potato psyllid, I don't think you need such an in-depth discussion about the various Liberibacter haplotypes

- We would prefer to keep the information about Lso haplotypes in as we believe it is an important aspect of Lso epidemiology. The information on Lso haplotypes is greatly reduced compared to the previous manuscripts.

Line 77: change “North-West” to “Northwest”

- Changed. Line 76

Line 108: Change “North-Western” and “South-Western” to “Northwestern” and “Southwestern”.

- Changed. Line 107

Line 114: US collections of non-targets isn't mentioned in abstract.

- Specimens were collected by Andy Jensen from multiple locations in the USA and tested with the assays as detailed in Table 1. We have added “USA” to the abstract. Line 32.

Lines 129-132: “DNA extraction in DNeasy Blood and Tissue Kit Protocol from Animal Tissues (Qiagen)” mentioned previously on line 128?

- Information condensed to avoid duplication. Lines 128-130
